# Geographical Variations in Prostate Cancer Outcomes: A Systematic Review of International Evidence

**DOI:** 10.3389/fonc.2019.00238

**Published:** 2019-04-08

**Authors:** Paramita Dasgupta, Peter D. Baade, Joanne F. Aitken, Nicholas Ralph, Suzanne Kathleen Chambers, Jeff Dunn

**Affiliations:** ^1^Cancer Research Centre, Cancer Council Queensland, Brisbane, QLD, Australia; ^2^Menzies Health Institute Queensland, Griffith University, Southport, QLD, Australia; ^3^School of Mathematical Sciences, Queensland University of Technology, Brisbane, QLD, Australia; ^4^School of Public Health and Social Work, Queensland University of Technology, Brisbane, QLD, Australia; ^5^Institute for Resilient Regions, University of Southern Queensland, Toowoomba, QLD, Australia; ^6^St Vincent's Private Hospital, Toowoomba, QLD, Australia; ^7^School of Nursing & Midwifery, University of Southern Queensland, Toowoomba, QLD, Australia; ^8^Health and Wellness Institute, Edith Cowan University, Perth, WA, Australia; ^9^Faculty of Health, University of Technology, Sydney, NSW, Australia

**Keywords:** prostate cancer, rural, area-disadvantage, health disparity, systematic review, geographical variations, continuum of care

## Abstract

**Background:** Previous reviews of geographical disparities in the prostate cancer continuum from diagnosis to mortality have identified a consistent pattern of poorer outcomes with increasing residential disadvantage and for rural residents. However, there are no contemporary, systematic reviews summarizing the latest available evidence. Our objective was to systematically review the published international evidence for geographical variations in prostate cancer indicators by residential rurality and disadvantage.

**Methods:** Systematic searches of peer-reviewed articles in English published from 1/1/1998 to 30/06/2018 using PubMed, EMBASE, CINAHL, and Informit databases. Inclusion criteria were: population was adult prostate cancer patients; outcome measure was PSA testing, prostate cancer incidence, stage at diagnosis, access to and use of services, survival, and prostate cancer mortality with quantitative results by residential rurality and/or disadvantage. Studies were critically appraised using a modified Newcastle-Ottawa Scale.

**Results:** Overall 169 studies met the inclusion criteria. Around 50% were assessed as high quality and 50% moderate. Men from disadvantaged areas had consistently lower prostate-specific antigen (PSA) testing and prostate cancer incidence, poorer survival, more advanced disease and a trend toward higher mortality. Although less consistent, predominant patterns by rurality were lower PSA testing, prostate cancer incidence and survival, but higher stage disease and mortality among rural men. Both geographical measures were associated with variations in access and use of prostate cancer-related services for low to high risk disease.

**Conclusions:** This review found substantial evidence that prostate cancer indicators varied by residential location across diverse populations and geographies. While wide variations in study design limited comparisons across studies, our review indicated that internationally, men living in disadvantaged areas, and to a lesser extent more rural areas, face a greater prostate cancer burden. This review highlights the need for a better understanding of the complex social, environmental, and behavioral reasons for these variations, recognizing that, while important, geographical access is not the only issue. Implementing research strategies to help identify these processes and to better understand the central role of disadvantage to variations in health outcome are crucial to inform the development of evidence-based targeted interventions.

## Introduction

Worldwide, prostate cancer is the second most commonly diagnosed invasive cancer and the fifth leading cause of cancer deaths in men (1). Prostate cancer is especially prevalent in developed regions including Australia, United States and Western Europe, with incidence rates varying more than 25-fold between high and low incidence countries (1, 2). In contrast, mortality rates are higher in less developed countries especially among predominantly black Caribbean and sub-Saharan African populations (1, 2). These wide variations in the global burden of prostate cancer reflect the impact of country-specific differences in Prostate Specific Antigen (PSA) testing practices (2, 3), in addition to westernized diet, sedentary lifestyle and obesity (2), genetic differences (2, 3), dissimilar health systems, population life expectancy and competing causes of mortality (4).

Wide and persistent geographical disparities in prostate cancer incidence and mortality (5), treatment (6) and survival have been identified globally (7). Our previously published review (4) of international patterns in disparities along the prostate cancer continuum from detection to incidence, staging, treatment, survival and mortality identified a consistent pattern of poorer outcomes with increasing residential disadvantage and for rural residents. Specifically, men from rural and socioeconomically disadvantaged areas had lower rates of PSA testing, incidence, survival, and access or use of services, but also more aggressive disease at diagnosis and higher mortality rates (4). Although it is likely that the country-specific factors mentioned above also contribute to these within-country differences, a complex interplay of clinical, social, environmental, and behavioral factors are probably also involved (4–7).

Our earlier review (4) was limited in that it was not systematic, considered only a limited number of reference databases, and, given the extent of recent literature on this topic, no longer provides a contemporary summary of international patterns in prostate cancer. Here, we update and extend this work by systematically reviewing current international evidence on the extent of geographical variation in prostate cancer outcomes, with a focus on patterns by rurality and area-level socioeconomic disadvantage. Cancer outcomes along the continuum from PSA testing to diagnosis to mortality and survival are included. This review is intended to identify gaps in knowledge, formulate strategic research priorities and inform the development of evidence-based interventions to address observed inequities.

## Methods

### Patient Involvement

No patients were directly involved in the development of the research questions, choosing the outcome measures of interest, study design and implementation or interpretation of results.

### Definitions of Geographical Measures

The studies included in this systematic review used a range of definitions to define rurality and residential disadvantage. For the purposes of this review, “urban” areas were those described as “urban,” “metropolitan,” or “major cities,” with the remainder being categorized as “rural” areas. Advantaged areas were those described as “affluent” or “advantaged,” with the remainder being “disadvantaged.” Where studies reported on geographical variations by intermediate categories, such as suburban groups or quintiles of area-disadvantage, only comparisons between most “extreme” rural and/or most disadvantaged to the least rural and/or disadvantaged categories (such as very remote vs. metropolitan and quintile one vs. quintile five), are presented here.

### Clinical Questions

This review was conducted according to published PRISMA guidelines for conducting systematic reviews (8). Clinical questions to guide the review were clearly defined following a structured framework and agreed upon before commencing the review process. The review addressed six key questions on variations by residential location encompassing the key themes of PSA testing, prostate cancer incidence, tumor characteristics, survival, access to treatment services and prostate cancer mortality (Table 1).

**Table 1:** Clinical questions guiding the systematic review.

PSA testing 1. Internationally, do men living in more remote areas have lower rates of PSA testing for prostate cancer than men living in more urban areas? 2. Internationally, do men living in more socioeconomically disadvantaged areas have lower rates of PSA testing for prostate cancer than men living in more affluent areas? Prostate cancer incidence 3. Internationally, is there evidence of inequality in the incidence of prostate cancer according to rurality of residence? 4. Internationally, is there evidence of inequality in the incidence of prostate cancer according to socio-economic status of residence? Tumor characteristics 5. Internationally, among men diagnosed with prostate cancer, do men living in more remote areas have more advanced tumor characteristics than men living in more urban areas? 6. Internationally, among men diagnosed with prostate cancer, do men living in more socioeconomically disadvantaged areas have more advanced tumor characteristics than men living in more affluent areas? Prostate cancer survival 7. Internationally, among men diagnosed with prostate cancer, do men living in more remote areas experience poorer survival than men living in more urban areas? 8. Internationally, among men diagnosed with prostate cancer, do men living in more socioeconomically disadvantaged areas experience poorer survival than men living in more affluent areas? Access and use of treatment services 9. Internationally, among men diagnosed with prostate cancer, do men living in more remote areas access less prostate cancer-related treatment services than men living in more urban areas? 10. Internationally, among men diagnosed with prostate cancer, do men living in more socioeconomically disadvantaged areas access less prostate cancer-related treatment services than men living in more affluent areas? Prostate cancer mortality 11. Internationally, do men living in more remote areas have higher mortality due to prostate cancer than men living in more urban areas? 12. Internationally, do men living in more socioeconomically disadvantaged areas have higher mortality due to prostate cancer than men living in more affluent areas?

These themes and the relevant questions were repeated when considering differences by residential disadvantage.

### Literature Searches

The electronic databases: PubMed, EMBASE, CINAHL, and Informit were systematically searched for all indexed articles from 1 January 1998 to 30 June 2018. Final searches were undertaken on 02 July 2018. The Web of Science database was used for cited reference searches.

Search strategies were based on keywords and subject headings to reflect the review aim (Supplemental File 1). Key terms related to prostate cancer, e.g., “prostate neoplasms”; and “prostate cancers” were combined with terms pertaining to geographical aspects and area-based disadvantage, including “geographic inequalities,” “spatial,” “health services accessibility” and “rural health”; and outcome measures of interest, such as “PSA screening,” “incidence,” “stage,” “mortality,” “survival,” “prostatectomy,” “brachytherapy,” and “therapy.” Additional synonyms reflecting each of the key terms were also included.

### Inclusion Criteria

Studies were eligible if they met the following inclusion criteria:

1. the population included adult male prostate cancer patients or focused on a prostate cancer specific sub-group; and
2. the outcome measure was PSA testing, prostate cancer incidence, stage at diagnosis, survival, access and use of treatment services, or prostate cancer mortality
3. was a quantitative study on geographical differences by:
  - location of residential area (rural vs. urban, non-metropolitan vs. metropolitan comparisons); and/or
  - socioeconomic status of residential location

The scope of the review was limited to English language peer-reviewed original research articles. Reviews, editorials, books, conference abstracts and commentaries were excluded, although when identified through the systematic searches their reference lists were examined for relevant articles.

### Review Process

After removing duplicates, the titles and abstracts of all articles generated by the queries were independently reviewed by two reviewers (first PD, second PB) to assess their relevance to the clinical question(s). Full text versions of all articles of potential relevance were then retrieved for more detailed evaluation by one reviewer (PD). During this process, articles were categorized as “include” or “exclude” with reasons for exclusion being noted.

### Critical Appraisal

The quality of all included articles was evaluated using pre-defined criteria (9, 10). Quantitative studies were assessed using a modified (11) version of the Newcastle-Ottawa Scale (NOS) (10), a risk of bias assessment tool for non-randomized studies. The NOS assesses cohort studies on six items over five broad perspectives (a) selection bias; (b) ascertainment of exposure and/or confounders; (c) outcome assessment; (d) follow-up and (e) adjustments for residual confounders (two items). Case-control studies are evaluated on selection of cases and controls, ascertainment of exposure, response rate, adequacy of case-definition and accounting for residual confounders.

The NOS was further modified, by incorporating three additional items evaluating (a) study attrition (missing data), (b) statistical methods, and (c) data presentation, based on published checklists (9). Studies were scored according to the extent that they met each of the nine assessed criteria (Supplemental File 2) using an ordinal scale to rate the risk of bias as 0 (high), 1 (intermediate) and 2 (low) and the individual item scores then summed to give a total quality score. Each article was then assigned a total score (range of 0–18) which was categorized as “high” (12–16), “moderate” (9–13.5), or “low” (< 9) quality. Studies were not excluded based specifically on their quality rating.

Studies were also classified according to the published levels of evidence for quantitative observational studies from the Australian National Health and Medical Research Council (NHMRC) (17) in decreasing order of strength as Level I, Level II, Level III-1, Level III-2, Level III-3 or Level IV.

### Data Extraction

Information on study features including bibliography (author(s), year, title), setting, time period, design, population (such as sample size, eligibility criteria), outcome measures, assessed geographical unit(s), measure used to define urban/rural residence and/or area disadvantage (if applicable), relevant statistical results and key findings were extracted from all included articles by one reviewer. A selection of these were randomly checked by another reviewer. Disagreements were resolved by discussion until consensus was reached. Any discrepancies were rechecked with the original source.

## Results

### Study Selection

**Figure 1:**
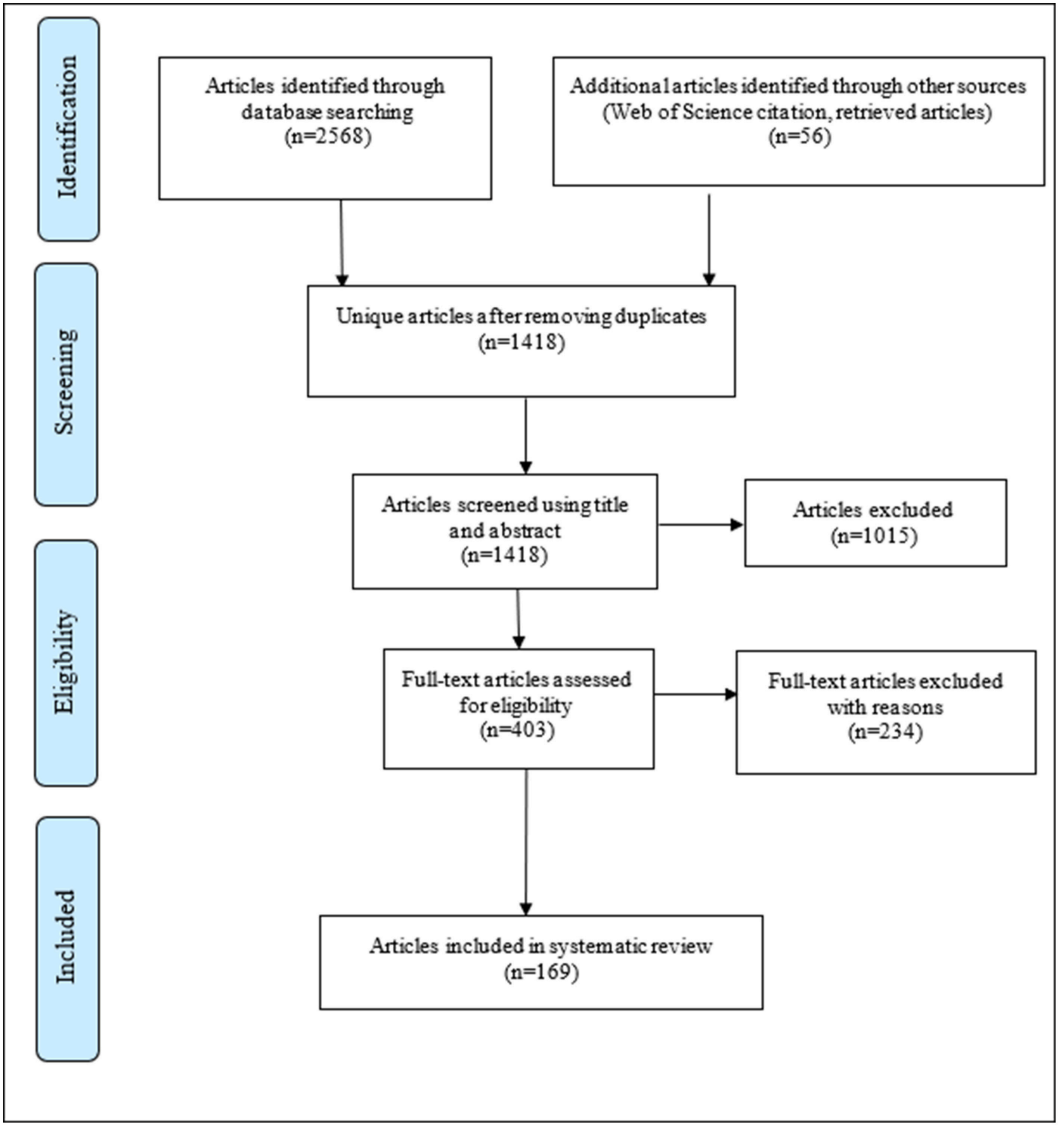
Process of inclusion and exclusion of studies for the systematic review.

The process of identifying relevant articles for the review is shown in a PRISMA diagram (Figure 1). A total of 2,568 articles were identified across combined databases with another 54 citations found through other sources (citation searches, reference lists, other reviews) After removing duplicates, 1,418 articles remained of which 1,015 were excluded after initial scanning of the title/abstracts. Following assessment of the remaining 403 full-text articles, 169 met the inclusion criteria for the review. Of the 234 excluded articles, most (more than 95%) did not specifically present quantitative statistics on geographical variations in the outcomes of interest, and/or used only individual-level instead of area-level measures of socioeconomic disadvantage.

### Study Characteristics

Around half (18) of the included studies were from the United States (USA), followed by Australia (19), the United Kingdom (UK, 24), Canada (5), Spain (4), the Netherlands (3), New Zealand (3), Denmark (2), France (2), and Ireland (2). Seven more studies were also from Europe (one each from Belgium, Finland, Germany, Greece, Lithuania, Sweden, and Switzerland), five from Asia (two from Japan, one each from Iran, South Korea and Taiwan), two from other parts of North America (French West Indies, Puerto Rico), one from South America (Colombia), one from Africa (Egypt) and one including Australia and Canada (Supplemental File 3).

Data for 157 (93%) of the included studies were sourced from administrative collections, such as population-based state or national cancer registries, official census and mortality records or non-population based clinical databases. The remaining 12 studies involved medical record reviews and cross-sectional surveys. With respect to study design, the majority (160, 95%) were observational cohort studies, eight were cross-sectional and one was a case-control study.

Of the 169 studies, 46 reported rural/urban differences in at least one of the outcome measures of interest, 82 assessed variations by residential disadvantage while 41 assessed both these measures. Overall, 87 studies looked at differences by residential rurality and 123 by disadvantage, respectively. Studies varied widely in the definition of urban or rural residence. While more than half (62%) of the relevant 87 studies used standardized definitions, such as the USA Department of Agriculture's Rural Urban Continuum Codes (RUCC) and Rural Urban Commuting Area Codes (RUCA) (12, 20) or the Accessibility/Rurality Index of Australia (ARIA+) (13) others defined non-urban and urban areas based on distances to services, degree of urbanization, population size or density. Three studies did not provide detailed information regarding how the geographical classification was derived (Supplemental File 3).

A range of measures were also used to define residential disadvantage (Supplemental File 3). More than three-quarters (76%) of the 63 non-USA-based studies used a standardized definition, such as the Australian Index of Relative Socio-Economic Disadvantage (IRSD) (14), Scottish Index of Multiple Deprivation (SIMD) (15), French Deprivation Index (16), or the UK-based Index of Multiple Deprivation (IMD) (21), Carstairs (22), or Townsend Deprivation Indexes (23). Of the 60 USA-based studies, 36 (60%) used various area-based indicators including median income, education or poverty while the remaining 24 studies created study-specific composite scores derived from these area-based measures.

Around half (24) of all the included studies were graded as high quality and the remaining 85 as moderate quality. There were no studies assessed as low quality. The median quality score was 13.4 (interquartile range of 12–15). Contributing to this higher quality was that most studies (138, 82%) used population-based representative data sources, such as cancer registries and mortality records. The key limiting factors were that 31 (18%) studies did not use a population-based representative sample, while more than a third (61, 36%) did not adjust for confounders or presented only age-adjusted estimates by residential location. Although cancer registries and mortality databases provide representative, reliable and objective data, they present limited information on all plausible prognostic factors. The lack of information on the extent of follow-up also lowered study quality. No study provided Level I evidence, nearly three-quarters (71%) gave Level II evidence, one quarter (24%) Level III evidence and 5% Level-IV evidence (Supplemental File 3).

### Key Findings

**Figure 2:**
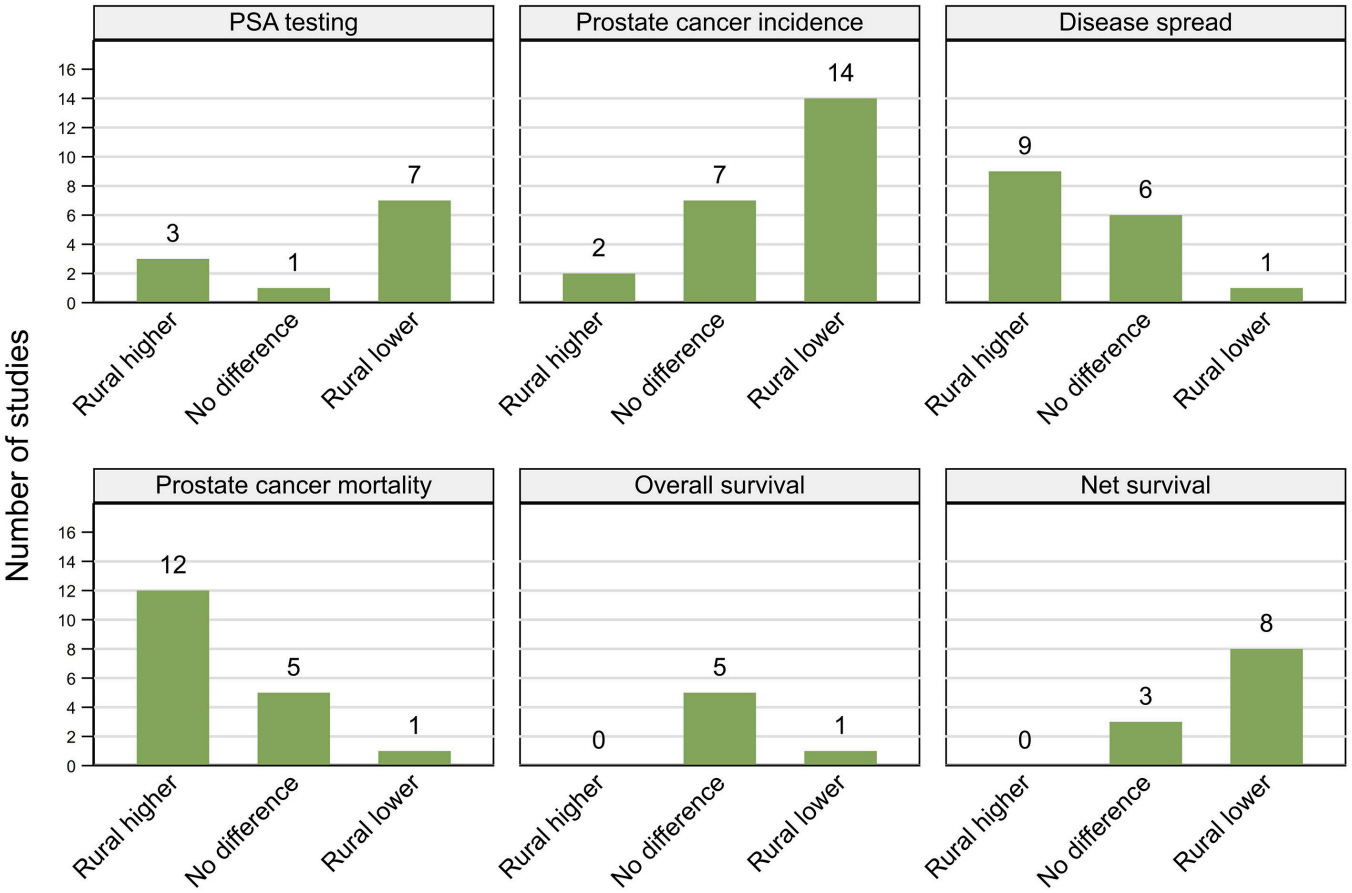
Summary of key patterns by residential rurality for PSA testing, prostate cancer incidence, disease spread, mortality, overall, and net survival.

**Figure 3:**
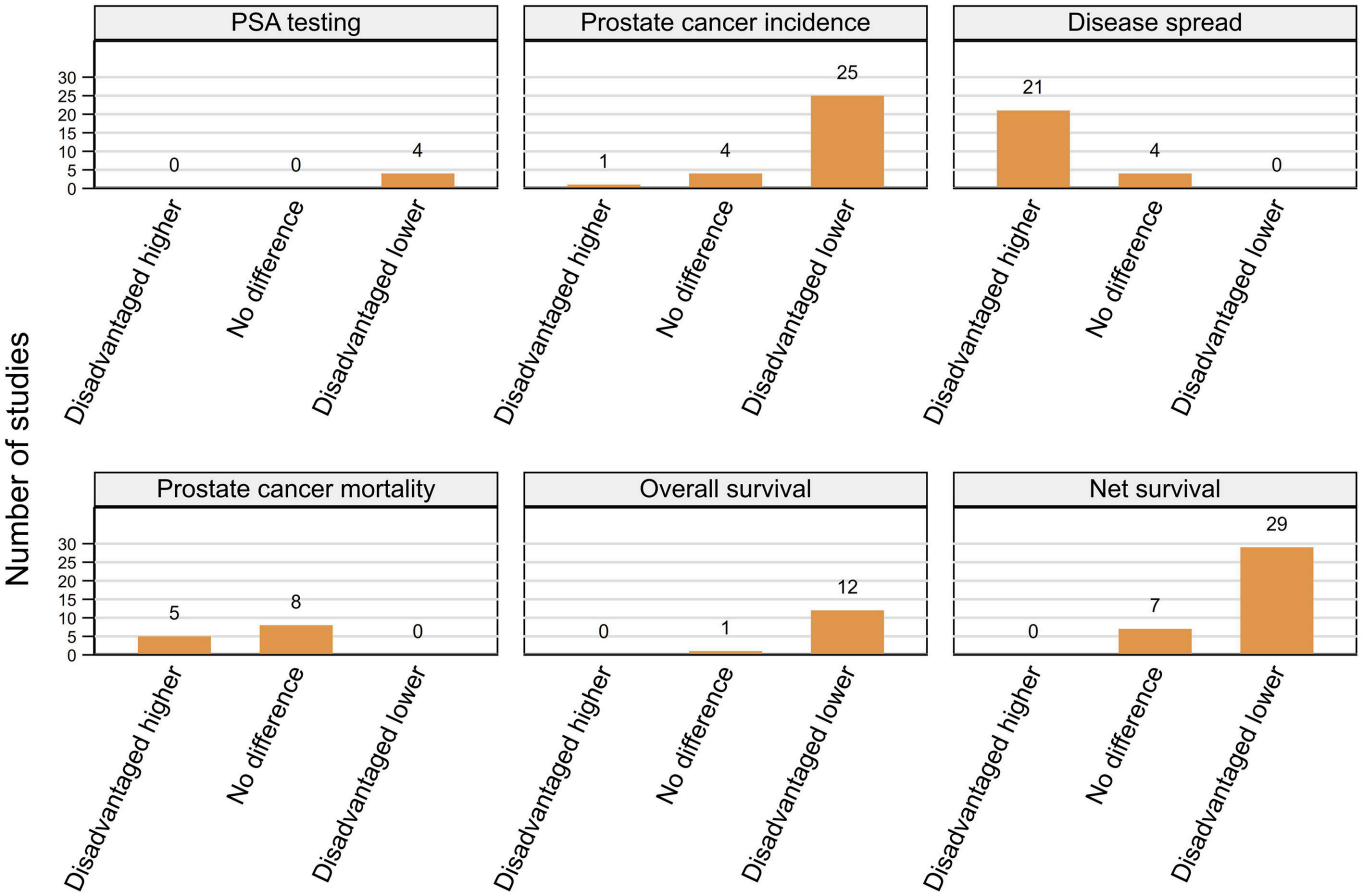
Summary of key patterns by residential disadvantage for PSA testing, prostate cancer incidence, disease spread, mortality, overall, and net survival.

**Figure 4:**
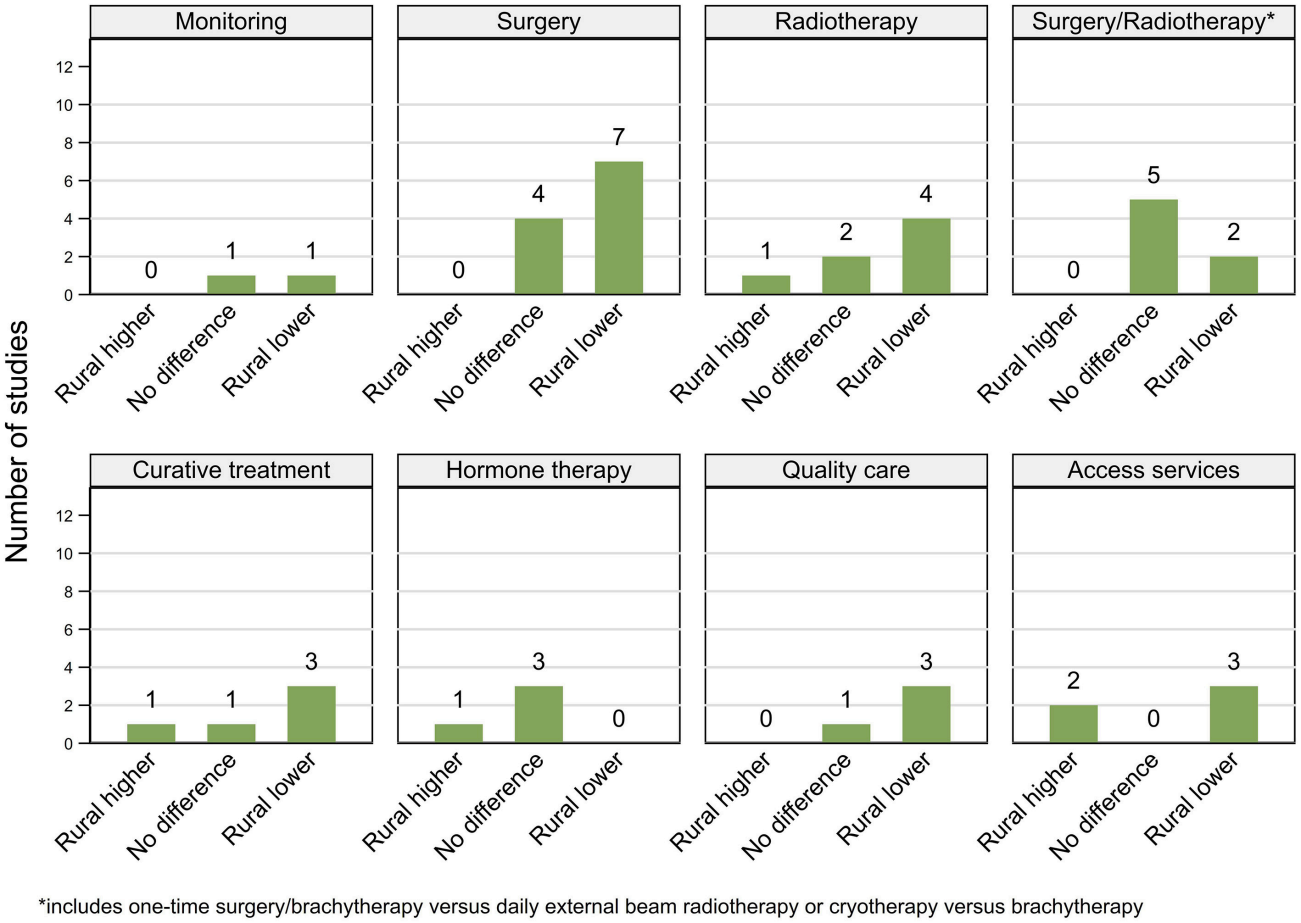
Summary of key patterns by residential rurality for access and use of prostate cancer related services.

**Figure 5:**
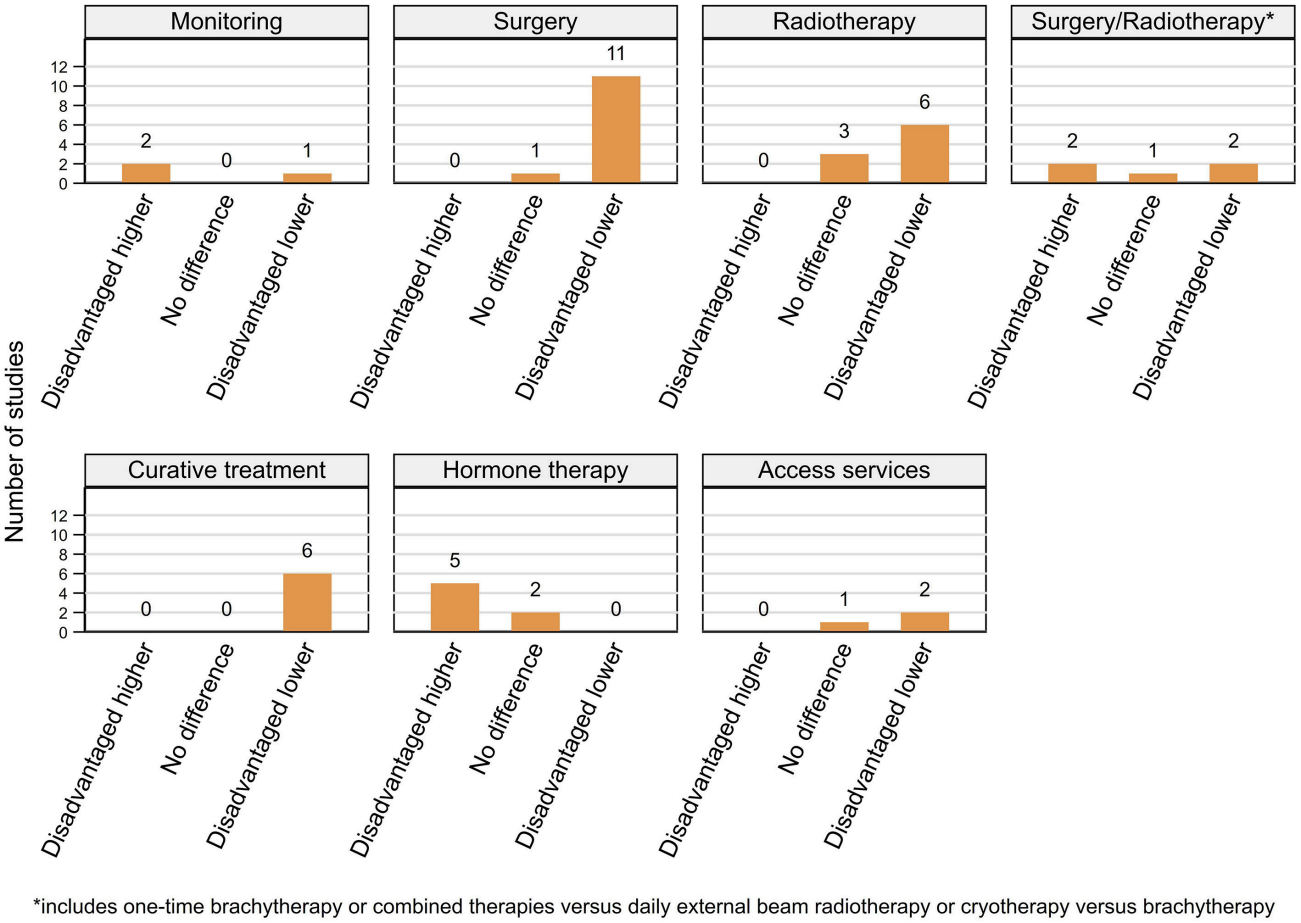
Summary of key patterns by residential disadvantage for access and use of prostate cancer related services.

Studies are summarized below (Figures 2–5; Tables 2–7) according to clinical questions within each of the key themes: (1) PSA testing, (2) incidence, (3) tumor characteristics, (4) survival, (5) access and use of services, and (6) mortality outcomes. Within each clinical question, results are presented separately for variations by rurality and residential disadvantage, further grouped by country within each of the continents (Africa, Americas, Asia, Europe and Oceania). Since several studies reported on multiple outcomes, some studies are repeated either in the same table or across multiple tables.

When multivariate analyses were conducted (136 of 169 studies), only the fully adjusted estimates are reported because many such studies only reported those results. Given the wide heterogeneity among studies in terms of the definitions of geographical measures, time periods, study populations, statistical methods and data presentation, only general trends within and among studies as well as between and within countries have been described. Given these limitations, any summary patterns have been deliberately interpreted with caution. The emphasis is on describing whether there was evidence of geographical variations in the relevant outcome by the reported measure of rurality and/or disadvantage, and if so, the direction and magnitude of the effect.

#### PSA Testing

##### Rurality

Results were not consistent across the 11 included studies (Figure 2; Table 2), both within and between countries. All studies included men aged at least 40 years. Based on the 2012 Behavioral Risk Factor Surveillance System (BRFSS) survey, two studies from the USA (one high, one moderate quality), both reported that asymptomatic non-Hispanic white men from rural areas were 11% more likely to undergo PSA testing than those from urban areas, although the geographical differences were not significant among other ethnic groups (26, 27). While urban residence was associated with lower prevalence of PSA testing in a high quality cross-sectional study using the 2010 BFRSS survey (19), the reverse pattern (higher PSA testing in urban areas) was reported by an earlier moderate-quality study, based on the 2001 BRFSS survey (25). Men from urban areas were also more likely to have repeated PSA tests within 3 years in another USA-based moderate quality study (28).

**Table 2:** Summary of included studies on differentials in PSA testing.

**References**	**Location**	**Period**	**Sample size^**a**^**	**Highest PSA testing**	**Findings [95% confidence interval in brackets]^**b, c**^**
**URBAN/RURAL DIFFERENTIALS (U, URBAN; R, RURAL)**					
Garg et al. (19)	USA	2010	108,245	Rural	OR (R:U) 1.22 [1.11–1.32]^d^
Jemal et al. (25)	USA	2001	NS	Urban	53% (R), 58% (U)^e, f^
Sammon et al. (26)	USA	2012	122,309	Rural (NHW) No difference (AA)	OR (R:U): NHW 1.11 [1.04–1.18]^d^ AA 1.06 [0.78–1.45]^d^
Trinh et al. (27)	USA	2012	30.3 million	Rural (NHW) No difference (Asian)	OR (R:U): Overall 1.11 [1.03–1.20]^d^ NHW 1.11 [1.03–1.19]^d^, Asian 2.17 [0.85–5.56]^d^
Zhu et al. (28)	MD, USA	2006	1,721	Urban (2 PSA tests in past 3 years)	OR (R:U) 0.77 [0.63–0.99]^d^
McAlister et al. (29)	Alberta, Canada	2012–2015	55,603	Areas with higher ratio of specialists to GP	OR (unit increase specialist/GP ratio) 7.79 [5.13–11.29]
Guessous et al. (30)	Switzerland	1992–2012	12,034	Urban (ever), Urban (past 2 years)	OR (ever, R:U) 0.90 [0.85–0.95] OR (past 2 years, R:U) 0.80 [0.72–0.88]
Littlejohns et al. (23)	UK	2006–2010	212,039	No difference	OR (R:U) 1.01 [0.98–1.04]
Baade et al. (31)	Australia	1995–2009	NS	Urban	2008–2009 Rate ratio (R:U) 0.93 [0.93–0.94]^g, h^
Coory and Baade (32)	Australia	1995–2003	NS	Urban	2002–2003 Rate ratio (R:U) 0.84 [0.83–0.85]^g, h^
Obertova et al. (33)	New Zealand	2010	34,960	Urban	RR (R:U) 0.72 [0.56–0.92]
**RESIDENTIAL AREA DISADVANTAGE DIFFERENTIALS (A, AFFLUENT; D, DISADVANTAGED)**					
Gorday et al. (34)	Calgary, Canada	2011	NS	Affluent	RR (per $100,000 increase income) 1.26 [1.20–1.32]
McAlister et al. (29)	Alberta, Canada	2012–2015	55,603	Affluent	OR (D:A) 0.85 [0.81–0.88]^d^
Littlejohns et al. (23)	UK	2006–2010	212,039	Affluent	OR (D:A) 0.84 [0.81–0.97]
Morgan et al. (35)	Scotland	2003–2008	96,484	Affluent	OR (D:A) 0.68 [0.64–0.71]^d^

*AA, African-American; GP, general practitioner; NHW, non-Hispanic white; MD, Maryland; NS, not stated; OR, Odds ratio; PSA, prostate-specific antigen; RR, relative risk; UK, United Kingdom; USA, United States*.

a *All studies only included men aged at least 40 years*.

b *Having had at least one PSA test over the study period*.

c *Findings based on model-based estimates adjusted for age and various socio-demographic factors, except where indicated*.

d *If the reference category was rural or disadvantaged, the inverse odds ratio or rate ratio was calculated to ensure consistency across Tables and make them easier to read*.

e *Findings based on descriptive statistics*.

f *Significant (p < 0.05)*.

g *Findings based on age-standardized rates (per 100,000 men) of PSA testing*.

h *Findings for other years provided in figures in article*.

A moderate quality study from the UK found no differences by urban/rural location in PSA testing (23), while three other (moderate quality) studies reported higher rates of PSA testing among men from urban areas in Australia (31, 32) and New Zealand (33). Consistent with this, urban residents were 10% more likely to have ever had PSA testing and 20% more likely to have been tested within the past 2 years from 1992 to 2012 in Switzerland (30), while men living in regions with higher ratio of specialists to general practitioners (GPs) were around eight times more likely to have PSA testing in Canada (29) (both studies moderate quality).

##### Residential disadvantage

All four (two high, two moderate quality) of the included studies reported that PSA testing was more common in affluent areas (Figure 2; Table 2). Among 212,039 men aged 40–69 years in the UK, men living in most disadvantaged areas were 16% less likely to have a PSA test between 2006 and 2010 than those from affluent areas (23). Similar patterns were reported by one Scottish (35) and two Canadian studies (29, 34).

#### Incidence

##### Rurality

Of the 23 studies included, 14 (six high, eight moderate quality) reported higher prostate cancer incidence rates in urban areas (25, 32, 36, 38, 40–44, 46, 47, 49, 52, 53), two (moderate) higher rates in rural areas (50, 55) and seven (one high, six moderate) no urban rural differences (31, 37, 39, 45, 48, 51, 54) (Figure 2; Table 3). Six studies used a mixture of data collected both prior to and after the widespread use of PSA testing, which combined with varying time-periods for analysis, could lead to conflicting patterns (31, 32, 39, 49, 51, 55).

**Table 3:** Summary of included studies on differentials in prostate cancer incidence.

**References**	**Location**	**Period**	**Sample size**	**Highest incidence**	**Findings [95% confidence interval in brackets]^**a, b**^**
**URBAN/RURAL DIFFERENTIALS (U, URBAN; R, RURAL)**					
Dey et al. (36)	Gharbiah, Egypt	1999–2002	NS	Urban	RR (R:U) 0.21 [0.16–0.27]^c^
Holowaty et al. (37)	Canada	1999–2003	NS	No difference	Maps in article
Altekruse et al. (38)	South-east USA	1999–2001	66,468	Urban (localized disease)	Maps in article^d^
Clegg et al. (39)	USA	1973–2001	1,995	No difference	RR (R:U) 1.05 [0.94–1.16]
Fogleman et al. (40)	USA	2001–2011	NS	Urban	ASR (R) 132.8, (U) 143.3^d, e^
Jemal et al. (25)	USA	1995–2000	NS	Urban	Rate ratio (R:U) 0.93 (W) 0.89 (AA) (CI not reported)^d, e^
Major et al. (41)	USA	1995–2006	23,612	Areas high density urologists (W) No difference (AA)	HR (low: high urologist density): W 0.90 [0.84–0.96] AA 1.11 [0.80–1.54]
Zahnd et al. (42)	USA	2009–2013	NS	Urban	ASR (R) 114.1, (U) 124.5^d, e^
Zahnd et al. (43)	USA	2009–2013	NS	Urban	NAACCR: ASR (R) 114.1 [113.5–114.7], (U) 124.5 [124.3–124.8]^d^ SEER 18: ASR (R) 117.8 [116.5–119.2], (U) 131.9 [131.3–132.4]^d^
Ghali et al. (44)	NH, USA	2004–2011	4,731	Urban	42.3% (R), 57.7% (U)^e, f^
Higginbotham et al. (45)	MS, USA	1996	1,501	No difference	ASR (R) 136.0, (U) 147.1 (*p* > 0.05, CI not reported)^d^
Oliver et al. (46)	VA, USA	1990–1999	37,373	Urban	RR (R:U) 0.20 (W), 0.26 (AA)^c, e, g^
Marsa et al. (47)	Denmark	1994–2003	8,279	Urban	RR (R:U) 0.86 [0.78–0.96]
Meijer et al. (48)	Denmark	2004–2008	14,612	No difference	HR (R:U) 0.95 [0.88–1.02]
Ocana-Riola et al. (49)	Granada, Spain	1985–1996	1,037	Urban	RR (R:U) 0.69 [0.61–0.79]^c^
Sharp et al. (50)	Ireland (RoI, NI)	1995–2007	2,550	Rural	RR (R:U) 1.06 [1.03–1.11]^c^
Jarup et al. (51)	UK	1975–1991	24,457	No difference	Maps in article
AIHW (52)	Australia	2004–2008	NS	Urban	ASR (R) 150.8 [142.9–159.0], (U) 173.3 [171.9–174.7]^d^
Baade et al. (31)	Australia	1986–2005	NS	No difference	Box 3, page 295 article^d^
Coory and Baade (32)	Australia	1985–2000	NS	No difference (until 1993) Urban (1994–2000)	1999–2000 Rate ratio (R:U) 0.95 [0.92, 0.98]^d^, Box 3, page 114 article
Cramb et al. (53)	QLD, Australia	1998–2007	NS	Urban	Figure 5, page 8 article
Depczynski et al. (54)	NSW, Australia	2006–2009	3,647	No difference	HR (R:U) 0.99 [0.86–1.14]^c^
Yu et al. (55)	NSW, Australia	1982–2007	68,686	Rural	65.8% (R), 37.9% (U)^e, f^
**RESIDENTIAL AREA DISADVANTAGE DIFFERENTIALS (A, AFFLUENT; D, DISADVANTAGED)**					
Boscoe et al. (56)	16 states and LA, USA	2005–2009	436,000	Affluent	Figure 1, page 2195 article
Hastert et al. (57)	USA	2000–2002	NS	No difference	HR (D:A) 0.87 [0.75–1.01]
Houston et al. (58)	USA	2009–2013	945,586	Affluent	ASR (A) 128.9 [125.9–132.0], (D) 119.5 [118.9–120.2]^d^
Kish et al. (59)	USA	2002–2008	357,078	Affluent	22.6% (A), 12.5% (D)^e, f^
Major et al. (41)	USA	1995–2006	23,612	Affluent (W) No difference (AA)	HR (unit increase disadvantage): W 0.88 [0.85–0.92] AA 1.04 [0.93–1.17]
Singh and Jemal (60)	USA	1988–1992	NS	Affluent	Rate ratio (D:A) 0.79 (all), 0.68 (W), 0.82 (AA) (CI not reported)^d, e^
Yu et al. (61)	USA	2000–2008	NS	Affluent	2006–2008 ASR (A) >80 (D) 60, (CI not reported)^d^ Figures, page 88 article
Cheng et al. (62)	CA, USA	1998–2002	98,484	Affluent	Rate ratio (D:A) 0.78 [0.77–0.80]^c, d^
Yin et al. (63)	CA, USA	2000–2002, 2006–2008	148,009–150,929	Affluent	RR (D:A) 0.75–0.82, (*p* < 0.001, CI shown in Figures 3, 4, page 88–89 article)
Liu et al. (64)	LA, USA	1972–1997	83,068	No difference (1972–1987), Affluent (1988–1997)	1972–1987 ASR (A) 78.0 [76.1–79.8], (D) 76.0 [74.1–77.9]; 1988–1995 (A) 167.9 [164.7–171.1], (D) 106.0 [103.0–109.1]; 1996–1997 (A) 173.6 [167.1–180.1], (D) 86.4 [81.2–91.6]^d^
Mather et al. (65)	Louisiana, USA	1988–1999	31,159	Affluent (W)	RR (unit increase disadvantage) 0.93 [0.90–0.97]
Oliver et al. (46)	VA, USA	1990–1999	37,373	Affluent (W), no difference (AA)	RR (D:A) 0.65 (W)^e^, 1.15 (AA) (CI not reported)^c, g^
Sanderson et al. (66)	SC, USA	2000–2002	407 (cases) 393 (controls)	Disadvantaged	OR (D:A) 1.92 [1.25–2.94]^c^
Luce et al. (67)	French West Indies	2009–2010	1,750	No difference	RR (D:A) 1.08 [0.91–1.29]
Soto-Salgado et al. (68)	Puerto-Rico	1992–2004	NS	Affluent	RR (D:A) 0.89 [0.83–0.96]^c^
Haddad-Khoshkar et al. (69)	Iran	2005–2008	10,361	Affluent	RR (unit increase advantage) 5.98 [3.47–8.53]
Miki et al. (70)	Japan	1990–2009	732	No difference	HR (D:A) 0.96 [0.69–1.33]^c^
Aarts et al. (71)	South-east Netherlands	1996–2008	12,706	Affluent	2008 ASR (A) 130 (D) 77 (CI not reported)^d^, Figure 3, page 2639 article
Meijer et al. (48)	Denmark	2004–2008	14,612	Affluent	HR (D:A) 0.79 [0.75–0.83]^c^
Pukkala and Weiderpass (72)	Finland	1971–1995	6,972	Affluent	SIR (A) 1.24 [1.15–1.32], (D) 0.86 [0.80–0.92]^d^
Bryere et al. (16)	Normandy, France	1997–2009	11,611	Affluent	RR (D:A) 0.84 [0.78–0.92]^c^
Garcia-Gil et al. (73)	Catalonia, Spain	2009–2012	NS	Affluent	RR (D:A) 0.74 [0.69–0.80]
Vicens et al. (74)	Girona, Spain	1993–2006	NS	Affluent	RR (D:A) 0.60 [0.47–0.76]
Ocana-Riola et al. (49)	Granada, Spain	1985–1996	1,037	Affluent	RR (D:A) 0.73 [0.57–0.93]
Jarup et al. (51)	UK	1975–1991	24,457	No difference	RR (D:A) 1.00 [0.96–1.05]
Maringe et al. (75)	England	1986–2004	87,102	Affluent	RR (D:A): SA 0.67 [0.58–0.77], non-SA 0.88 [0.87–0.89]
Morgan et al. (35)	Scotland	2003–2008	96,484	Affluent	OR (D:A) 0.68 [0.52–0.87]^c^
Shafique et al. (76)	Scotland	1991–2007	15,519	No difference (1991–1996) Affluent (1997–2007)	Rate ratio (D:A): 1991–1996, 0.99, 1997–2002, 0.79 2003–2007, 0.62^e^(CI not reported)^d^
Tweed et al. (15)	Western Scotland	2001–2012	15,314	Affluent	RR (D:A) 0.87 [0.76–0.96]
Cramb et al. (53)	QLD, Australia	1998–2007	NS	Affluent	Figure 5, page 8 article

*AA, African-American; ASR, age-standardized rate; CA, California; CI, confidence interval; HR, hazard ratio; LA, Los Angeles; MS, Mississippi; NAACCR, North American Association Central Cancer Registries; NH, New Hampshire; NI, Northern Ireland; NS, not stated; OR, Odds ratio; RoI, Republic of Ireland; RR, relative risk; SA, South-Asian; SC, South Carolina; SEER, surveillance epidemiology end results; SIR, standardized incidence ratio; UK, United Kingdom; USA, United States; VA, Virginia; W, White*.

a *Findings based on model-based estimates adjusted at least for age and geographical measure, except where indicated*.

b *Reference citations provided when authors gave only figures*.

c *If the reference category was rural or disadvantaged, the inverse odds ratio or rate ratio was calculated to ensure consistency across Tables and make them easier to read*.

d *Findings based on age-standardized incidence rates (per 100,000 men) and/or standardized incidence ratios*.

e *Significant (p < 0.05)*.

f *Findings based on descriptive statistics*.

g *Generated from reported coefficients*.

While eight of the USA-based studies, reported higher incidence rates among urban residents (or those from areas with higher density of urologists) (25, 38, 40–44, 46), two found no geographical differentials (39, 45). Similarly, two Australian studies reported no significant differences (31, 54), three studies found higher prostate cancer incidence among urban males (32, 52, 53) and one study recorded the reverse pattern (55). In Denmark, urban men had higher incidence rates from 1994 to 2003 (47), but no differential was evident from 2004 to 2008 (48).

##### Residential disadvantage

Prostate cancer incidence was consistently higher among men from affluent areas across 25 (11 high, 14 moderate quality) of the 30 included studies with only a single (moderate) USA-based case-control study reporting the reverse pattern (66) (Figure 3; Table 3). The remaining four (two moderate, two high quality) studies from the USA (57), UK (51), Japan (70), and French West Indies (67) found no differentials by residential disadvantage.

Analysis of the 1988–1992 Surveillance, Epidemiology, and End Results (SEER) data showed that residents of affluent areas had higher prostate cancer incidence than men from deprived areas (60), while rates decreased monotonically with increasing residential disadvantage in the USA from 2005 to 2009 (56). Similar patterns were reported by nine other USA-based (41, 46, 58, 59, 61–65), 11 European, (15, 16, 35, 48, 49, 71–76) and one study each from Australia (53), Puerto-Rico (68), and Iran (69).

#### Tumor Characteristics

There were some variations in the definition of advanced prostate cancer with three studies each basing their classification on tumor size (77, 78, 85) or prostate cancer risk groups (79, 86, 100) and one on pathological Gleason score (90). All remaining studies used a standard cancer staging system (such as the SEER Summary stage or TNM) with Stage I-II cancers consistently referred to as localized disease, Stage III as regional disease and distant/metastatic (Stage IV) cancers as advanced (18, 24, 25, 39, 58, 64, 84, 88, 92, 102), although some collectively categorized both regional and distant cancers as advanced disease (21, 41, 55, 80–83, 87, 89, 91, 93–99, 101).

##### Rurality

Findings were not consistent across the 16 included studies (Figure 2; Table 4), with nine (four high, five moderate quality) reporting more advanced tumor characteristics among rural men and six (three high, three moderate) no geographical differentials. However, one high quality study from New Zealand found that men who lived closer to cancer centers were more likely to have advanced disease (87). There were no clear patterns in study findings with characteristics, such as sample size or time period. While three Australian studies (83–85) found no evidence of geographical differentials, three others reported that rural men were more likely to be diagnosed with advanced disease (24, 55, 86). Further discrepancies in findings were evident across the nine USA-based studies, with six reporting more advanced disease among rural residents (25, 77, 81, 82) and those with poorer access to urologists (41, 79), whereas three others found no significant differences in the rates of advanced disease between urban and rural men (39, 78, 80).

**Table 4:** Summary of included studies on differentials in advanced stage prostate cancer.

**References**	**Location**	**Period**	**Sample size**	**Highest advanced cancer^a^**	**Findings [95% confidence interval in brackets]^b,c^**
**URBAN/RURAL DIFFERENTIALS (U, URBAN; R, RURAL)**					
Baldwin et al. (77)	USA	2004–2006	51,982	Rural	23.6% (R), 19.7% (U)^d, e^
Clegg et al. (39)	USA	1973–2001	2,457	No difference	OR (R:U) 1.08 [0.75–1.56]
Jemal et al. (25)	USA	1995–2000	NS	Rural	Rate ratio (R:U) 1.13 (W) 1.09 (AA) (CI not reported)^e, f^
Major et al. (41)	USA	1995–2006	23,612	No difference (W) Areas low density urologists (AA)	HR (low: high urologist density): W 0.98 [0.79–1.21] AA 2.68 [1.31–5.47]
Skolarus et al. (78)	USA	2008	11,333	No difference	34% (R), 32% (U) (*p* > 0.05)^d^
Holmes et al. (79)	NC, USA	2004–2005	2,251	Furthest from urologists Only metastatic (W)	OR (unit increase distance): W 1.03 [1.01–1.07] AA 1.09 [1.04–1.14]
McLafferty and Wang (80)	IL, USA	1998–2002	42,291	No difference	OR (R:U) 0.96 (*p* > 0.05, CI not reported)
Goovaerts and Xiao (81)	FL, USA	1981–2007	226,435	Rural^f^	Maps in article^f^
Xiao et al. (82)	FL, USA	1996–2002	60,289	Rural	OR (R:U) 1.14 [1.05–1.24]
Depczynski et al. (83)	NSW, Australia	2006–2009	1,005	No difference	OR (R:U) 1.03 [0.52–2.04]^g^
Luo et al. (24)	NSW, Australia	1993–2002	32,643	Rural	HR (R:U) 1.24 [1.14–1.36]
Tervonen et al. (84)	NSW, Australia	1980–2009	104,168	No difference	OR (R:U) 1.21 [0.88–1.66]
Yu et al. (55)	NSW, Australia	1982–2007	68,686	Rural	10.8% (R), 9.9% (U)^d, e^
Papa et al. (85)	Victoria, Australia	1995–2000	1,984	No difference	2.8% (R), 2.4% (U) (*p* > 0.05)^d^
Ruseckaite et al. (86)	Victoria, Australia	2008–2013	7,204	Rural	High-risk 26.0% (R), 24.0% (U), metastatic 11.8% (R) 5.2% (U)^d, e^
Haynes et al. (87)	New Zealand	1994–2004	25,078	Closest to cancer centers	OR (highest: lowest distance) 0.61^e^ (CI not reported)
**RESIDENTIAL AREA DISADVANTAGE DIFFERENTIALS (A, AFFLUENT; D, DISADVANTAGED)**					
Boscoe et al. (88)	16 states and LA, USA	2005–2009	436,251	Disadvantaged	RR (D:A) 1.27 [1.20–1.34]
Byers et al. (89)	7 States, USA	1997	4,332	Disadvantaged	13% (A), 16% (D)^d, e^
Chu et al. (90)	3 States, USA	1989–2010	2,502	Disadvantaged (income, poverty)	OR (D:A)^g^ Income 1.37 [1.03–1.79]; Poverty 1.47 [1.11–1.92] Employment 1.27 [0.96–1.67], Education 1.10 [0.85–1.43]
Greenlee and Howe (18)	USA	1997–2000	366,006	Disadvantaged	OR (D:A) 1.68 [1.50–1.88]
Houston et al. (58)	USA	2008–2013	NS	Disadvantaged (Age 50–74) No difference (Age ≥75)	Age 50–74, ASR (A) 9.2, (D) 15.4^e, f^ Age ≥75, ASR (A) 38.7, (D) 43.0
Major et al. (41)	USA	1995–2006	22,523	No difference	HR (unit increase disadvantage): W 1.13 [0.79–1.63] AA 0.90 [0.79–1.03]
Marlow et al. (91)	USA	1998–2004	687,464	Disadvantaged	OR (D:A) Income 1.08 [1.05–1.12], Education 1.09 [1.06–1.13]
Weiner et al. (92)	USA	2004–2013	1,034,754	Disadvantaged	OR (D:A) 1.39 [1.35–1.44]
Liu et al. (64)	LA, USA	1977–1997	NS	No difference (1977–1987), Disadvantaged (1987–1997)	Figure 3, page 708 article^f^
McLafferty and Wang (80)	IL, USA	1998–2002	42,291	No difference	OR (unit increase advantage) 0.92 (*p* > 0.05, CI not reported)
Niu et al. (93)	NJ, USA	1993–1999	41,999	Disadvantaged	32.2% (A), 34.2% (D)^d, e^
Schwartz et al. (94)	Detroit, USA	1988–1992	11,896	Disadvantaged	OR (D:A) 1.30 [1.14–1.47]^g^
Goovaerts et al. (95)	FL, USA	1981–2007	256,365	Disadvantaged	15.7% [15.1–16.3] (A), 21.2% [20.1–22.2] (D)^f^
Xiao et al. (96)	USA	1990–2001	167,386	Disadvantaged	OR (unit increase income) 0.98 [0.97–0.99] OR (unit increase education) 0.86 [0.78–0.96]
Xiao et al. (82)	FL, USA	1996–2002	60,289	Disadvantaged	OR (unit increase advantage) 0.81 [0.67–0.98]
Xiao et al. (97)	FL, USA	2002–2007	11,083	No difference	OR (per $1,000 increase income) 0.96 [0.92–1.01]
Aarts et al. (98)	South-east Netherlands	1998–2008	11,086	Disadvantaged	26% (A), 30% (D)^d, e^
Lyratzopoulos et al. (99)	UK	1998–2006	15,916	Disadvantaged	OR (D:A) 1.06 [1.03–1.09]
McVey et al. (100)	UK	2000–2006	43,222	Disadvantaged	71.4% (A), 76.5% (D)^d, e^
Lyratzopoulos et al. (21)	Eastern England	2006–2010	20,372	Disadvantaged	OR (D:A) 1.21 [1.12–1.30]
Maclean et al. (101)	England	2012	27,880	Disadvantaged	RD (D:A) 4.9 [2.9–7.0]^e, h^
Luo et al. (24)	NSW, Australia	1993–2002	32,643	Disadvantaged	HR (D:A) 1.12 [1.04–1.19]
Tervonen et al. (84)	NSW, Australia	1980–2009	104,168	Disadvantaged	OR (D:A) 1.33 [1.23–1.45]
Tervonen et al. (102)	NSW, Australia	2000–2008	47,401	Disadvantaged	OR (D:A) 1.95 [1.65–2.30]
Haynes et al. (87)	New Zealand	1994–2004	25,078	No difference	OR (D:A) 1.28 (*p* > 0.05, CI not reported)

*AA, African-American; ASR, age-standardized rate; CI, confidence interval; FL, Florida; HR, hazard ratio; IL, Illinois; LA, Los Angeles, NS, not stated; NC, North Carolina; NJ, New Jersey; NSW, New South Wales; OR, Odds ratio; RD, risk difference; SIR, standardized incidence ratio; UK, United Kingdom; USA, United States; W, White*.

a *Some measure of spread of disease, such as stage at diagnosis, clinical tumor size or prostate cancer risk groups*.

b *Findings based on model-based estimates adjusted at least for age and geographical measure, except where indicated*.

c *Reference citations provided when authors gave only figures*.

d *Findings based on descriptive statistics*.

e *Significant (p < 0.05)*.

f *Findings based on age-standardized rates (per 100,000 men) by stage*.

g *If the reference category was rural or disadvantaged, the inverse odds ratio or rate ratio was calculated to ensure consistency across Tables and make them easier to read*.

h *A positive value means higher likelihood of being diagnosed with advanced cancer vs. reference group*.

##### Residential disadvantage

A consistent pattern of advanced stage at diagnosis among men from disadvantaged areas was evident across 21 (15 high, six moderate quality) of the 25 included studies (Figure 3; Table 4) despite varying definitions of advanced prostate cancer. Four studies (two high, two moderate), three from the USA (41, 80, 97) and one from New Zealand (87) found no differences by residential disadvantage.

Analysis of 436,251 incident cases from the USA found that males from disadvantaged areas were 1.27 times more likely to be diagnosed with advanced prostate cancer from 2005 to 2009 (88). Similar patterns were reported by 12 other USA-based studies (18, 58, 64, 82, 89–96), although the differential was only evident after 1987 in one instance (64) and among men aged 50–74 years at diagnosis in another study (58). All three studies from Australia (24, 84, 102), four from the UK (21, 99–101), and the single Dutch study (98) also reported positive association between residential disadvantage and being diagnosed with advanced disease.

#### Survival

Any interpretation of patterns and comparability across studies should reflect the type of survival measure used and their respective definitions. Two commonly used measures are overall survival (deaths from all causes) or net survival (includes relative and cancer-specific survival) that is the mortality specifically associated with a cancer diagnosis (186). Patterns are presented separately for overall and net survival.

##### Rurality

Of six studies that looked at association between residential rurality and overall survival (Figure 2; Table 5), four (high quality) reported no geographical differentials for men diagnosed with prostate cancer in the USA after controlling for treatment and comorbidities (103–106). Similar patterns were reported by a single (moderate quality) Australian study (108) whereas men living closer to primary care had higher survival in a single (high quality) study from England that did not control for either treatment or comorbidities (107).

**Table 5:** Summary of included studies on differentials in prostate cancer survival.

**References**	**Location**	**Diagnostic interval (survival interval)**	**Sample size**	**Highest survival**	**Findings (survival or mortality risk, higher mortality risk is poorer survival) [95% confidence interval in brackets]^a, b, c, d, e, f^**
**URBAN/RURAL DIFFERENTIALS (U, URBAN; R, RURAL)**					
**Overall survival**					
Chen et al. (103)	USA	2004–2006 (2004–2012)	19,565	No difference, high-risk (s, t, c, d)	HR (R:U) 1.03 [0.86–1.26]
Marsh et al. (104)	USA	2004–2012 (2004–2012)	268,378	No difference, intermediate-risk (s, t, c, d)	HR (R:U) 0.97 [0.89–1.05]^g^
Prasad et al. (105)	USA	2004–2007 (2004–2007)	41,275	By NCCN PC risk groups No difference (t, c, d)	5-years HR (R:U): low 0.98 [0.76–1.25], intermediate 1.09 [0.93–1.28], high 0.91 [0.90–1.03]
Vetterlein et al. (106)	USA	2004–2012 (2004–2012)	775,999	No difference (s, t, c, d)	HR (R:U) 0.99 [0.94–1.04]
Jones et al. (107)	Northern England	1994–2002 (1994–2005)	20,688	Closest to primary care (d)	HR (unit increase travel time) 1.04 [1.01–1.07]
Hall et al. (108)	WA, Australia	1982–2001 (1982–2001)	14,123	No difference (t, c, d)	3-years HR (R:U) 0.71 [0.36–1.07]
**Relative survival**					
Marsa et al. (47)	Denmark	1994–2003 (1994–2006)	8,279	Urban	5-years RS (R) 48% [45–52], (U) 57% [55–60]^b^
Baade et al. (31)	Australia	1982–2004 (1982–2006)	NS	Urban (1990–2004)	5-years HR (R:U): 1982–1989, 1.01 [0.97–1.05], 1990–1999, 1.14 [1.11–1.17], 2000–2004, 1.24 [1.17–1.31]
Jong et al. (109)	NSW, Australia	1992–1996 (1992–1999)	NS	Urban (s)	5-years RER (R:U) 2.53 [1.60–4.01]
Yu et al. (55)	NSW, Australia	1982–2007 (1992–2007)	68,686	Urban (s, d)	10-years RER (R:U) 1.32 [1.19–1.46]
**Prostate-cancer specific survival**					
White et al. (110)	TX, USA	1995–2002 (1995–2003)	87,444	No difference (s, d)	HR (R:U) 0.95 [0.79–1.14]
Li et al. (111)	Sweden	1990–2008 (1990–2008)	73,159	Urban (c, d)	OR (R:U) 1.26 [1.19–1.35]
Campbell et al. (112)	Scotland	1991–1995 (1991–1995)	6,833	Closest to cancer centers (d)	HR (R:U) 1.23 [1.02–1.48]
Tervonen et al. (113)	NSW, Australia	1980–2008 (1980–2008)	95,543	Urban (s, d)	PCS SHR (R:U) 1.10 [1.05–1.16]
Thomas et al. (114)	QLD, Australia	2005–July 2007 (2005–2011)	7,393	No difference (s, t, d)	5-years PCS SHR (R:U) 1.21 [0.72–2.05]
Papa et al. (85)	Victoria, Australia	1995–2000 (1995–2008)	1,984	Urban, men who had RP (s, d)	10-years PCS SHR (R:U) 4.09 [1.56–10.7]
Tan et al. (115)	Victoria, Australia	2004–2014 (2004–2014)	14,686	No difference, men who had RP (s)	HR (R:U) 0.74 (*p* > 0.05, CI not reported)
**RESIDENTIAL AREA DISADVANTAGE DIFFERENTIALS (A, AFFLUENT; D, DISADVANTAGED)**					
**Overall survival**					
Chen et al. (103)	USA	2004–2006 (2004–2012)	19,565	Affluent, high-risk (s, t, c, r)	HR (D:A): income 1.23 [1.10–1.37]^g^, education 1.22 [1.09–1.37]^g^
Glaser et al. (116)	USA	2004–2013 (2004–2013)	113,719	By intermediate and high-risk groups Affluent (s, t, c)	HR (D:A): intermediate 1.21 [1.10–1.32]^g^, high 1.25 [1.15–1.35]^g^
Marsh et al. (104)	USA	2004–2012 (2004–2012)	268,378	Affluent, intermediate-risk (s, t, c, r)	HR (D:A): income 1.26 [1.19–1.34], education 1.14 [1.07–1.21]
Prasad et al. (105)	USA	2004–2007 (2004–2007)	41,275	By PC risk groups Affluent (intermediate, high-risk) No difference (low-risk) (t, c, r)	5-years HR (D:A): low 1.13 [0.84–1.50], intermediate 1.52 [1.25–1.85], high 1.16 [1.02–1.33]
Vetterlein et al. (106)	USA	2004–2012 (2004–2012)	775,999	Affluent (s, t, c, r)	HR (D:A): income 1.28 [1.23–1.34], education 1.13 [1.09–1.18]
Byers et al. (89)	7 States, USA	1997 (1997–2002)	4,332	No difference (s, c)	5-years HR (D:A) 1.14 [0.95–1.37]
DeRouen et al. (117)	San Francisco, USA	1997–2003 (1997–2009)	1,800	Affluent (s, t, c)	HR (D:A) 1.47 [1.04–2.08]
Hong et al. (118)	FL, USA	2001–2007 (2001–2012)	6,457	Affluent (s, t, c)	HR (per $1,000 increase income) 0.99 [0.98–0.99]
Xiao et al. (119)	FL, USA	2001–2007 (2001–2007)	18,042	Affluent (s, t, c)	HR (per unit increase income) 0.99^h^ (CI not reported)
Aarts et al. (98)	South-east Netherlands	1998–2008 (1998–2010)	11,086	By stage and age group^h^ Localized affluent (age 60–74) Advanced affluent (age ≥60) (t, c)	HR (D:A): Localized Age < 60 1.72 [0.90–3.31], Age 60–74 1.46 [1.22–1.74], Age ≥75 1.13 [0.96–1.34] advanced Age < 60 1.08 [0.65–1.77], Age 60–74 1.21 [1.01–1.46], Age ≥75 1.35 [1.10–1.64]
Jones et al. (107)	Northern England	1994–2002 (1994–2005)	20,688	Affluent (r)	HR (unit increase disadvantage) 1.06 [1.05–1.07]
Burns et al. (120)	RoI	1988–2009 (1988–2010)	26,816	Affluent (s)	OR (D:A) 1.20 [1.09–1.33]
Hall et al. (108)	WA, Australia	1982–2001 (1982–2001)	14,123	Affluent (t, c, r)	3-years HR (D:A) 1.34 [1.10–1.64]
**Relative survival**					
Mariotto et al. (121)	USA	1973–1995 (1973–1995)	NS	Affluent	5-years HR (increasing income) 1.03 (CI not reported)^i, j^
Bravo et al. (122)	Cali, Colombia	1995–2004 (1994–2006)	3,999	Affluent	5-years RER (D:A) 3.57 [2.37–5.40]
Ito et al. (123)	Osaka, Japan	1993–2004 (1993–2010)	7,922	Affluent	5-years RS, Deprivation gap (%) −15.3 [−19.3, −11.3]^f, j^
Belot et al. (124)	Western France	1997–2010 (1997–2013)	13,044	Affluent	5-years RER (unit increase disadvantage) 1.05 [1.02–1.08]
Jansen et al. (125)	Germany	1997–2006 (2002–2006)	132,559	Affluent	5-years RER (D:A) 1.55 [1.46–1.84]
Shack et al. (126)	UK	1986–2000 (1986–2004)	26,673	Affluent	5-years Deprivation gap (%) 1996–2000 −6.9 [−10.3, −3.4]^f, j^
Shafique and Morrison (127)	Scotland	1991–2007 (1991–2008)	15,292	Affluent (s)	5-years RER (D:A) 1.48 [1.31–1.68]
Shafique et al. (128)	Glasgow, Scotland	2000–2007 (2000–2008)	897	No difference (s)	5-years RER (D:A) 1.39 [0.61–3.18]
Coleman et al. (22)	England and Wales	1996–1999 (1996–2001)	73,921	Affluent	5-years Deprivation gap (%) 1996–1999 −7.2 [−9.0, −5.5]^f, j^
Rowan et al. (129)	England and Wales	1986–1999 (1986–2001)	201,000+	Affluent	5-years Deprivation gap (%) 1996–1999 −6.0 [−7.4, −4.6]^f, j^
Sloggett et al. (130)	England and Wales	1981–1997 (1981–2000)	1,714	No difference	3-years RER (D:A) 1.07 [0.97–1.18]
Exarchakou et al. (131)	England	1996–2013 (1996–2013)	NS	Affluent	1-year Deprivation gap (%) 2001 −3.7 [−4.3, −3.0] 2006 −2.7 [−3.3, −2.1] 2012 −1.4 [−2.4, −0.3]^f, j^
Rachet et al. (132)	England	1996–2006 (1996–2007)	265,753	Affluent	1-year Deprivation gap (%) 2006 −2.9 (CI not reported)^f, j^
AIHW (133)	Australia	2001–2007 (2006–2010)	NS	Affluent	5-years RS, Figure 6, page 35 article^b^
Stanbury et al. (134)	NSW, Australia	2004–2008 (2004–2008)	NS	Affluent (s)	5-years RER (D:A) 1.72 [1.37–2.15]
Yu et al. (135)	NSW, Australia	1992–2000 (1996–2001)	30,441	No difference (s)	5-years RER (D:A) 1.09 (*p* > 0.05, CI not reported)
Yu et al. (55)	NSW, Australia	1982–2007 (1992–2007)	68,686	Affluent (s, r)	10-years RER (D:A) 1.40 [1.29–1.53]
Jeffreys et al. (136)	New Zealand	1994–2003 (1994–2004)	9,632	Affluent	5-years RS (A) 88% [86–89], (D) 76% [73–78]^b^
**Prostate-cancer specific survival**					
Du et al. (137)	USA	1992–1999 (1992–2002)	61,228	Affluent, local/regional (s, t, c)	HR (D:A) 1.40 [1.20–1.64]
Kish et al. (59)	USA	2002–2008 (2004–2008)	357,078	Affluent	5-years PCS (A) 95.7%, (D) 90.9%^h^ (CI not reported)^b^
Singh and Jemal (60)	USA	1998–1999 (1998–1999)	NS	Affluent	HR (D:A) 1.57 [1.46–1.68]
Ellis et al. (138)	CA, USA	2000–2013 (2000–2013)	270,101	Affluent (s, t)	Figure 1, page 29 article
Hellenthal et al. (139)	CA, USA	1996–2005 (1996–2005)	123,953	Affluent, localized, stratified by treatment	HR (D:A) RP 2.20 [1.38–3.50], EBRT 2.21 [1.66–2.95]
Freeman et al. (140)	Chicago, USA	1986–1990 (1986–2006)	833	Affluent (s, t, c)	HR (D:A) 2.37 [1.76–3.18]
Schwartz et al. (141)	Detroit, USA	1988–1992 (1988–2008)	8,679	Affluent (localized) No difference (regional) (t)	HR (D:A): localized 1.39 [1.14–1.69], regional 1.37 [0.91–2.04]
Niu et al. (93)	NJ, USA	1993–1999 (1993–2004)	41,999	Affluent (s)	5-years HR (D:A) 1.39 [1.24–1.56]
White et al. (110)	TX, USA	1995–2002 (1995–2003)	87,444	Affluent (s, r)	HR (D:A) 1.36 [1.25–1.49]
Miki et al. (70)	Japan	1990–2009 (1990–2011)	732	No difference	HR (D:A) 1.22 [0.75–2.00]^g^
Louwman et al. (142)	South-east Netherlands	1997–2006 (1997–2006)	9,987	Affluent (c)	1-year HR (D:A) 1.36 [1.10–1.70]
Li et al. (111)	Sweden	1990–2008 (1990–2008)	73,159	Affluent (c, r)	OR (D:A) 1.19 [1.10–1.29]
Campbell et al. (112)	Scotland	1991–1995 (1991–1995)	6,833	Affluent	1-year PCS (A) 71.9% (D) 80.1% (CI not reported)^b, j^
Tervonen et al. (113)	NSW, Australia	1980–2008 (1980–2008)	95,543	Affluent (s, r)	SHR (D:A) 1.16 [1.11–1.22]
Tervonen et al. (102)	NSW, Australia	2000–2008 (2000–2008)	47,401	Affluent (s, r)	SHR (D:A) 1.31 [1.16–1.47]
Thomas et al. (114)	QLD, Australia	2005–July 2007 (2005–2011)	7,393	No difference (s, t, r)	5-years PCS SHR (D:A) 1.35 [0.95–1.92]
Papa et al. (85)	Victoria, Australia	1995–2000 (1995–2008)	1,984	No difference, men who had RP (s, r)	10-years PCS SHR (D:A) 1.11 [0.55–2.22]^g^
Haynes et al. (87)	New Zealand	1994–2004 (1994–2004)	25,078	No difference (s)	OR (D:A) 0.98 (*p* > 0.05, CI not reported)

*CA, California; CI, confidence interval; FL, Florida; EBRT, external beam radiotherapy; HR, hazard ratio; NJ, New Jersey; NCCN, National Comprehensive Cancer Network, NS, not stated; NSW, New South Wales; OR, Odds ratio; OS, overall survival; Qld, Queensland; PC, prostate cancer; PCS, prostate cancer survival; RoI, Republic of Ireland; RER, relative excess risk; RP, radical prostatectomy; RS, relative survival; SHR, sub-hazard ratio; TX, Texas; WA, Western Australia*.

a If both crude survival analysis and multivariate analysis conducted, only key findings from latter fully adjusted models shown.

b Crude survival estimates only.

c National Comprehensive Cancer Network (NCCN) prostate cancer risk groups: very low risk (clinical (c) stage T1c, Gleason score [GS] ≤ 6; PSA < 10 ng/l, < 3 positive prostate biopsy cores, ≤ 50% core involved with cancer, PSA density < 0.15 ng/mL/g), low risk (cT1-cT2a; GS ≤ 6; PSA < 10 ng/mL); intermediate (cT2b-cT2c, GS 7, PSA 10–20 ng/mL); high (cT3a, GS ≥8, PSA>20 ng/mL), very high risk (c T3b-cT4, primary GS pattern 5 or >4 biopsy cores with GS 8–10), regional (cN1, clinically positive regional lymph nodes) and metastatic (cM1, clinically positive for metastasis).

d All models adjusted for age.

(s) Also adjusted for some measure of spread of diagnosis, such as stage at diagnosis, Gleason score or National Comprehensive Cancer Network prostate cancer risk groups.

(t) Also adjusted for treatment-related factors.

(d) Also adjusted for area-disadvantage.

(c) Also adjusted for comorbidities.

(r) Also adjusted for urban/rural residence.

e Higher risk of death implies poorer survival whereas lower risk of death implies better survival.

f Deprivation gap is the absolute difference (%) between predicted relative survival estimates for most affluent and most disadvantaged groups from regression models. By convention, a negative value implies lower survival for the most disadvantaged group.

g If the reference category was rural or disadvantaged, the inverse odds ratio or rate ratio was calculated to ensure consistency across Tables and make them easier to read.

h Localized disease is Stage I and II (confined to the prostate).

i Generated from reported coefficients.

j *Significant (p < 0.05)*.

However, all four moderate quality studies, one from Denmark (47) and three from Australia (31, 55, 109) that reported poorer prostate cancer relative survival (excess mortality risk of 1.14–2.53, rural vs. urban) for rural residents did not consider comorbidities or treatment, although two did adjust for stage at diagnosis (55, 109). Four (high quality) studies, two Australian (85, 113), and one each from Scotland (112) and Sweden (111) also found consistently lower prostate cancer survival among rural residents. Only one of these studies adjusted for comorbidities (111), two for stage (85, 113) and none for treatment. However, three other studies (one high, two moderate quality), from Australia (114, 115) and the USA (110) found no significant associations with rurality for stage-adjusted estimates. Of the 11 papers in total that reported net survival (relative or prostate-cancer specific survival), only three found no geographical differentials in survival and eight higher survival among urban men (Figure 2; Table 5).

Finally, most of the 17 included papers focused on medium term survival with two reporting survival 10-years after diagnosis (55, 85).

##### Residential disadvantage

A consistent pattern of poorer overall survival among men diagnosed with prostate cancer while living in disadvantaged areas of the USA was evident across eight (seven high, one moderate quality) (103–106, 116–119) studies (Figure 3; Table 5). Four more studies (three high, one moderate quality), one each from Ireland (120), England (107), Australia (108), and the Netherlands (98) (only among men aged 60–74 years with localized or 60+ years with advanced disease) also reported similar patterns even after adjustment for various combinations of potential explanatory factors, notably stage, treatment and comorbidities (98, 103–106, 108, 116–120). Only one USA-based (high quality) (89) study found no differences by residential disadvantage.

Fifteen studies (seven high, eight moderate quality) reported lower prostate cancer relative survival rates for residents of disadvantaged areas even after adjusting for stage (55, 127, 134) although none controlled for treatment or comorbidities. Six studies were from the UK (22, 126, 127, 129, 131, 132), three from Australia (55, 133, 134) with one each from Colombia (122), France (124), Germany (125), Japan (123), New Zealand (136), and the USA (121). Although the gap in 1-year relative survival for men with prostate cancer from most and least disadvantaged areas in England had narrowed between 1996 and 2013, significant socioeconomic inequalities remained (131, 132). However, two other high quality UK-based studies (128, 130) and one Australian study (moderate quality) (135) found no evidence for differentials in relative survival by residential disadvantage.

Finally, 14 (nine high, five moderate quality) of 18 included studies consistently reported poorer prostate-cancer specific survival among males from disadvantaged areas in the USA (nine studies) (59, 60, 93, 110, 137–141), Australia (two studies) (102, 113), Netherlands (142), Sweden, (111), and Scotland (112) after controlling for diverse explanatory factors. On multivariate analyses, estimated hazard ratios (HR) for increased risk of mortality (poorer survival) ranged from 1.16 to 2.37 (disadvantaged vs. affluent). Three of the four remaining studies (two high, two moderate quality) found no evidence for survival differentials by residential disadvantage in Japan (70), New Zealand (87) and Australia (85) while another Australian study reported that although residential disadvantage was not significantly associated with prostate cancer mortality, male residents of those areas had poorer non prostate-cancer specific mortality (114).

In summary, of 49 included studies, male residents of disadvantaged areas had consistently poorer overall (12 of 13) and net survival (29 of 36) (Figure 3; Table 5) when diagnosed with prostate cancer.

Most studies focused on medium-term survival, with only two (55, 85) following men for longer than 10 years after their cancer diagnosis. Four presented 1-year survival estimates (112, 131, 132, 142).

#### Access and Use of Services

Treatment of early stage prostate cancer, localized disease or National Comprehensive Cancer Network (NCCN) low to intermediate-risk groups remains controversial with no consensus regarding their optimum management (187, 188). Several treatment types are available depending on clinical features, patient age and preferences. For example, men diagnosed with localized (defined as no identifiable regional lymph nodes or distant metastases) disease have three main options: expectant management (EM) that is monitoring for cancer progression while not having curative therapy, curative surgery typically radical prostatectomy (RP) or radiotherapy (RT), such as brachytherapy (BB) or external beam radiotherapy (EBRT). As such, patterns described below for different treatment types do not necessarily imply adverse outcomes by residential location.

For ease of interpretation we have presented patterns below by different treatment types (Figures 4, 5; Table 6) after an overall summary. No studies reporting geographical variations in use of services for metastatic disease were found.

**Table 6:** Summary of included studies on differentials in access and use of services.

**References**	**Location**	**Period**	**Sample size^**a**^**	**Key outcome**	**Findings [95% confidence interval in brackets]^a, b, c, d, e^**
**URBAN/RURAL DIFFERENTIALS (U, URBAN; R, RURAL)**					
**Higher expectant management (monitoring with no definitive therapy)**					
Parikh et al. (143)	USA	2010–2013	40,839	Closest to treating center for very low-risk PC	OR (high: low distance) 0.94 [0.92–0.95]
Schymura et al. (144)	USA	1997	3,328	No difference for localized PC	17.6% (R), 19.2% (U) (*p* > 0.05)^g^
**Higher surgery or radical prostatectomy (RP)**					
Baldwin et al. (77)	USA	2004–2006	48,121	Urban for early stage PC	71.7% (R), 75.8% (U)^g, h^
Schymura et al. (144)	USA	1997	3,328	No difference for localized PC	36.0% (R), 39.9% (U) (*p* > 0.05)^g^
des Bordes et al. (145)	TX, USA	2004–2009	46,971	No difference for localized PC	OR (R:U) 0.94 [0.75–1.19]
Ghali et al. (44)	NH, USA	2004–2011	4,731	Urban for localized PC	37.2% (R), 62.8% (U)^g, h^
Jones et al. (146)	Northern England	1994–2002	20,688	No difference	OR (furthest: closest RT) 0.93 [0.85–1.01]
Baade et al. (31)	Australia	1995–2008	NS	Urban	2007–2008 ASR (R) 182.2, (U) 239.2 (CI not reported)^i^
Coory and Baade (32)	Australia	1995–2002	NS	Urban	2001–2002 Rate ratio (R:U) 0.71 [0.65–0.77]^i^
Hayen et al. (147)	NSW, Australia	1993–2002	33,200	Urban	RR (R:U) 0.69 [0.65–0.73]
Baade et al. (148)	QLD, Australia	2005–2007	1,064	Closest to RT facilities	APP (furthest RT) 35.0 [28–42], (closest RT) 50.2 [47–53]
Hall et al. (108)	WA, Australia	1982–2001	14,123	No difference	HR (R:U) 0.54 [0.29–1.03]
Tan et al. (115)	Victoria, Australia	2004–2014	14,686	Urban (4 age groups: 35–44 to 65–74)	21.1–25.5% (R), 73.7–78.9% (U)^g, h^
**Higher radiotherapy (RT includes BB, EBRT, IMRT)**					
Baldwin et al. (77)	USA	2004–2006	48,121	Rural (BB) for early stage PC	28.3% (R), 24.2% (U)^g, h^
Cobran et al. (149)	USA	2002–2006	10,975	Urban (IMRT) for localized PC	OR (R:U) 0.17 [0.08–0.37]
Schymura et al. (144)	USA	1997	3,328	No difference for localized PC	34.1% (R), 31.1% (U) (*p* > 0.05)^g^
des Bordes et al. (145)	TX, USA	2004–2009	46,971	No difference for localized PC	OR (R:U) 1.16 [0.94–1.43]
Ghali et al. (44)	NH, USA	2004–2011	4,731	Urban (BB) for localized PC	43.8% (R), 56.3% (U)^g, h^
Jones et al. (146)	Northern England	1994–2002	20,688	Closest to RT facilities	OR (furthest: closest RT) 0.88 [0.79–0.99]
Henry et al. (150)	Victoria, Australia	2009	NS	Urban	15.7% (R), 25.8 (U)^g, h^
**Type of curative treatment**					
Cary et al. (151)	USA	2005–2008	138,226	No difference (RT vs. RP) for localized PC	OR (R:U) 1.02 [0.87–1.20]^f^
Cetnar et al. (152)	WI, USA	2004	1,096	No difference (RT vs. RP) for localized PC	OR (R:U) 0.98 [0.53–1.82]
Park et al. (153)	South Korea	2003–2013	1,382	No difference (RT vs. RP)	OR (R:U) 0.77 [0.51–1.17]
Ghali et al. (44)	NH, USA	2004–2011	4,731	Urban (higher RP or BB vs. EBRT) for localized PC	OR (R:U) 0.50 [0.36–0.69]
Glaser et al. (116)	USA	2004–2013	113,719	No difference (EBRT plus BB vs. EBRT) for intermediate or high-risk PC	OR (R:U): intermediate 0.94 [0.81–1.10], high 0.90 [0.75–1.09]
Baldwin et al. (77)	USA	2004–2006	48,121	No difference (RP or BB vs. EBRT) among curatively treated men for early stage PC	73.6% (R), 73.8% (U) (*p* > 0.05)^g^
Williams et al. (154)	USA	2001–2005	10,928	Urban more likely to receive cryotherapy for localized PC	11.1% (R), 88.9% (U)^g, h^
**Higher any curative treatment (RP, RT, combined therapies)**					
Baldwin et al. (77)	USA	2004–2006	48,121	Urban for early stage PC	HR (R:U) 0.75 [0.68–0.83]^f^
Cary et al. (151)	USA	2005–2008	138,226	Urban for localized PC	OR (R:U) 0.81 [0.69–0.95]^f^
Mahal et al. (155)	USA	2004–2010	39,779	Rural for very low-risk PC	OR (R:U) 1.08 [1.01–1.16]^f^
Cetnar et al. (152)	WI, USA	2004	1,096	No difference for localized PC	OR (R:U) 0.96 [0.52–1.77]
Ruseckaite et al. (86)	Victoria, Australia	2008–2013	7,204	Urban	OR (R:U) 0.53 [0.38–0.74]^f^
**Higher hormonal therapy (ADT, ORCH)**					
Hayen et al. (147)	NSW, Australia	1993–2002	33,200	Rural (ORCH)	RR (R:U) 1.36 [1.26–1.47]
Schymura et al. (144)	USA	1997	3,328	No difference for localized PC	12.3% (R), 9.8% (U) (*p* > 0.05)^g^
Skolarus et al. (78)	USA	2008	9,862	No difference in post-EBRT HT for high-risk PC	90.3% (R), 85.3% (U) (*p* > 0.05)^g^
Park et al. (153)	South Korea	2003–2013	1,382	No difference (ADT vs. surgery)	OR (R:U) 1.23 [0.90–1.67]
**Higher quality of care**					
Papa et al. (85)	Victoria, Australia	1995–2000	1,984	Urban-private hospitals or high volume surgical care	OR (private, R:U) 0.43 [0.34–0.56] High volume surgeons: 51% (R), 72% (U)^g, h^
Mahal et al. (156)	USA	2004–2012	138,019	Urban-academic centers for high-risk PC	Academic centers: 1.4% (R), 84.6% (U)^g, h^
Skolarus et al. (78)	USA	2008	9,862	Urban-comprehensive cancer facilities	46.0% (R), 63.0% (U)^g, h^
Skolarus et al. (78)	USA	2008	9,862	No difference in timeliness of care	97 days (R), 106 days (U) (*p* > 0.05)^g^
Skolarus et al. (78)	USA	2008	9,862	No difference in 2 of 3 quality of care measures	10 cores at biopsy 88.7% (R), 90.1% (U) (*p* > 0.05)^g^ CT (metastatic PC) 41.2% (R), 53.1% (U) (*p* > 0.05)^g^ Recommended RT dosage 63.3% (R), 74.5% (U)^g, h^
**Access to care**					
Skolarus et al. (78)	USA	2008	9,862	Rural poorer access to treatment centers	Median distance (miles) 15 (U), 82 (R)^g, h^
Vetterlein et al. (106)	USA	2004–2012	775,999	Rural poorer access to treatment centers	OR (R:U) 119 [107–134]
Aggarwal et al. (157)	England	2010–2014	44,363	Rural more likely to travel to larger more comprehensive RT facilities	More than 1 h OR (R:U) 1.87 [1.51–2.33]
Aggarwal et al. (158)	England	2010–2014	19,256	Rural more likely to travel to established robotic centers and those with high reputation for RP	More than 1 h OR (R:U) 2.14 [1.84–2.47]
Sharma et al. (159)	QLD, Australia	2010–2013	587	Access to RT facility impacts RT uptake among rural men	Figures in article^g^
**RESIDENTIAL AREA DISADVANTAGE DIFFERENTIALS (A, AFFLUENT; D, DISADVANTAGED)**					
**Higher expectant management (monitoring with no definitive therapy)**					
Krishna et al. (160)	USA	2004–2009	2,916	Affluent (AS vs. WW)^h^ for low-risk PC	OR (D:A) 0.47 [0.34–0.65]^f^
Aarts et al. (98)	South-east Netherlands	1998–2008	11,086	Disadvantaged (age < 60) for localized PC	OR (D:A): Age < 60 1.64 [1.01–2.64], Age 60–74 1.20 [0.99–1.45]
McVey et al. (100)	UK	2000–2006	43,222	Disadvantaged	23.0% (A), 36.0% (D)^g, h^
**Higher surgery or radical prostatectomy (RP)**					
Hoffman et al. (161)	USA	2003–2008	17,206	No difference for localized PC	OR (D:A) 1.04 [0.88–1.25]^f^
Krupski et al. (162)	USA	1995–1999	96,769	Affluent for non-metastatic PC	OR (D:A) 0.79 [0.74–0.95]
Marsh et al. (104)	USA	2004–2012	151,663	Affluent for intermediate-risk PC	38% (A), 14% (D)^g, h^
Schymura et al. (144)	USA	1997	3,328	Affluent for localized PC	41.2% (A), 35.8% (D)^g, h^
des Bordes et al. (145)	TX, USA	2004–2009	46,971	Affluent for localized PC	OR (D:A) 0.65 [0.60–0.69]
Aarts et al. (98)	South-east Netherlands	1998–2008	11,086	Affluent for localized PC	OR (D:A): Age < 60: 0.57 [0.40–0.81], Age 60–74 0.75 [0.62–0.91]
Lyratzopoulos et al. (99)	UK	1995–2006	35,171	Affluent	OR (D:A) 0.59 [0.46–0.76]
McVey et al. (100)	UK	2000–2006	43,222	Affluent	34.0% (A), 19.0% (D)^g, h^
Fairley et al. (163)	Northern England	2000–2006	21,334	Affluent for non-metastatic PC	OR (D:A) 0.64 [0.55–0.75]
Jones et al. (146)	Northern England	1994–2002	20,688	Affluent	OR (unit increase disadvantage) 0.99 [0.98–0.99]
Hall et al. (108)	WA, Australia	1982–2001	14,123	Affluent	HR (D:A) 0.63 [0.47–0.83]
Hayen et al. (147)	NSW, Australia	1993–2002	33,200	Affluent	RR (D:A) 0.83 [0.78–0.89]
**Higher radiotherapy (RT includes BB, EBRT, IMRT)**					
Cobran et al. (149)	USA	2002–2006	10,975	Affluent (IMRT) for localized PC	OR (D:A) 0.44 [0.36–0.53]^f^
Hoffman et al. (161)	USA	2003–2008	17,206	No difference for localized PC	OR (D:A) 1.03 [0.93–1.14]^f^
Krupski et al. (162)	USA	1995–1999	96,769	Affluent for non-metastatic PC	OR (D:A) 0.74 [0.69–0.79]
des Bordes et al. (145)	TX, USA	2004–2009	46,971	Affluent for localized PC	OR (D:A) 0.85 [0.79–0.90]
Krupski et al. (164)	USA	1995–1999	34,763	No difference adjuvant RT for non-metastatic PC	OR (D:A) 0.92 [0.75–1.11]
Jin et al. (165)	Canada	2012	2,663	No difference adjuvant RT 6 months post-RP for high-risk PC	OR (D:A) 1.11 [0.60–2.08]^f^
Fairley et al. (163)	Northern England	2000–2006	21,334	Affluent (BB or EBRT) for non-metastatic PC	OR (D:A): BB 0.32 [0.22–0.47], EBRT 0.83 [0.74–0.94]
Jones et al. (146)	Northern England	1994–2002	20,688	Affluent	OR (unit increase disadvantage) 0.99 [0.98–0.99]
Lyratzopoulos et al. (99)	UK	1995–2006	35,171	Affluent	OR (D:A) 0.71 [0.63–0.81]
**Type of curative treatment**					
Glaser et al. (116)	USA	2004–2013	113,719	Affluent (EBRT plus BB vs. EBRT) for intermediate or high-risk PC	OR (D:A): intermediate 0.82 [0.78–0.87]^f^, high 0.72 [0.67–0.78]^f^
Muralidhar et al. (166)	USA	2004–2012	222,084	Disadvantaged (RT vs. RP) for localized PC (by urban/rural areas)	OR (D:A): urban 1.20 [1.15–1.25]^f^, rural 2.08 [1.33–3.23]^f^
Watson et al. (167)	PA, USA	2012–2014	2,194	No difference (RT vs. RP) for localized PC	OR (D:A) 1.27 [0.82–1.96]^f^
Aarts et al. (98)	South-east Netherlands	1998–2008	11,086	Affluent (one-time BB, age 60–74) for localized PC Disadvantaged (daily EBRT, age < 60) for localized PC	OR (D:A): Age < 60 1.04 [0.65–1.66], Age 60–74 0.62 [0.50–0.79] OR (D:A): Age < 60 1.78 [1.10–1.87], Age 60–74 1.14 [0.96–1.34]
Williams et al. (154)	USA	2001–2005	10,928	Disadvantaged more likely to receive cryotherapy than BB for localized PC	42.0% (D), 12.8% (A)^g, h^
**Higher any curative treatment (RP, RT, combined therapies)**					
Baldwin et al. (77)	USA	2004–2006	48,121	Affluent for early stage PC	HR (D:A): 0.75 [0.64–0.88]
Byers et al. (89)	USA	1997	4,332	Affluent	78% (A), 67% (D)^g, h^
Gilbert et al. (168)	USA	1991–2005	28,053	Affluent for low-risk PC	OR (D:A) 0.87 [0.80–0.95]
Mahal et al. (155)	USA	2004–2010	39,779	Affluent for very low-risk PC	OR (D:A) 0.80 [0.76–0.85]^f^
Watson et al. (167)	PA, USA	2012–2014	2,194	Affluent for localized PC	OR (D:A) 0.64 [0.41–0.99]^f^
Fairley et al. (163)	Northern England	2000–2006	21,334	Affluent for non-metastatic PC	OR (D:A): 0.60 [0.54–0.68]
**Higher hormonal therapy (ADT, ORCH)**					
Byers et al. (89)	USA	1997	4,332	Disadvantaged	6% (A), 12% (D)^g, h^
Gilbert et al. (168)	USA	1991–2005	28,053	Disadvantaged for low-risk PC	OR (D:A) 1.15 [1.03–1.29]
Hoffman et al. (161)	USA	2003–2008	17,206	Disadvantaged (ADT) for localized PC	OR (D:A) 1.19 [1.03–1.37]^f^
Aarts et al. (98)	South-east Netherlands	1998–2008	11,086	No difference for localized PC	OR (D:A): Age < 60 1.57 [0.97–2.54], Age 60–74 1.06 [0.89–1.27]
Hayen et al. (147)	NSW, Australia	1993–2002	33,200	Disadvantaged (ORCH)	RR (D:A) 1.30 [1.15–1.46]
Krupski et al. (169)	USA	1991–1999	65,716	No difference in secondary therapy for non-metastatic PC	OR (unit increase income): post-RP 0.99 [0.99–1.00] OR (unit increase income): post-RT 1.00 [0.99–1.01]
Fairley et al. (163)	Northern England	2000–2006	21,334	Disadvantaged for non-metastatic PC	OR (D:A): 1.56 [1.42–1.71]
**Access to care**					
Jin et al. (165)	Canada	2012	2,663	No difference in rates of referral to radiation oncologists within 6 months post-RP for high-risk PC	OR (D:A) 0.89 [0.6–1.32]^f^
Aggarwal et al. (157)	England	2010–2014	44,363	Affluent more willing to travel to larger more comprehensive RT facilities	More than 1 h OR (D:A) 0.76 [0.62–0.95]^f^
Aggarwal et al. (158)	England	2010–2014	19,256	Affluent more willing to travel to robotic surgical centers and those with high reputation for RP	31–60 min OR (D:A) 0.74 [0.52–0.87]^f^

APP, adjusted predicted probabilities; ADT, androgen deprivation therapy; AS, active surveillance; BB, brachytherapy; CA, California; CI, confidence interval; EBRT, external beam radiotherapy; HR, hazard ratio; HT, hormone therapy; IMRT, intensity-modulated radiotherapy; NH, New Hampshire; NS, not stated; NSW, New South Wales; OR, Odds ratio; ORCH, orchiectomy, Qld, Queensland; PA, Pennsylvania; PC, prostate cancer; RP, radical prostatectomy; RR, relative risk; RT, radiotherapy; TX, Texas; UK, United Kingdom; USA, United States; WA, Western Australia; WI, Wisconsin; WW, watchful waiting.

a Findings based on model-based estimates adjusted at least for age and geographical measure, except where indicated.

b Expectant management consists of active surveillance and watchful waiting. Watchful waiting involves monitoring with intent of treating symptoms with palliative intent, whereas active surveillance for very low-risk prostate cancer involves actively monitoring cancer progression, with an intent to provide curative therapy if cancer progresses.

c Definitive treatment includes radical prostatectomy, radiotherapy (brachytherapy, external beam radiotherapy) or a combination thereof.

d Localized disease has no identifiable regional lymph nodes or distant metastases (stage I and II or stage cT1-cT2). Early-stage disease (stage T1-T2NOS; Gleason score < 8; PSA ≤ 20 ng/mL).

e National Comprehensive Cancer Network (NCCN) prostate cancer risk groups: very low risk (clinical (c) stage T1c, Gleason score [GS] ≤ 6; PSA < 10 ng/l, < 3 positive prostate biopsy cores, ≤ 50% core involved with cancer, PSA density < 0.15 ng/mL/g), low risk (cT1-cT2a; GS ≤ 6; PSA < 10 ng/mL); intermediate (cT2b-cT2c, GS 7, PSA 10–20 ng/mL); high (cT3a, GS ≥8, PSA>20 ng/mL), very high risk (c T3b-cT4, primary GS pattern 5 or >4 biopsy cores with GS 8–10), regional (cN1, clinically positive regional lymph nodes) and metastatic (cM1, clinically positive for metastasis).

f If the reference category was rural or disadvantaged, the inverse odds ratio or rate ratio was calculated to ensure consistency across Tables and make them easier to read.

g Findings based on descriptive statistics.

h Significant (p < 0.05).

i *Findings based on age-standardized rates (per 100,000 men)*.

##### Rurality

Twenty-two (11 high, 11 moderate) out of 28 included studies reported geographical variations in access and use of services among men diagnosed with prostate cancer with six (four high, two moderate quality) finding no differences.

###### Expectant management.

While men living furthest away from treating facilities were 8% less likely to have expectant management for very low-risk prostate cancer in the USA from 2010 to 2013 (143), an earlier study reported no differences by residential rurality for localized disease (144).

###### Radical prostatectomy.

Patterns for RP varied with two USA-based (44, 77) and five Australian studies (31, 32, 115, 147, 148) reporting higher rates among urban men or those living closer to major treatment facilities (148). Whereas, two other studies from the USA (144, 145) as well as one each from Australia (108) and England (146) reported no geographical differentials.

###### Radiotherapy.

Included studies gave mixed results for differentials in RT rates for localized or early stage prostate cancer in the USA, with two finding no significant differences (144, 145), one higher rates of BB among rural (77) and one among urban residents (44). Rural men were also 83% less likely to receive intensity-modulated RT (IMRT) for localized prostate cancer (149). Increasing distance from radiation centers in England was associated with lower RT among men diagnosed with prostate cancer (146), while in Australia RT rates were higher among urban than rural males (150).

###### Type of curative treatment.

A study from South Korea (153) and two from the USA (151, 152) found no association between residential rurality and the type of curative treatment received (i.e., RP vs. RT). Moreover, among men with early stage prostate cancer who underwent curative treatment in the USA, urban and rural residents were about equally as likely to receive a one-time treatment (RP or BB) (77), whereas urban residence was associated with greater use of one-time RP or BB rather than daily EBRT for localized disease in another study (44). However, there was no difference by residential location in the use of combined radiotherapies (EBRT and BB vs. EBRT) for intermediate or high-risk disease (116). Urban men were more likely to undergo cryotherapy for localized disease in a single USA-based study (154).

###### Any curative treatment.

Findings from the USA indicated that rural men were 19 to 25% less likely to receive any curative treatment for localized (151) or early-stage (77) disease, whereas a single state-based study found no geographical differences in receipt of curative treatments for localized disease in Wisconsin, USA (152). However, rural residents were more likely to undergo treatment than active surveillance for very low-risk disease (155). Finally, urban men were around two times more likely to undergo curative treatment in one Australian study (86).

###### Hormone therapy.

A consistent pattern of no geographical differentials in hormone therapy (HT) was reported by two USA-based (78, 144) and one South Korean study (153), although in Australia rural men were 36% more likely to undergo orchiectomy (147).

###### Quality of care.

Although one USA-based study found that urban men were more likely to be treated at comprehensive care facilities, no geographical differentials were evident in the timeliness or quality of their care except for receipt of recommended RT dosage (78). Urban residents were more likely to be treated for high-risk disease at academic centers in the USA (156) and by high-volume surgeons or private hospitals in Australia (85).

###### Access to care.

Two USA-based studies reported poorer access to treatment centers among rural men (78, 106), while in Australia, improving access to RT facilities increased its uptake among rural prostate cancer patients (159). Finally, rural residents were more likely than their urban counterparts to undergo prostate cancer-related treatment at larger more comprehensive RT facilities (157) or established centers for robotic surgery rather than nearest facilities (based on travel times) in England (158).

##### Residential disadvantage

A clear and persistent pattern of variations in the access and use of prostate cancer related services by residential disadvantage was evident across 24 (11 high, 13 moderate quality) of 27 included studies with three (high quality) reporting no difference.

###### Expectant management.

One USA-based study found that residents of affluent areas (vs. disadvantaged) with low-risk disease were two times more likely to be under active surveillance (a strategy of close monitoring, with intent of curative treatment on disease progression) than watchful waiting (monitoring and treating symptoms with palliative intent) (160). By contrast one study from the UK (100) and one from the Netherlands (98) reported that men aged below 60 years from disadvantaged areas were more likely to be under expectant management for low-risk or localized disease, respectively.

###### Radical prostatectomy.

Four studies from the USA reported that residents of affluent areas had higher rates of RP for localized (144, 145), intermediate-risk (104) and non-metastatic prostate cancer (162), while one found no differences (161). Men from disadvantaged areas were consistently less likely (range 17–44% vs. affluent) to undergo RP in the UK (four studies) (99, 100, 146, 163), Australia (two studies) (108, 147) and the Netherlands (98).

###### Radiotherapy.

Higher residential advantage was consistently associated with greater RT usage (OR 0.32–0.85 disadvantaged vs. affluent) in the USA (145, 162) and UK (99, 146, 163) with one USA-based study finding no differences (161). Males from affluent areas in the USA were more likely to receive IMRT for localized disease (149). However, rates of adjuvant RT after surgery did not vary by residential disadvantage in one USA-based (164) and one Canadian study (165).

###### Type of curative treatment.

While one study found that men from disadvantaged areas were less likely to receive RT than surgery for localized prostate cancer in the USA (166), another found no significant differentials by residential disadvantage (167). However, affluent residents had higher usage of combined EBRT and BB (vs. EBRT) for intermediate or high-risk disease (116) and were more likely to undergo BB than cryotherapy for localized disease (154). In the Netherlands, men aged 60–74 from affluent areas were significantly more likely to have one-time BB, whereas higher receipt of EBRT was associated with living in disadvantaged areas, for those aged below 60 years (98).

###### Any curative treatment.

A consistent pattern of men living in disadvantaged areas being 13 to 40% less likely to receive curative treatment when diagnosed with prostate cancer of varying stage or risk group was evident across five studies from the USA (77, 89, 155, 167, 168) and one from England (163).

###### Hormone therapy.

By contrast, residents of disadvantaged areas had higher rates of HT in three studies from the USA (89, 161, 168) and one each from England (163) and Australia (147) with no differentials found in the Netherlands (98). One USA-based study found no association between residential disadvantage and secondary HT after primary curative treatment for non-metastatic prostate cancer (169).

###### Access to care.

Men from affluent areas were more likely to travel beyond their closest treatment centers to larger established centers in England especially those offering robotic surgery (158) or innovative radiation therapies, such as IMRT or proton beam therapy (157). Finally, there were no differences in post-surgery referral rates to radiation oncologists for high-risk disease between affluent and disadvantaged areas in Ontario, Canada (165).

#### Prostate Cancer Mortality

##### Rurality

Eleven (five high, six moderate quality) of the 18 included studies consistently reported higher prostate cancer mortality rates among rural residents (25, 31, 32, 45, 172–177, 181), one (moderate) the reverse trend (179) and four (one high, three moderate quality) no differences (171, 178, 180, 182) (Figure 2; Table 7). A (high quality) study by Lagace et al. (170) found higher prostate cancer mortality rates among rural men in Canada and a trend toward higher death rates in rural areas for Australia. Another high quality study also reported higher prostate cancer mortality rates outside urban areas in Australia, although the difference between most extreme remote and urban category was not significant (52).

**Table 7:** Summary of included studies on differentials in prostate cancer mortality.

**References**	**Location**	**Period**	**No of deaths**	**Highest mortality**	**Findings [95% confidence interval in brackets]^**a, b**^**
**URBAN/RURAL DIFFERENTIALS (U, URBAN; R, RURAL)**					
Lagace et al. (170)	Canada	1986–1996	NS	Rural	SMR (R:U) 1.09 [1.01–1.18]^c^
Pampalon et al. (171)	Quebec, Canada	1998–1998	NS	No difference	ASR (R) 29.8, (U) 29.4 (*p* >0.05, CI not reported)^c^
Colli and Amling (172)	USA	2000–2003	NS	Rural	HR (1 unit increase in urbanization) 0.95^c, d, e^
Jemal et al. (173)	USA	1970–1989	453,896	Rural	Maps in article^c^
Jemal et al. (25)	USA	1995–2000	NS	Rural	Rate ratio (R:U) 1.04 (W) 1.12 (AA)^c, d^
Odisho et al. (174)	USA	2001–2005	NS	Rural	(% change mortality, U:R) 8.06% lower [−10.94, −5.18]^d^
Rogerson et al. (175)	USA	1968–1998	NS	Rural	Maps in article^c^
Rusiecki et al. (176)	USA	1950–2000	NS	Rural	RR (R:U) 1.03 [1.01–1.06]
Singh et al. (177)	USA	2003–2007	NS	Rural	Rate ratio (R:U) Overall 1.06, W 1.08, AA 1.22^c, d^
Higginbotham et al. (45)	MS, USA	1996	430	Rural	Rate ratio (R:U) 1.15^c, d^
Zahnd et al. (178)	MS, USA	2008–2012	NS	No difference	ASR (R) 25.9 [24.9–26.9], (U) 26.1 [25.1–27.9]^c^
Yang and Hsieh (179)	Taiwan	1982–1991	NS	Urban	Rate ratio (R:U) 0.55 [0.38–0.78]^c, f^
Hagedoorn et al. (180)	Belgium	2001–2011	NS	No difference	ASR (R) 25.5 [48.8–53.3], (U) 30.3 [50.1–54.8] RR (R:U) 1.02 (*p* > 0.05, CI not reported)
Nikolaidis et al. (181)	Greece	1999–2008	NS	Rural	RR (R:U) 1.86 [1.10–3.14]
Smailyte and Kurtinaitis (182)	Lithuania	1993–2004	NS	No difference	2004 ASR (R) 20.4 (U) 20.2 (*p* > 0.05, CI not reported)^c^
AIHW (52)	Australia	2006–2010	NS	No difference	ASR (R) 33.0 [28.9–37.6], (U) 29.1 [28.5–29.7]^c^
Baade et al. (31)	Australia	1985–2007	NS	Rural	2008 Rate ratio (R:U) 1.24 [1.11–1.38]^c^, Box 2, page 295 article
Coory and Baade (32)	Australia	1985–2002	NS	No difference (1985–1987) Rural (1988–2002)	Rate ratio (R:U)^c^: 1985–1987 1.06 [0.97–1.15] 2000–2002 1.21 [1.14–1.29]^c^
Lagace et al. (170)	Australia	1997–1999	NS	No difference	SMR (R:U) 1.02 [0.73–1.39]^c^
**RESIDENTIAL AREA DISADVANTAGE DIFFERENTIALS (A, AFFLUENT; D, DISADVANTAGED)**					
Jemal et al. (173)	USA	1970–1989	453,896	No difference^c^	Maps in article^c^
Odisho et al. (174)	USA	2001–2005	NS	Disadvantaged	(% change mortality, unit increase advantage) 0.38% lower [−0.64, −0.12]^d^
Rand et al. (183)	USA	2004–2011	83	No difference	OR (D:A) 0.56 [0.18–1.75]
Singh et al. (177)	USA	2003–2007	NS	Disadvantaged	Rate ratio (D:A) Overall 1.21, W 1.03, AA 1.26^c, d^
Cheng et al. (62)	CA, USA	1999–2001	8,997	Disadvantaged^c^	Rate ratio (D:A) 1.14 [1.05–1.09]^c, f^
Wan et al. (184)	TX, USA	1996–2004	14,036	Disadvantaged (census block group/tract)^g^ Affluent (county)	OR (D:A): Block group 1.18 [1.02–1.37], tract 1.16 [1.01–1.34] County 0.77 [0.65–0.90]
Zahnd et al. (178)	MS, USA	2008–2012	NS	Disadvantaged	ASR (A) 23.9 [22.7–25.2], (D) 27.0 [26.1–27.9]^c^
Soto-Salgado et al. (68)	Puerto Rico	1992–2004	NS	No difference	RR (D:A) 1.14 [0.98–1.30]^f^
Miki et al. (70)	Japan	1990–2009	422	No difference	HR (D:A) 1.30 [0.58–2.86]^f^
Hagedoorn et al. (180)	Belgium	2001–2011	NS	No difference	ASR (A) 25.0 [48.2–52.5], (D) 31.3 [49.9–54.9], RR (D:A) 0.97 (*p* > 0.05, CI not reported)
Pukkala and Weiderpass (72)	Finland	1971–1995	3,020	No difference	SIR (A) 1.18 [1.06–1.32], (D) 0.93 [0.84–1.02]^c^
Borrell et al. (185)	11 cities, Spain	1996–2003	8,914	No difference	RR range (D:A) 0.64 [0.37–1.04] to 1.12 [0.58–1.89]
Morgan et al. (35)	Scotland	2003–2008	822	No difference	OR (D:A) 1.22 [0.63–2.33]^f^

ASR, age-standardized rate; CA, California; CI, confidence interval; HR, hazard ratio; MS, Mississippi; NS, not stated; OR, Odds ratio; RR, relative risks; SIR, standardized mortality ratio; USA, United States; W, White.

a Findings based on model-based estimates adjusted at least for age and geographical measure, except where indicated.

b Reference citations provided when authors gave only figures.

c Findings based on age-standardized mortality rates (per 100,000 men) and/or standardized mortality ratios.

d Significant (p < 0.05).

e Generated from reported coefficients.

f If the reference category was rural or disadvantaged, the inverse odds ratio or rate ratio was calculated to ensure consistency across Tables and make them easier to read.

g *Standard hierarchy of census geographical entities in USA: counties (primary administrative units of states); census tracts (small subdivisions of a county, 1,200–8,000 people) and census block groups (statistical divisions of census tracts, 600–3,000 people)*.

In the USA, men from rural areas had 3–15% higher prostate cancer mortality rates than those from urban areas across four studies (25, 45, 176, 177) with four more reporting similar patterns (172–175). Although the study by Zhand et al. (178) found no rural urban differences in prostate cancer mortality rates in the Mississippi Delta region, both urban and rural rates were higher compared to corresponding urban and rural areas for other regions in the USA.

##### Residential disadvantage

Five (two high, three moderate quality) (62, 174, 177, 178, 184) of the seven included studies from the USA reported higher prostate cancer mortality rates in disadvantaged areas with two (high quality) (173, 183) reporting no difference (Figure 3; Table 7). By contrast, all six studies (two high, four moderate) from other countries, one each from Puerto Rico (68), Japan (70), Belgium (180), Finland (72), Spain (185), and Scotland (35) reported no difference. A noteworthy point is that the significantly higher prostate cancer mortality rates among disadvantaged men in Texas between 1996 and 2004 reported by Wan et al. (184) only held for the smaller census block groups and tract-level geographical units of the USA-census, with the effect reversing for larger county-level areas.

#### Effects of Rurality After Adjustment for Residential Disadvantage

Of the 169 articles included, 34 (20%) presented estimates of rurality, adjusted for residential disadvantage, for at least one of the considered outcomes. The proportion of studies including these adjusted estimates varied for each outcome, ranging from 11% (2 of 18) for prostate cancer mortality, 18% for PSA testing (2 of 11) and prostate cancer incidence (4 of 23), 31% for spread of disease (5 of 16), 36% for access and use of services (10 of 28), 65% for net survival (7 of 11) and 100% for overall survival (6 of 6).

**Figure 6:**
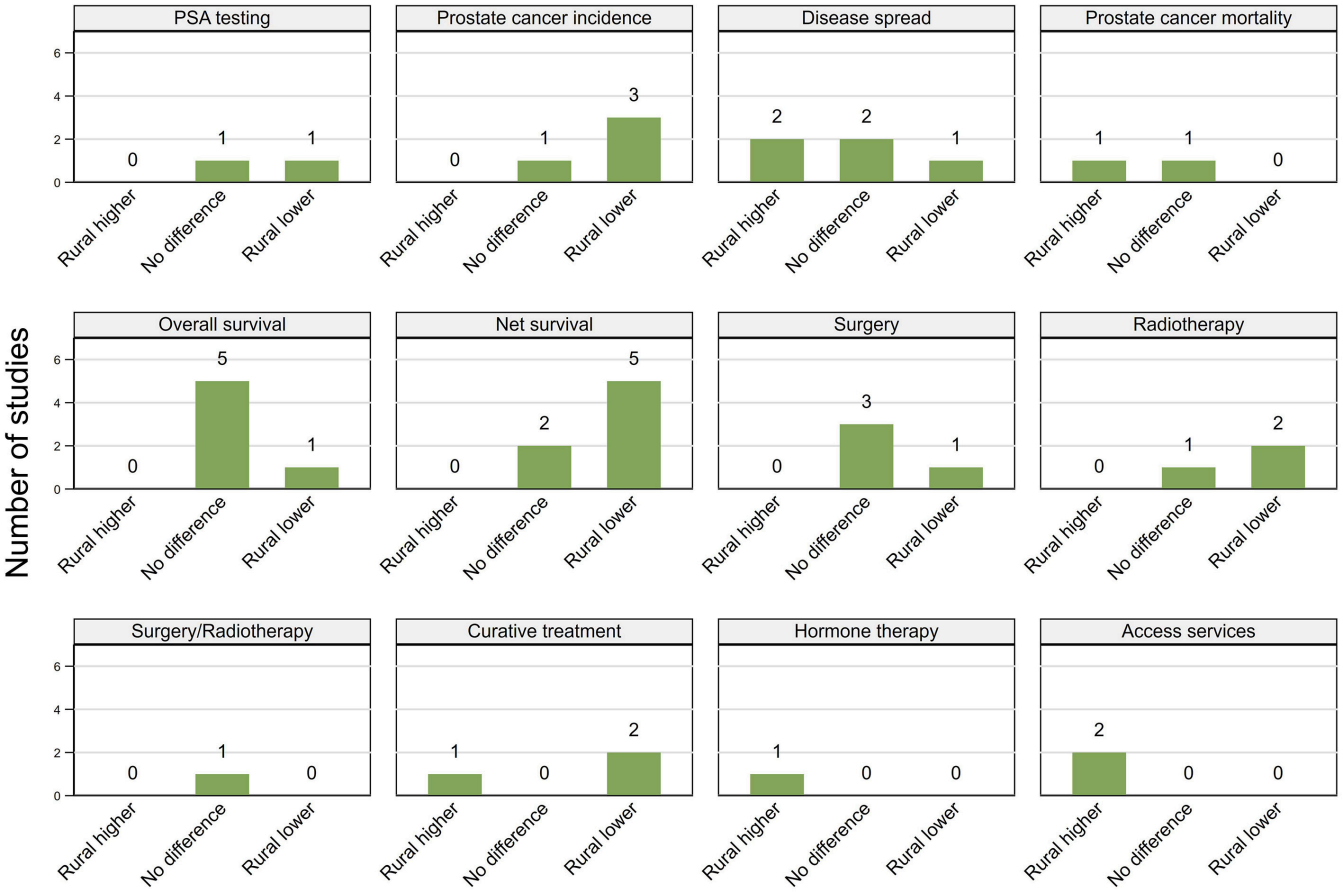
Summary of key patterns by rurality from studies that also adjusted for residential disadvantage.

The majority of the studies including adjusted estimates were from the USA, followed by Australia and Canada (Supplemental File 4). However, there was wide heterogeneity across studies in the covariates included in the statistical models; this does limit comparisons across them. In addition, only two studies presented both unadjusted and adjusted estimates by rurality, hence we cannot reliably assess the effect of rurality after controlling residential disadvantage. Nevertheless, after adjustment for residential disadvantage, there was a consistent pattern for rurality to remain independently associated with geographical variations in prostate cancer incidence, net survival and access to services (Figure 6), and for no independent association with overall survival. There were no consistent patterns for PSA testing, mortality or advanced spread of disease.

**Figure 7:**
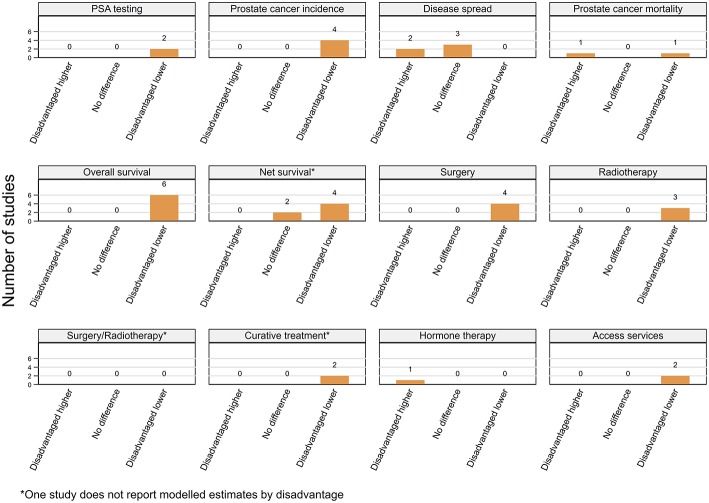
Summary of key patterns by residential disadvantage from studies that also adjusted for rurality.

Overall, for most outcomes except advanced spread of disease and prostate cancer mortality, there appeared to be more consistent evidence of an independent effect for residential disadvantage than for rurality (Figure 7).

In summary, included studies did not provide sufficient evidence to conclude that the urban/rural differential in prostate cancer outcomes was completely accounted for by disparities in residential disadvantage. However, there is a suggestion that residential disadvantage may have a stronger effect on prostate cancer disparities than living in an urban or rural area.

## Discussion

This systematic review found a consistent pattern of differences by residential disadvantage across the prostate cancer continuum from PSA testing to incidence, staging, treatment, survival and mortality. Specifically, compared to residents of affluent areas, men living in socioeconomically disadvantaged areas generally had lower PSA testing and prostate cancer incidence, more advanced spread of disease at diagnosis, poorer survival, and higher mortality. Findings by rurality were less consistent. Where a pattern was observed, it was that men from rural areas had lower PSA testing, incidence and survival, more advanced disease and higher prostate cancer mortality than urban residents. There was also evidence that men from more rural or disadvantaged areas had poorer access and use of prostate cancer-related treatment services than those from urban or advantaged areas.

Although the underlying reasons for these variations are not known, there is widespread consensus that they reflect complex and interacting social, genetic, environmental and behavioral processes that can occur at a range of geographical scales (7, 189). The finding that some observed geographical patterns varied by ethnic status, such as with PSA testing for rurality (26, 27), incidence by area disadvantage (41, 46), while others were consistent, such as incidence by rurality (46) highlights the likely complexity of these processes.

Prostate cancer incidence rates increased sharply with the dissemination of PSA-based testing in the early 1990s (5, 190). It is likely that some of the observed geographical patterns in prostate cancer incidence and survival may reflect geographical variations in PSA testing prevalence. Specifically, men from urban (25, 28–33) and affluent (23, 29, 34, 35) areas had higher PSA testing rates than those from rural or disadvantaged areas, respectively. This has been suggested to be due to differential access to screening services and health care (26, 27, 29, 30), GP attitudes (27, 30, 33, 34), health literacy (26–28, 30), socio-cultural norms (26, 27), and help-seeking behaviors (26–28, 30). There was widespread agreement that the higher prostate cancer incidence (15, 16, 31, 39, 41, 42, 47, 48, 53, 56, 62, 68, 73, 76) and lower advanced stage diagnoses (21, 24, 25, 84, 86, 88) among urban and affluent men reflected increased detection of localized and latent cancers through PSA testing. The impact of PSA testing on prostate cancer mortality risks however remains controversial (2, 191). Variations in potential risk factors and health behaviors reflecting physical and social environment (35, 36, 47–49, 56, 60, 62, 125, 177, 180), health care quality, access or utilization (24, 62, 68, 71, 80, 177, 180) and availability of specialists (41, 79, 174) were other commonly cited reasons for the geographical disparities in prostate cancer incidence, stage and mortality.

Non-clinical factors that have been suggested to contribute to geographical patterns of care include differences in access to and availability of treatment modalities (77, 85, 151, 153, 157, 158), clinician practice patterns (86, 108, 116, 145, 151, 153, 162), patient preferences (77, 86, 98, 108, 151, 153, 155, 162), comorbidities (77, 98, 99, 116, 161, 162), and treatment decision-making processes (98, 99, 161, 162). Variations in treatment could also potentially reflect the managing physician's preferences (99, 161, 192) which, in the absence of a definitive treatment guideline for prostate cancer, strongly influences prostate cancer treatment choices (99, 192, 193).

Although stage at diagnosis impacts prostate cancer survival (60, 89, 125, 127), survival differentials by residential location were evident even after adjustment for stage (55, 85, 93, 102, 110, 113, 127, 134, 137, 138, 140), and in some instances also adjusted for treatment (137, 138, 140). Many of the proposed explanations for the geographical variations in survival were multifactorial and included variations in psychosocial factors (93, 98, 105, 108, 111, 113, 117), comorbidities (98, 104, 113, 125, 126, 135, 139, 142), access to high-quality healthcare (55, 60, 93, 98, 103, 104, 106, 107, 110, 113, 117, 125, 127, 135, 139, 141), intensity of clinical follow-up (55, 102, 127, 139), and compliance with recommended treatments (47, 106, 125). Finally, even after adjusting for stage, there is likely to be a residual confounding by PSA testing, in that the observed survival differentials may reflects the diagnosis of latent prostate cancers through PSA testing rather than a true difference in survival (194).

It is likely that inequalities in access to diagnostic and treatment services is a key factor contributing to the geographical disparities in prostate cancer outcomes. These inequalities are influenced by socioeconomic factors, health care policies and proximity to medical services. For rural residents, a diagnosis of prostate cancer can present unique challenges in obtaining appropriate, high-quality care, including limited local services and long travel distances incurring financial, psychosocial and logistical barriers (195–197). Several of the studies in this review found that increasing travel burden impacted treatment (116, 143, 146, 150, 159, 166). Moreover, high-volume specialists and hospitals which have been associated with rapid adoption of innovative treatments and technologies (149, 158, 196), multidisciplinary care (103, 143, 165) and better prostate cancer-related clinical outcomes (103, 106, 198) are typically concentrated in urban areas (196, 199). The close overlap between rurality and residential disadvantage in countries, such as Australia (85, 195) and the USA (77, 200) and the necessity for repeated visits for specific treatments like EBRT are likely to worsen the impact of accessibility-related barriers.

From the studies that reported results adjusted for both area disadvantage and rurality, there was consistent evidence that the strong impact of residential disadvantage remained even after adjusting for rurality. While its effect was diluted, it appears that the urban/rural differential in prostate cancer outcomes was not completely accounted for by disparities in residential disadvantage. Therefore, living in rural areas constitutes an additional disadvantage in terms of prostate cancer outcomes, over and above residential socioeconomic disadvantage itself. As such, rather than considering these two geographical measures separately, both rurality and socioeconomic disadvantage need to be considered together in terms of their impact on inequalities in burden of prostate cancer.

### Findings in Context of Other Studies

We are not aware of any previous systematic reviews reporting the international evidence for variations along the continuum of prostate cancer outcomes by rurality and residential disadvantage. Our findings were consistent with two earlier systematic reviews on variations in prostate cancer incidence and mortality by rurality (5) and survival by socioeconomic disadvantage (7), respectively. Two earlier reviews also found persistent geographical disparities across a range of prostate cancer outcomes in the USA (201) and worldwide (4). None of these previous reviews critically assessed studies or included the one-third (58 of 169) of articles included in this review published since 2014.

### Key Gaps in Current Literature and Suggestions for Future Research

We found only six studies looking at geographical variations in expectant management (EM) for low-risk disease. Differences in use of EM, and the lack of standardized definitions and/or protocols during the time-periods of the included studies impaired the comparability across studies. Only one study (160) specifically distinguished between two main EM strategies of watchful waiting (palliative treatment for symptomatic progression) and active surveillance (curative treatment on evidence of disease progression).

This review highlighted the need for large, high quality studies that include the whole range of prostate cancer indicators within the one cohort. It is likely this will require an innovative combination of population-wide data linkage studies as well as qualitative investigations. Studies based solely on routinely collected population-based data, such as cancer registries and/or hospital separation databases are unable to provide data on the wide range of patient, tumor, clinician and health-care system related factors that are likely to be potential confounders. Collecting information on the characteristics of the geographical areas, in addition to the characteristics of individuals living in those areas, combined with more rigorous analytical approaches, such as multilevel regression (189), will provide greater insights into the key drivers of this geographical variation.

No studies were found describing geographical differences in use of relatively new treatments, such as robotic surgery, management of metastatic prostate cancer, or treatment making decision processes. It was also unclear whether the underlying factors contributing to the key patterns in prostate cancer outcomes are similar for men living in rural and disadvantaged areas.

### Strengths and Limitations

Strengths of this review include the comprehensive search of current literature over multiple databases for studies describing international patterns in disparities along the prostate cancer continuum by rurality and residential disadvantage, quality checking of all included articles and graphical summary of results. Given it is now well-recognized that rurality and residential disadvantage interactively affect cancer outcomes, rather than acting in isolation (7, 20, 189), we also specifically assessed the impact of adjusting for residential disadvantage on variations in all six of considered outcomes by rurality and vice-versa.

All of the included quantitative studies were observational in nature, although the majority were population-based cohort studies. Around half were graded as high quality, none were graded as low-quality and the majority presented model-based estimates. Large scale population-based studies are able to identify associations between geographical measures and disparities in outcome measures. The challenge is that these associations in themselves provide only limited information in terms of the underlying reasons for observed disparities and hence how they may be reduced.

A key limitation of this review, similar to previous reviews on similar topics (4, 5, 7) was the wide variability in definition of rurality and residential disadvantage both within (especially for the USA) and across countries. For example, area-level disadvantage was variously defined in terms of a single area-level indicator (typically income), a study-specific combination over several area-based measures or a standardized country-specific composite area-based deprivation index. Moreover, the concept of rurality itself may differ between countries, such as Australia (13) and the USA (20) and smaller, more densely populated, countries, such as the UK (202). The choice of geographical measure used (20, 61, 130) and the scale over which it was measured (20, 102, 134, 184) could have impacted study findings, particularly if, for example, the area-level effects are only present in the more extreme values of the remoteness continuum.

Studies also varied widely in their data collection, analytical and reporting methods, the time frames for both diagnostic and survival intervals and covariates included in the statistical analysis. The wide heterogeneity across studies precluded a meta-analysis and limited their comparability.

Finally, despite searching multiple databases with complex queries and evaluating reference lists of identified articles, the possibility that the search term criteria, or choice of literature databases, could have inadvertently caused the exclusion of relevant articles remains.

## Conclusions

We found consistent evidence for geographical inequalities across a range of prostate cancer indicators across diverse populations, with men from disadvantaged areas facing a higher prostate cancer burden. Although there was some evidence of an association between rural residence and a higher prostate cancer burden, patterns were less consistent. There needs to be an increased focus on developing more complex research strategies to identify the key underlying drivers that can then be incorporated into evidence-based targeted interventions.

Recognizing the variation in the burden caused by prostate cancer between countries internationally, it is critical to develop strategies to at least ensure equitable access to adequate health care for all men within each country. This would ensure that all male residents of a country have the opportunity to access the same level of care regardless of where they live. Key priorities include diagnosing more aggressive disease early, optimizing informed patient-treatment decision making and providing men the best possible treatment for their disease regardless of their residential location. These tasks pose immense challenges to health providers in each country and will require collaboration over a range of concerned stakeholders.

Current evidence points to the benefit of considering health outcomes underpinned by a multi-level continuum of advantage/disadvantage where resources at an individual, social and community level serve to enable or inhibit certain behaviors and systems over a person's lifetime. Consequently, it is important to examine key variables including socioeconomic, psychosocial, cultural, and geographic characteristics in ways that reflect the complexity of people's lives. Employing such a framework will also limit the misleading reliance on the simplistic rural-urban dichotomy by highlighting the dynamic relationship between geography and disadvantage in understanding inequity in the prostate cancer burden.

## Author Contributions

All authors, JD, PB, PD, JA, NR, and SC contributed to the design of the study. PB coordinated the study. PD conducted the literature searches. PD and PB acted as reviewers. PD drafted the manuscript. PB contributed to the initial draft of the manuscript and all authors. JD, PB, PD, JA, NR, and SC refined and approved the final version of the paper. Each author has participated sufficiently in the work and takes responsibility for appropriate portions of the content. All authors have read and have given final approval of the version to be published.

### Conflict of Interest Statement

The authors declare that the research was conducted in the absence of any commercial or financial relationships that could be construed as a potential conflict of interest.

## Supplementary Material

The Supplementary Material for this article can be found online at: https://www.frontiersin.org/articles/10.3389/fonc.2019.00238/full#supplementary-material

Supplemental File 1 Systematic review literature search strategies. The file lists data-base specific search queries.

Supplemental File 2 Quality appraisal tools for included quantitative studies. The file lists the criteria and scoring system used for assessing the quality of the included cohort (Table S2.1) and case-control (Table S2.2) studies.

Supplemental File 3 Geographical measures, summary scores, overall grades and levels of evidence for included studies. The file lists the geographical measures, summary scores, overall quality scores and levels of evidence for studies included in the systematic review.

Supplemental File 4 Summary of the included studies that adjusted for both rurality and residential disadvantage.
