# Supplementary material for: Geographical Variations in Prostate Cancer Outcomes: A Systematic Review of International Evidence
Source: Front Oncol. 2019 Apr 8;9:238. doi: 10.3389/fonc.2019.00238 (PMC6463763; doi:10.3389/fonc.2019.00238)
Supplement: Supplemental File 1 — Systematic review literature search strategies. The file lists data-base specific search queries. [file Data_Sheet_1.PDF]

Supplemental file 1

Geographical variations in prostate cancer outcomes: a systematic review of international evidence

Paramita Dasgupta, Peter D Baade*, Joanne F Aitken, Nicholas Ralph, Suzanne Chambers, Jeff Dunn

***Correspondence:** Professor Peter D Baade: peterbaade@cancerqld.org.au

1 Systematic review literature search strategies

Electronic databases searched: PubMed (1998- June last week, 2018), EMBASE (1998- June last week, 2018) and CINAHL (1998- June last week, 2018)

All search queries were conducted in a stepwise manner by breaking down each question into key concepts. Each numbered step in Tables below corresponds to the query used for an individual element such as Prostate Cancer or geographical aspects and area-based disadvantage or outcome measures of interest. For each element, alternative terms were used to cover all possible synonyms for that component. Finally, the individual search queries were combined to create the final search query using BOOLEAN operators such as "AND" or "OR".

1.1 PUBMED

Search	Query
#1	("Prostate cancer"[All Fields] OR "Prostatic neoplasms"[MeSH Terms])
#2	("Rural health"[MeSH Terms] OR "urban health"[MeSH Terms] OR "Geographic inequalities"[All Fields] OR Spatial[All Fields] OR "Socioeconomic"[All Fields] OR ("vulnerable populations"[MeSH Terms] OR ("vulnerable"[All Fields] AND "populations"[All Fields]) OR "vulnerable populations"[All Fields] OR "disadvantaged"[All Fields]) OR "health literacy"[MeSH Terms] OR "health services accessibility"[MeSH Terms])
#3	("incidence"[MeSH Terms] OR "mortality"[MeSH Terms] OR "prevalence"[MeSH Terms] OR "survival"[MeSH Terms] OR "Disease progression"[MeSH Terms] OR ("PSA testing"[All Fields] OR "PSA screening"[All Fields]) OR ("therapy"[Subheading] OR "therapy"[All Fields] OR "treatment"[All Fields] OR "therapeutics"[MeSH Terms] OR "therapeutics"[All Fields]) OR "treatment complications"[All Fields] OR "prostatectomy"[All Fields] OR "brachytherapy"[All Fields] OR "treatment"[All Fields] OR "recurrence"[MeSH Terms])
#4	("Prostate cancer"[All Fields] OR "Prostatic neoplasms"[MeSH Terms]) AND ("Rural health"[MeSH Terms] OR "urban health"[MeSH Terms] OR "Geographic inequalities"[All Fields] OR Spatial[All Fields] OR "Socioeconomic"[All Fields] OR ("vulnerable populations"[MeSH Terms] OR ("vulnerable"[All Fields] AND "populations"[All Fields]) OR "vulnerable populations"[All Fields] OR "disadvantaged"[All Fields]) OR "health literacy"[MeSH Terms] OR "health services accessibility"[MeSH Terms]) AND ("incidence"[MeSH Terms] OR "mortality"[MeSH Terms] OR "prevalence"[MeSH Terms] OR "survival"[MeSH Terms] OR "Disease progression"[MeSH Terms] OR ("PSA testing"[All Fields] OR "PSA screening"[All Fields]) OR ("therapy"[Subheading] OR "therapy"[All Fields] OR "treatment"[All Fields] OR "therapeutics"[MeSH Terms] OR "therapeutics"[All Fields]) OR "treatment complications"[All Fields] OR "prostatectomy"[All Fields] OR "brachytherapy"[All Fields] OR "treatment"[All Fields] OR "recurrence"[MeSH Terms]) AND (("1998/01/01"[PDAT] : "2018/12/31"[PDAT]) AND "humans"[MeSH Terms] AND English[lang])

1.2 EMBASE

Search	Query
#1	('prostate cancer'/exp OR 'prostate cancer')
#2	('rural health care'/exp OR 'rural health care' OR 'rural population'/exp OR 'rural population' OR 'urban rural difference'/exp OR 'urban rural difference' OR 'socioeconomics'/exp OR 'medical geography'/exp OR 'medical geography' OR 'health disparity'/exp OR 'health care disparity'/exp OR 'health care disparity' OR 'socioeconomic inequality')
#3	('survival'/exp OR 'survival' OR 'cancer mortality'/exp OR 'cancer mortality' OR 'incidence' OR 'incidence'/exp OR 'prostate specific antigen' OR 'psa screening' OR 'cancer staging'/exp OR 'cancer staging' OR 'cancer grading' OR 'cancer size' OR 'metastasis'/exp OR 'metastasis' OR 'clinical practice'/exp OR 'cancer adjuvant chemotherapy'/exp OR 'cancer chemotherapy'/exp OR 'cancer radiotherapy'/exp OR 'prostatectomy' OR 'prostatectomy'/exp OR 'brachytherapy/exp' OR 'brachytherapy')
#4	(('prostate cancer'/exp OR 'prostate cancer') AND ('rural health care'/exp OR 'rural health care' OR 'rural population'/exp OR 'rural population' OR 'urban rural difference'/exp OR 'urban rural difference' OR 'socioeconomics'/exp OR 'medical geography'/exp OR 'medical geography' OR 'health disparity'/exp OR 'health care disparity'/exp OR 'health care disparity' OR 'socioeconomic inequality') AND ('survival'/exp OR 'survival' OR 'cancer mortality'/exp OR 'cancer mortality' OR 'incidence' OR 'incidence'/exp OR 'prostate specific antigen' OR 'psa screening' OR 'cancer staging'/exp OR 'cancer staging' OR 'cancer grading' OR 'cancer size' OR 'metastasis'/exp OR 'metastasis' OR 'clinical practice'/exp OR 'cancer adjuvant chemotherapy'/exp OR 'cancer chemotherapy'/exp OR 'cancer radiotherapy'/exp OR 'prostatectomy' OR 'prostatectomy'/exp OR 'brachytherapy/exp' OR 'brachytherapy')) AND [male]/lim AND [english]/lim AND [1998-2018]/py

1.3 CINAHL

Search	Query
S1	(MH "prostate cancer+") OR (MH "prostate neoplasms") OR TX "prostate cancer"
S2	(MH "Population Characteristics") OR (MH "Rural Health Centers") OR (MH "Hospitals, Rural") OR (MH "Rural Population") OR (MH "Rural Health Services") OR (MH "Rural Areas") OR (MH "Rural Health") OR (MH "Rural Health Nursing") OR (MH "Socioeconomic Factors+") OR (MH "Social Determinants of Health") OR (MH "Healthcare Disparities") OR (MH "Health Status Disparities") OR (MH "Health Services Needs and Demand+") OR TX geography OR TX rural
S3	(MH "Survival") OR (MH "Survival Analysis+") OR (MH "Mortality+") OR TX 'survival' OR ("disease free survival"[MeSH Terms]) OR ("excess mortality"[MeSH Terms]) OR ("cancer-specific survival" [MeSH Terms]) OR ("event free survival"[MeSH Terms]) OR (MH "incidence") OR TX incidence OR TX "cancer staging" OR (MH "cancer staging") OR TX "cancer screening" OR TX "prostate cancer screening" OR (MH "prostate cancer screening") OR TX prostatectomy OR (MH "prostatectomy") OR (MH "brachytherapy") OR TX brachytherapy OR ((MH "Chemotherapy, Cancer+") OR (MH "Chemotherapy, Adjuvant") OR (MH "Radiotherapy, Cancer+") OR (MH "Radiotherapy, Adjuvant")) OR TX treatment OR (MH "prostate cancer treatment") OR TX prostate cancer treatment
S4	S1 AND S2 AND S3
