# Supplementary material for: Geographical Variations in Prostate Cancer Outcomes: A Systematic Review of International Evidence
Source: Front Oncol. 2019 Apr 8;9:238. doi: 10.3389/fonc.2019.00238 (PMC6463763; doi:10.3389/fonc.2019.00238)
Supplement: Supplemental File 2 — Quality appraisal tools for included quantitative studies. The file lists the criteria and scoring system used for assessing the quality of the included cohort (Table S2.1) and case-control (Table S2.2) studies. [file Data_Sheet_2.PDF]

Supplemental file 2

Geographical variations in prostate cancer outcomes: a systematic review of international evidence

Paramita Dasgupta, Peter D Baade*, Joanne F Aitken, Nicholas Ralph, Suzanne Chambers, Jeff Dunn

***Correspondence:** Professor Peter D Baade: peterbaade@cancerqld.org.au

1 Quality appraisal tools for included quantitative studies

1.1 Table S2.1 Quality appraisal elements for cohort studies

Quality criteria	Score
**I. Selection bias**	
Representative of population of interest	2
Selected group, somewhat representative	1
Highly selected, convenient or not described	0
**II. Assessment (or measurement) of exposure and or confounding variables**	
Secure records, independent blind assessment	2
Independent assessment un-blinded; self-reported	1
No description or unclear how exposure was assessed	0
**III. Assessment (or measurement) of outcome**	
Record linkage, independent blind assessment, previously validated/reliable measures	2
Independent assessment un-blinded; self-report, novel measures	1
Novel measures (no validation/reliability tests) or assessment of outcome not described	0
**IV. Adequacy of follow-up and/or were all patients included**	
Yes (follow-up > 95%) of patients or > 95% of all patients included	2
Reasonable follow-up of all patients or all patients included (>80%)	1
≤ 80% of patients /included patients followed-up, not described or not relevant	0
**V. Adequacy of adjustment for confounding: (stratification, multivariate analysis)**	
Yes	2
Not clear or not applicable	1
No	0
**VI. If there was adjustment for residual confounding**	
Study comprehensively controls for age and additional risk factors	2
Study controls for age and most plausible additional factors	1
Minimum adjustment for plausible prognostic variables; no adjustment	0
**VII. Attrition (missing data): Was missing data handled appropriately**	
Yes	2
Not clear or not applicable	1
No	0
**VIII. Statistical methods adequate or appropriate and sufficiently described**	
Yes	2
Not clear or not applicable	1

No	0
**IX. Data presentation**	
Examples of data presented allows clear understanding of data analysis and interpretation	2
Examples provided but do not present a clear interpretation of data	1
Very little data presented or incomplete recording	0

1.2 Table S2.2 Quality appraisal elements for case-control studies

Quality criteria	Score
**I. Selection bias (Selection of cases)**	
Consecutive or obviously representative series of cases	2
Somewhat representative	1
Highly selected, convenient or not described	0
**II Selection bias (Selection of controls)**	
From same underlying population as the cases (and would be cases if had outcome)	2
Somewhat representative	1
Clear differences between cases and controls, or not described	0
**III. Assessment (or measurement) of exposure and or confounding variables**	
Secure records	2
Independent blind interview (structured interview where blind to case/control status)	1
Interview not blinded to case/control status, written self-report, no description or unclear	0
**IV. Response rate**	
High participation rates with same rate for both groups	2
Moderate participant rate (65-80%) for both groups with non-respondents described	1
Clear differences in rates between two groups	0
**V. Adequacy of case definition**	
Independent validation (e.g. medical records)	2
Subjective methods	1
No description	0
**VI. Adequacy of matching of cases and controls and/or adjustment for confounding:**	
Comprehensive matching or adjustment for all plausible prognostic variables	2
Matching or adjustment for most plausible prognostic variables	1
Minimum matching or adjustment for plausible prognostic variables; no adjustment	0
**VII. Attrition (missing data): If a concern was missing data handled appropriately**	
Yes	2
Not clear or not applicable	1
No	0
**VIII. Statistical methods adequate or appropriate and sufficiently described**	
Yes	2
Not clear or not applicable	1
No	0
**IX. Data presentation**	
Examples of data presented allows clear understanding of data analysis and interpretation	2
Examples provided but do not present a clear interpretation of data	1
Very little data presented or incomplete recording	0
