# Supplementary material for: Geographical Variations in Prostate Cancer Outcomes: A Systematic Review of International Evidence
Source: Front Oncol. 2019 Apr 8;9:238. doi: 10.3389/fonc.2019.00238 (PMC6463763; doi:10.3389/fonc.2019.00238)
Supplement: Supplemental File 3 — Geographical measures, summary scores, overall grades and levels of evidence for included studies. The file lists the geographical measures, summary scores, overall quality scores and levels of evidence for studies included in the systematic review. [file Data_Sheet_3.PDF]

Supplemental file 3

Geographical variations in prostate cancer outcomes: a systematic review of international evidence

Paramita Dasgupta, Peter D Baade*, Joanne F Aitken, Nicholas Ralph, Suzanne Chambers, Jeff Dunn

***Correspondence:** Professor Peter D Baade: peterbaade@cancerqld.org.au

1 Table 1 Geographical measures, summary scores, overall grades and levels of evidence for included studies

		Geographical measure definitions				
**Study**	**Country**	**Rurality**	**Disadvantage[1]**	**Score**	**Quality[2]**	**Level[3]**
Aarts et al, (2010) (1)	Netherlands	N/A	Composite Index	14	High	II
Aarts et al, (2013) (2)	Netherlands	N/A	Composite Index	16	High	II
Aggarwal et al, (2017) (3)	England	RUC2011	IMD2004	14	High	II
Aggarwal et al, (2018) (4)	England	RUC2011	IMD2004	12	Moderate	II
AIHW, (2013a) (5)	Australia	ARIA+	N/A	14	High	II
AIHW, (2013b) (6)	Australia	N/A	IRSD	14	High	II
Altekruse et al, (2010) (7)	USA	RUCC	N/A	14	High	II
Baade et al, (2011) (8)	Australia	ARIA+	N/A	12	Moderate	II
Baade et al, (2012) (9)	Australia	Access to services	IRSD	13	Moderate	II
Baldwin et al, (2013) (10)	USA	OMB classification	Poverty and employment	15	High	II
Belot et al, (2018) (11)	France	N/A	EDI	13	Moderate	II
Borrell et al, (2010) (12)	Spain	N/A	Composite Index	12	Moderate	IV
Boscoe et al, (2014) (13)	USA	N/A	Poverty	13	Moderate	II
Boscoe et al, (2016) (14)	USA	N/A	Poverty	15	High	II
Bravo et al, (2014) (15)	Colombia	N/A	Income	12	Moderate	II
Bryere et al, (2014) (16)	France	N/A	French DI	14	High	II
Burns et al, (2014) (17)	RoI	N/A	Carstairs Index	16	High	II
Byers et al, (2008) (18)	USA	N/A	Composite Index	14	High	III-3
Campbell et al, (2000) (19)	UK	Access to services	Carstairs Index	12	Moderate	II
Cary et al, (2016) (20)	USA	RUCC	N/A	15	High	II
Cetnar et al, (2013) (21)	USA	RUCA	N/A	14	High	III-3
Chen et al, (2016) (22)	USA	RUCC	Median income and education	13	Moderate	III-3

		Geographical measure definitions				
**Study**	**Country**	**Rurality**	**Disadvantage**[1]	**Score**	**Quality**[2]	**Level**[3]
Cheng et al, (2009) (23)	USA	N/A	Composite Index	15	High	IV
Chu et al, (2012) (24)	USA	N/A	Composite Index	13	Moderate	III-3
Clegg et al, (2009) (25)	USA	Census urban/rural areas	N/A	13	Moderate	III-3
Cobran et al, (2016) (26)	USA	Population density	Education	12	Moderate	III-3
Coleman et al, (2004) (27)	UK	N/A	Carstairs Index	14	High	II
Colli and Amling (2008) (28)	USA	Census urban/rural areas	N/A	12	Moderate	II
Coory and Baade (2005) (29)	Australia	ARIA+	N/A	12	Moderate	II
Cramb et al, (2011) (30)	Australia	ARIA+	IRSD	13	Moderate	II
Depczynski et al, (2018a) (31)	Australia	ARIA+	N/A	13	Moderate	III-3
Depczynski et al, (2018b) (32)	Australia	ARIA+	N/A	13	Moderate	III-3
DeRouen et al, (2018) (33)	USA	N/A	Composite Index	16	High	III-3
des Bordes et al, (2018) (34)	USA	RUCA	Poverty	15	High	III-3
Dey et al, (2011) (35)	Egypt	CAPMAS codes	N/A	12	Moderate	II
Du et al, (2006) (36)	USA	N/A	Composite Index	15	High	II
Ellis et al, (2018) (37)	USA	N/A	Composite Index	15	High	II
Exarchakou et al, (2018) (38)	England	N/A	IMD2004	14	High	II
Fairley et al, (2009) (39)	England	N/A	IMD2004	15	High	II
Fogleman et al, (2015) (40)	USA	RUCC	N/A	11	Moderate	II
Freeman et al, (2011) (41)	USA	N/A	Composite Index	14	High	III-3
Garcia-Gil et al, (2014) (42)	Spain	N/A	Composite Index	12	Moderate	III-3
Garg et al, (2013) (43)	USA	MSA	N/A	12	Moderate	IV
Ghali et al, (2018) (44)	USA	RUCA	N/A	16	High	II
Gilbert et al, (2011) (45)	USA	N/A	Median income and education	13	Moderate	III-3
Glaser et al, (2017) (46)	USA	RUCC	Median income	12	Moderate	III-3
Goovaerts and Xiao (2011) (47)	USA	RUCC	N/A	11	Moderate	II
Goovaerts et al, (2015) (48)	USA	N/A	Median income	13	Moderate	II
Gorday et al, (2014) (49)	Canada	N/A	Median income and education	11	Moderate	III-3

		Geographical measure definitions				
**Study**	**Country**	**Rurality**	**Disadvantage[1]**	**Score**	**Quality[2]**	**Level[3]**
Greenlee and Howe (2009) (50)	USA	N/A	Poverty	16	High	II
Guessous et al, (2016) (51)	Switzerland	Population density	N/A	13	Moderate	III-3
Haddad-Khoshkar et al, (2015) (52)	Iran	N/A	HDI	13	Moderate	II
Hagedoorn et al, (2018) (53)	Belgium	Population density	Belgian DI	14	High	II
Hall et al, (2005) (54)	Australia	ARIA	IRSD	15	High	II
Hastert et al, (2015) (55)	USA	N/A	Composite Index	11	Moderate	II
Hayen et al, (2008) (56)	Australia	Health service areas	IRSD	15	High	II
Haynes et al, (2008) (57)	New Zealand	Access to services	NZDep	14	High	II
Hellenthal et al, (2010) (58)	USA	N/A	Composite Index	15	High	II
Henry et al, (2014) (59)	Australia	Regional city (urban)	N/A	9	Moderate	III-3
Higginbotham et al, (2001) (60)	USA	Census urban/rural areas	N/A	12	Moderate	II
Hoffman et al, (2015) (61)	USA	N/A	Education	13	Moderate	II
Holmes et al, (2012) (62)	USA	RUCC, Distance to urologists	N/A	14	High	II
Holowaty et al, (2010) (63)	Canada	Census urban/rural codes	N/A	14	High	II
Hong et al, (2015) (64)	USA	N/A	Median income	14	High	II
Houston et al, (2018) (65)	USA	N/A	Poverty	12	Moderate	II
Ito et al, (2014) (66)	Japan	N/A	JDI	12	Moderate	II
Jansen et al, (2014) (67)	Germany	N/A	GIMD	14	High	II
Jarup et al, (2002) (68)	UK	Population density	Carstairs Index	12	Moderate	II
Jeffreys et al, (2009) (69)	New Zealand	N/A	NZDep	14	High	II
Jemal et al, (2002) (70)	USA	Unclear	Education and employment	11	Moderate	II
Jemal et al, (2005) (71)	USA	Population density	N/A	13	Moderate	II
Jin et al, (2018) (72)	Canada	N/A	MEDIAN INCOME	14	High	II
Jones et al, (2008a) (73)	England	Access to services,	IMD2004	14	High	II
Jones et al, (2008b) (74)	England	Access to services	IMD2004	13	Moderate	II
Jong et al, (2004) (75)	Australia	ARIA	N/A	14	High	II
Kish et al, (2014) (76)	USA	N/A	Composite Index	12	Moderate	II
Krishna et al, (2017) (77)	USA	N/A	Median income and education	12	Moderate	III-3
Krupski et al, (2005a) (78)	USA	N/A	Median income and education	15	High	II
Krupski et al, (2005b) (79)	USA	N/A	Median income and education	16	High	II

		Geographical measure definitions				
**Study**	**Country**	**Rurality**	**Disadvantage[1]**	**Score**	**Quality[2]**	**Level[3]**
Krupski et al, (2006) (80)	USA	N/A	Median income and education	13	Moderate	II
Lagace et al, (2007) (81)	Canada/Australia	MIZ (Canada) ; ARIA+ (Australia)	N/A	13	Moderate	II
Li et al, (2012) (82)	Sweden	Population density	Composite Index	16	High	II
Littlejohns et al, (2016) (83)	UK	Population density	Townsend Index	15	High	IV
Liu et al, (2001) (84)	USA	N/A	Composite Index	14	High	II
Louwman et al, (2010) (85)	Netherlands	N/A	Composite Index	14	High	II
Luce et al, (2017) (86)	West Indies	N/A	Composite Index	13	Moderate	II
Luo et al, (2015) (87)	Australia	ARIA+	IRSD	14	High	II
Lyratzopoulos et al, (2010) (88)	UK	N/A	IMD2004	15	High	II
Lyratzopoulos et al, (2013) (89)	England	N/A	IMD2004	17	High	II
Maclean et al, (2015) (90)	England	N/A	IMD2004	16	High	III-3
Mahal et al, (2015) (91)	USA	RUCC	Composite Index	15	High	II
Mahal et al, (2016) (92)	USA	RUCC	N/A	13	Moderate	III-3
Major et al, (2012) (93)	USA	Urologist density	Composite Index	12	Moderate	II
Maringe et al, (2013) (94)	England	N/A	Townsend Index	15	High	II
Mariotto et al, (2002) (95)	USA	N/A	Median income and education	12	Moderate	II
Marlow et al, (2010) (96)	USA	N/A	Median income and education	12	Moderate	III-3
Marsa et al, (2008) (97)	Denmark	Urbanization	N/A	14	High	II
Marsh et al, (2018) (98)	USA	RUCC	Median income and education	13	Moderate	III-3
Mather et al, (2006) (99)	USA	N/A	Education	11	Moderate	II
McAlister et al, (2017) (100)	Canada	Specialists/GP ratio	Median income	13	Moderate	III-3
McLafferty and Wang (2009) (101)	USA	RUCA	Median income	16	High	II
McVey et al, (2010) (102)	UK	N/A	IMD2004	12	Moderate	III-3
Meijer et al, (2013) (103)	Denmark	Population density	Employment	16	High	II
Miki et al, (2014) (104)	Japan	N/A	JDI	13	Moderate	III-3
Morgan et al, (2013) (105)	UK	N/A	SIMD	15	High	III-3
Muralidhar et al, (2016) (106)	USA	RUCC	Median income and education	13	Moderate	III-3
Nikolaidis et al, (2015) (107)	Greece	Urbanization	N/A	10	Moderate	II
Niu et al, (2010) (108)	USA	N/A	Poverty	16	High	II

		Geographical measure definitions				
**Study**	**Country**	**Rurality**	**Disadvantage[1]**	**Score**	**Quality[2]**	**Level[3]**
Obertova et al, (2016) (109)	New Zealand	MUA	N/A	12	Moderate	III-3
Ocana-Riola et al, (2004) (110)	Spain	Population density	Education and employment	15	High	II
Odisho et al, (2010) (111)	USA	RUCC	Median income, education and insurance	15	High	II
Oliver et al, (2006) (112)	USA	Census urban/rural areas	Median income, education and poverty	14	High	II
Pampalon et al, (2006) (113)	Canada	MIZ	N/A	11	Moderate	II
Papa et al, (2014) (114)	Australia	ARIA+	IRSD	15	High	II
Parikh et al, (2017) (115)	USA	Access to services	N/A	14	High	III-3
Park et al, (2016) (116)	South Korea	Population density	N/A	13	Moderate	III-3
Prasad et al, (2014) (117)	USA	Population density	Median income and education	14	High	III-3
Pukkala and Weiderpass (2002) (118)	Finland	N/A	Occupation	14	High	II
Rachet et al, (2010) (119)	England	N/A	IMD2004	14	High	II
Rand et al, (2014) (120)	USA	N/A	Median income	13	Moderate	III-3
Rogerson et al, (2006) (121)	USA	Unclear	N/A	12	Moderate	II
Rowan et al, (2008) (122)	UK	N/A	Carstairs Index (1986-1995) , IMD (1996-1999)	13	Moderate	II
Ruseckaite et al, (2016) (123)	Australia	ICS areas	N/A	12	Moderate	III-3
Rusiecki et al, (2006) (124)	USA	RUCC	N/A	12	Moderate	II
Sammon et al, (2016) (125)	USA	Population density	N/A	12	Moderate	IV
Sanderson et al, (2006) (126)	USA	N/A	Composite Index	14	High	III-3
Schwartz et al, (2003) (127)	USA	N/A	Composite Index	15	High	II
Schwartz et al, (2009) (128)	USA	N/A	Composite Index	14	High	II
Schymura et al, (2010) (129)	USA	Census urban/rural areas	Poverty and education	12	Moderate	III-3
Shack et al, (2010) (130)	UK	N/A	Carstairs Index (1986-1995) , IMD (1996-2000)	14	High	II
Shafique and Morrison (2013) (131)	UK	N/A	SIMD	15	High	II
Shafique et al, (2012a) (132)	UK	N/A	Carstairs Index	13	Moderate	II
Shafique et al, (2012b) (133)	UK	N/A	SIMD	13	Moderate	III-3
Sharma et al, (2016) (134)	Australia	Access to services	N/A	9	Moderate	III-3

Study	Country	Geographical measure definitions<br>Rurality	Disadvantage[1]	Score	Quality[2]	Level[3]
Sharp et al, (2014) (135)	Ireland (RoI, NI)	Population density	N/A	14	High	II
Singh and Jemal (2017) (136)	USA	N/A	Composite Index	14	High	II
Singh et al, (2011) (137)	USA	RUCC	Composite Index	12	Moderate	II
Skolarus et al, (2013) (138)	USA	RUCA	N/A	11	Moderate	II
Sloggett et al, (2007) (139)	UK	N/A	Carstairs Index	15	High	II
Smailyte and Kurtinaitis (2008) (140)	Lithuania	Population density	N/A	12	Moderate	II
Soto-Salgado et al, (2012) (141)	Puerto-Rico	N/A	Composite Index	14	High	II
Stanbury et al, (2016) (142)	Australia	N/A	IEO	14	High	II
Tan et al, (2018) (143)	Australia	Unclear	N/A	12	Moderate	II
Tervonen et al, (2016) (144)	Australia	ARIA+	IRSD	15	High	II
Tervonen et al, (2017a) (145)	Australia	ARIA+	IRSD	14	High	II
Tervonen et al, (2017b) (146)	Australia	N/A	IRSD	15	High	II
Thomas et al, (2017) (147)	Australia	ARIA+	IRSD	16	High	II
Trinh et al, (2016) (148)	USA	Population density	N/A	9	Moderate	IV
Tweed et al, (2018) (149)	UK	N/A	SIMD	15	High	II
Vetterlein et al, (2017) (150)	USA	RUCC	Median income and education	12	Moderate	III-3
Vicens et al, (2014) (151)	Spain	N/A	Composite Index	12	Moderate	II
Wan et al, (2011) (152)	USA	N/A	Composite Index	12	Moderate	II
Watson et al, (2017) (153)	USA	N/A	Composite Index	16	High	IV
Weiner et al, (2018) (154)	USA	N/A	Composite Index	14	High	III-3
White et al, (2011) (155)	USA	RUCA	Composite Index	16	High	II
Williams et al, (2012) (156)	USA	Population density	Median income and education	12	Moderate	III-3
Xiao et al, (2007) (157)	USA	N/A	Median income	16	High	II
Xiao et al, (2011) (158)	USA	Farm houses	N/A	12	Moderate	II
Xiao et al, (2014) (159)	USA	N/A	Median income	13	Moderate	II
Xiao et al, (2016) (160)	USA	N/A	Median income	12	Moderate	II
Yang and Hsieh (1998) (161)	Taiwan	Population density	N/A	11	Moderate	II
Yin et al, (2010) (162)	USA	N/A	Composite Index	15	High	II
Yu et al, (2008) (163)	Australia	N/A	IRSD	13	Moderate	II

Study	Country	Geographical measure definitions<br>Rurality	Disadvantage[1]	Score	Quality[2]	Level[3]
Yu et al, (2014a) (164)	Australia	ARIA+	IRSD	16	High	II
Yu et al, (2014b) (165)	USA	N/A	Composite Index	11	Moderate	II
Zahnd et al, (2017a) (166)	USA	RUCC	Income and Employment	13	Moderate	II
Zahnd et al, (2017b) (167)	USA	RUCC	N/A	12	Moderate	II
Zahnd et al, (2018) (168)	USA	RUCC	N/A	13	Moderate	II
Zhu et al, (2011) (169)	USA	Population density	N/A	10	Moderate	IV

2 References

1. Aarts MJ, van der Aa MA, Coebergh JW, Louwman WJ. Reduction of socioeconomic inequality in cancer incidence in the South of the Netherlands during 1996-2008. Eur J Cancer (2010) 46(14):2633-46 doi 10.1016/j.ejca.2010.07.039.

2. Aarts MJ, Koldewijn EL, Poortmans PM, Coebergh JW, Louwman M. The impact of socioeconomic status on prostate cancer treatment and survival in the southern Netherlands. Urology (2013) 81(3):593-9 doi 10.1016/j.urology.2012.11.011.

3. Aggarwal A, Lewis D, Sujenthiran A, Charman SC, Sullivan R, Payne H, et al. Hospital Quality Factors Influencing the Mobility of Patients for Radical Prostate Cancer Radiation Therapy: A National Population-Based Study. Int J Radiat Oncol Biol Phys (2017) 99(5):1261-70 doi 10.1016/j.ijrobp.2017.08.018.

4. Aggarwal A, Lewis D, Charman SC, Mason M, Clarke N, Sullivan R, et al. Determinants of Patient Mobility for Prostate Cancer Surgery: A Population-based Study of Choice and Competition. Eur Urol (2018) 73(6):822-5 doi 10.1016/j.eururo.2017.07.013.

5. AIHW. Cancer survival and prevalence in Australia: period estimates from 1982 to 2010. Asia Pac J Clin Oncol (2013) 9(1):29-39 doi 10.1111/ajco.12062.

6. AIHW. Cancer in Australia: Actual incidence data from 1991 to 2009 and mortality data from 1991 to 2010 with projections to 2012. Asia Pac J Clin Oncol (2013) 9(3):199-213 doi 10.1111/ajco.12127.

7. Altekruse SF, Huang L, Cucinelli JE, McNeel TS, Wells KM, Oliver MN. Spatial patterns of localized-stage prostate cancer incidence among white and black men in the southeastern United States, 1999-2001. Cancer Epidemiol Biomarkers Prev (2010) 19(6):1460-7 doi 10.1158/1055-9965.epi-09-1310.

8. Baade PD, Youlden DR, Coory MD, Gardiner RA, Chambers SK. Urban-rural differences in prostate cancer outcomes in Australia: what has changed? Med J Aust (2011) 194(6):293-6.

9. Baade PD, Youlden DR, Gardiner RA, Ferguson M, Aitken JF, Yaxley J, et al. Factors associated with treatment received by men diagnosed with prostate cancer in Queensland, Australia. BJU Int (2012) 110(11 Pt B):E712-9 doi 10.1111/j.1464-410X.2012.011533.x.

10. Baldwin LM, Andrilla CH, Porter MP, Rosenblatt RA, Patel S, Doescher MP. Treatment of early-stage prostate cancer among rural and urban patients. Cancer (2013) 119(16):3067-75 doi 10.1002/cncr.28037.

11. Belot A, Remontet L, Rachet B, Dejardin O, Charvat H, Bara S, et al. Describing the association between socioeconomic inequalities and cancer survival: methodological guidelines and illustration with population-based data. Clin Epidemiol (2018) 10:561-73 doi 10.2147/CLEP.S150848.

12. Borrell C, Mari-Dell'olmo M, Serral G, Martinez-Beneito M, Gotsens M, Members M. Inequalities in mortality in small areas of eleven Spanish cities (the multicenter MEDEA project). Health Place (2010) 16(4):703-11 doi 10.1016/j.healthplace.2010.03.002.

13. Boscoe FP, Johnson CJ, Sherman RL, Stinchcomb DG, Lin G, Henry KA. The relationship between area poverty rate and site-specific cancer incidence in the United States. Cancer (2014) 120(14):2191-8 doi 10.1002/cncr.28632.

14. Boscoe FP, Henry KA, Sherman RL, Johnson CJ. The relationship between cancer incidence, stage and poverty in the United States. Int J Cancer (2016) 139(3):607-12 doi 10.1002/ijc.30087.

15. Bravo LE, García LS, Collazos PA. Cancer survival in Cali, Colombia: A population-based study, 1995-2004. Colomb Med (2014) 45(3):110-6.

16. Bryere J, Dejardin O, Bouvier V, Colonna M, Guizard AV, Troussard X, et al. Socioeconomic environment and cancer incidence: a French population-based study in Normandy. BMC Cancer (2014) 14:87 doi 10.1186/1471-2407-14-87.

17. Burns RM, Sharp L, Sullivan FJ, Deady SE, Drummond FJ, O'Neill C. Factors Driving Inequality in Prostate Cancer Survival: A Population Based Study. PLoS One (2014) 9(9) doi 10.1371/journal.pone.0106456.

18. Byers TE, Wolf HJ, Bauer KR, Bolick-Aldrich S, Chen VW, Finch JL, et al. The impact of socioeconomic status on survival after cancer in the United States : findings from the National Program of Cancer Registries Patterns of Care Study. Cancer (2008) 113(3):582-91 doi 10.1002/cncr.23567.

19. Campbell NC, Elliott AM, Sharp L, Ritchie LD, Cassidy J, Little J. Rural factors and survival from cancer: analysis of Scottish cancer registrations. Br J Cancer (2000) 82(11):1863-6 doi 10.1054/bjoc.1999.1079.

20. Cary C, Odisho AY, Cooperberg MR. Variation in prostate cancer treatment associated with population density of the county of residence. Prostate Cancer Prostatic Dis (2016) 19:174 doi 10.1038/pcan.2015.65.

21. Cetnar JP, Hampton JM, Williamson AA, Downs T, Wang D, Owen JB, et al. Place of residence and primary treatment of prostate cancer: examining trends in rural and nonrural areas in Wisconsin. Urology (2013) 81(3):540-6 doi 10.1016/j.urology.2012.09.058.

22. Chen Y-W, Mahal BA, Muralidhar V, Nezolosky M, Beard CJ, Den RB, et al. Association Between Treatment at a High-Volume Facility and Improved Survival for Radiation-Treated Men With High-Risk Prostate Cancer. Int J Radiat Oncol Biol Phys (2016) 94(4):683-90 doi 10.1016/j.ijrobp.2015.12.008.

23. Cheng I, Witte JS, McClure LA, Shema SJ, Cockburn MG, John EM, et al. Socioeconomic status and prostate cancer incidence and mortality rates among the diverse population of California. Cancer Causes Control (2009) 20(8):1431-40 doi 10.1007/s10552-009-9369-0.

24. Chu DI, Moreira DM, Gerber L, Presti JC, Jr., Aronson WJ, Terris MK, et al. Effect of race and socioeconomic status on surgical margins and biochemical outcomes in an equal-access health

care setting: results from the Shared Equal Access Regional Cancer Hospital (SEARCH) database. Cancer (2012) 118(20):4999-5007 doi 10.1002/cncr.27456.

25. Clegg LX, Reichman ME, Miller BA, Hankey BF, Singh GK, Lin YD, et al. Impact of socioeconomic status on cancer incidence and stage at diagnosis: selected findings from the surveillance, epidemiology, and end results: National Longitudinal Mortality Study. Cancer Causes Control (2009) 20(4):417-35 doi 10.1007/s10552-008-9256-0.

26. Cobran EK, Chen RC, Overman R, Meyer AM, Kuo TM, O'Brien J, et al. Racial Differences in Diffusion of Intensity-Modulated Radiation Therapy for Localized Prostate Cancer. Am J Mens Health (2016) 10(5):399-407 doi 10.1177/1557988314568184.

27. Coleman MP, Rachet B, Woods LM, Mitry E, Riga M, Cooper N, et al. Trends and socioeconomic inequalities in cancer survival in England and Wales up to 2001. Br J Cancer (2004) 90(7):1367-73 doi 10.1038/sj.bjc.6601696.

28. Colli JL, Amling CL. Prostate cancer mortality rates compared to urologist population densities and prostate-specific antigen screening levels on a state-by-state basis in the United States of America. Prostate Cancer Prostatic Dis (2008) 11(3):247-51 doi 10.1038/pcan.2008.7.

29. Coory MD, Baade PD. Urban-rural differences in prostate cancer mortality, radical prostatectomy and prostate-specific antigen testing in Australia. Med J Aust (2005) 182(3):112-5.

30. Cramb SM, Mengersen KL, Baade PD. Identification of area-level influences on regions of high cancer incidence in Queensland, Australia: a classification tree approach. BMC Cancer (2011) 11:311 doi 10.1186/1471-2407-11-311.

31. Depczynski J, Dobbins T, Armstrong B, Lower T. Stage of diagnosis of prostate, breast and colorectal cancer in farm residents compared with other rural and urban residents in New South Wales. Aust J Rural Health (2018) 26(1):56-62 doi 10.1111/ajr.12392.

32. Depczynski J, Dobbins T, Armstrong B, Lower T. Comparison of cancer incidence in Australian farm residents 45 years and over, compared to rural non-farm and urban residents - a data linkage study. BMC Cancer (2018) 18 doi 10.1186/s12885-017-3912-2.

33. DeRouen MC, Schupp CW, Koo J, Yang J, Hertz A, Shariff-Marco S, et al. Impact of individual and neighborhood factors on disparities in prostate cancer survival. Cancer Epidemiol (2018) 53:1-11 doi 10.1016/j.canep.2018.01.003.

34. des Bordes JKA, Lopez DS, Swartz MD, Volk RJ. Sociodemographic Disparities in Cure-Intended Treatment in Localized Prostate Cancer. J Racial Ethn Health Disparities (2018) 5(1):104-10 doi 10.1007/s40615-017-0348-y.

35. Dey S, Zhang Z, Hablas A, Seifeldein IA, Ramadan M, El-Hamzawy H, et al. Geographic patterns of cancer in the population-based registry of Egypt: Possible links to environmental exposures. Cancer Epidemiol (2011) 35(3):254-64 doi 10.1016/j.canep.2010.09.010.

36. Du XL, Fang S, Coker AL, Sanderson M, Aragaki C, Cormier JN, et al. Racial disparity and socioeconomic status in association with survival in older men with local/regional stage prostate

carcinoma: findings from a large community-based cohort. Cancer (2006) 106(6):1276-85 doi 10.1002/cncr.21732.

37. Ellis L, Canchola AJ, Spiegel D, Ladabaum U, Haile R, Gomez SL. Racial and Ethnic Disparities in Cancer Survival: The Contribution of Tumor, Sociodemographic, Institutional, and Neighborhood Characteristics. J Clin Oncol (2018) 36(1):25-33 doi 10.1200/jco.2017.74.2049.

38. Exarchakou A, Rachet B, Belot A, Maringe C, Coleman MP. Impact of national cancer policies on cancer survival trends and socioeconomic inequalities in England, 1996-2013: population based study. BMJ (2018) 360:k764 doi 10.1136/bmj.k764.

39. Fairley L, Baker M, Whiteway J, Cross W, Forman D. Trends in non-metastatic prostate cancer management in the Northern and Yorkshire region of England, 2000-2006. Br J Cancer (2009) 101(11):1839-45 doi 10.1038/sj.bjc.6605424.

40. Fogleman AJ, Mueller GS, Jenkins WD. Does where you live play an important role in cancer incidence in the U.S.? Am J Cancer Res (2015) 5(7):2314-9.

41. Freeman VL, Ricardo AC, Campbell RT, Barrett RE, Warnecke RB. Association of census tract-level socioeconomic status with disparities in prostate cancer-specific survival. Cancer Epidemiol Biomarkers Prev (2011) 20(10):2150-9 doi 10.1158/1055-9965.epi-11-0344.

42. Garcia-Gil M, Elorza JM, Banque M, Comas-Cufi M, Blanch J, Ramos R, et al. Linking of primary care records to census data to study the association between socioeconomic status and cancer incidence in Southern Europe: a nation-wide ecological study. PLoS One (2014) 9(10):e109706 doi 10.1371/journal.pone.0109706.

43. Garg V, Raisch DW, Selig JP, Thompson TA. Health disparities in clinical practice patterns for prostate cancer screening by geographic regions in the United States: a multilevel modeling analysis. Prostate Cancer Prostatic Dis (2013) 16(2):193-203 doi 10.1038/pcan.2013.3.

44. Ghali F, Celaya M, Laviolette M, Ingimarsson J, Carlos H, Rees J, et al. Does Travel Time to a Radiation Facility Impact Patient Decision-Making Regarding Treatment for Prostate Cancer? A Study of the New Hampshire State Cancer Registry. J Rural Health (2018) 34:S84-S90 doi 10.1111/jrh.12224.

45. Gilbert SM, Kuo YF, Shahinian VB. Prevalent and incident use of androgen deprivation therapy among men with prostate cancer in the United States. Urol Oncol (2011) 29(6):647-53 doi 10.1016/j.urolonc.2009.09.004.

46. Glaser SM, Dohopolski MJ, Balasubramani GK, Benoit RM, Smith RP, Beriwal S. Brachytherapy boost for prostate cancer: Trends in care and survival outcomes. Brachytherapy (2017) 16(2):330-41 doi 10.1016/j.brachy.2016.12.015.

47. Goovaerts P, Xiao H. Geographical, temporal and racial disparities in late-stage prostate cancer incidence across Florida: a multiscale joinpoint regression analysis. Int J Health Geogr (2011) 10:63 doi 10.1186/1476-072X-10-63.

48. Goovaerts P, Xiao H, Gwede CK, Tan F, Huang Y, Adunlin G, et al. Impact of Age, Race and Socio-economic Status on Temporal Trends in Late-Stage Prostate Cancer Diagnosis in Florida. Spat Stat (2015) 14(Pt 100):321-37 doi 10.1016/j.spasta.2015.07.002.

49. Gorday W, Sadrzadeh H, de Koning L, Naugler C. Association of sociodemographic factors and prostate-specific antigen (PSA) testing. Clin Biochem (2014) 47(16-17):164-9 doi 10.1016/j.clinbiochem.2014.08.006.

50. Greenlee RT, Howe HL. County-level poverty and distant stage cancer in the United States. Cancer Causes Control (2009) 20(6):989-1000 doi 10.1007/s10552-009-9299-x.

51. Guessous I, Cullati S, Fedewa SA, Burton-Jeangros C, Courvoisier DS, Manor O, et al. Prostate cancer screening in Switzerland: 20-year trends and socioeconomic disparities. Prev Med (2016) 82:83-91 doi 10.1016/j.ypmed.2015.11.009.

52. Haddad-Khoshkar A, Jafari-Koshki T, Mahaki B. Investigating the Incidence of Prostate Cancer in Iran 2005 -2008 using Bayesian Spatial Ecological Regression Models. Asian Pac J Cancer Prev (2015) 16(14):5917-21 doi 10.7314/APJCP.2015.16.14.5917.

53. Hagedoorn P, Vandenheede H, Vanthomme K, Gadeyne S. Socioeconomic position, population density and site-specific cancer mortality: A multilevel analysis of Belgian adults, 2001-2011. Int J Cancer (2018) 142(1):23-35 doi 10.1002/ijc.31031.

54. Hall SE, Holman CD, Wisniewski ZS, Semmens J. Prostate cancer: socio-economic, geographical and private-health insurance effects on care and survival. BJU Int (2005) 95(1):51-8 doi 10.1111/j.1464-410X.2005.05248.x.

55. Hastert TA, Beresford SA, Sheppard L, White E. Disparities in cancer incidence and mortality by area-level socioeconomic status: a multilevel analysis. J Epidemiol Community Health (2015) 69(2):168-76 doi 10.1136/jech-2014-204417.

56. Hayen A, Smith DP, Patel MI, O'Connell DL. Patterns of surgical care for prostate cancer in NSW, 1993-2002: rural/urban and socio-economic variation. Aust N Z J Public Health (2008) 32(5):417-20 doi 10.1111/j.1753-6405.2008.00272.x.

57. Haynes R, Pearce J, Barnett R. Cancer survival in New Zealand: ethnic, social and geographical inequalities. Soc Sci Med (2008) 67(6):928-37 doi 10.1016/j.socscimed.2008.05.005.

58. Hellenthal NJ, Parikh-Patel A, Bauer K, Ralph W, deVere W, Koppie TM. Men of higher socioeconomic status have improved outcomes after radical prostatectomy for localized prostate cancer. Urology (2010) 76(6):1409-13 doi 10.1016/j.urology.2010.03.024.

59. Henry MJ, Jones P, Morrissy K, Matheson LM, Pitson G, Healy P, et al. Radiotherapy in the Barwon South Western Region: a rural perspective. J Med Imaging Radiat Oncol (2014) 58(5):612-7 doi 10.1111/1754-9485.12208.

60. Higginbotham JC, Moulder J, Currier M. Rural v. urban aspects of cancer: first-year data from the Mississippi Central Cancer Registry. Fam Community Health (2001) 24(2):1-9.

61. Hoffman RM, Shi Y, Freedland SJ, Keating NL, Walter LC. Treatment patterns for older veterans with localized prostate cancer. Cancer Epidemiol (2015) 39(5):769-77 doi 10.1016/j.canep.2015.07.005.

62. Holmes JA, Carpenter WR, Wu Y, Hendrix LH, Peacock S, Massing M, et al. Impact of distance to a urologist on early diagnosis of prostate cancer among black and white patients. J Urol (2012) 187(3):883-8 doi 10.1016/j.juro.2011.10.156.

63. Holowaty EJ, Norwood TA, Wanigaratne S, Abellan JJ, Beale L. Feasibility and utility of mapping disease risk at the neighbourhood level within a Canadian public health unit: an ecological study. Int J Health Geogr (2010) 9:21 doi 10.1186/1476-072x-9-21.

64. Hong X, Fei T, Adunlin G, Ali AA, Goovaerts P, Gwede CK, et al. Factors Associated with Overall Survival Prostate Cancer in Florida: A Multilevel Analysis. J Health Care Poor Underserved (2015) 26(1):266-77 doi 10.1353/hpu.2015.0007.

65. Houston KA, King J, Li J, Jemal A. Trends in Prostate Cancer Incidence Rates and Prevalence of Prostate Specific Antigen Screening by Socioeconomic Status and Regions in the United States, 2004 to 2013. J Urol (2018) 199(3):676-81 doi 10.1016/j.juro.2017.09.103.

66. Ito Y, Nakaya T, Nakayama T, Miyashiro I, Ioka A, Tsukuma H, et al. Socioeconomic inequalities in cancer survival: a population-based study of adult patients diagnosed in Osaka, Japan, during the period 1993-2004. Acta Oncol (2014) 53(10):1423-33 doi 10.3109/0284186X.2014.912350.

67. Jansen L, Eberle A, Emrich K, Gondos A, Holleczek B, Kajüter H, et al. Socioeconomic deprivation and cancer survival in Germany: An ecological analysis in 200 districts in Germany. Int J Cancer (2014) 134(12):2951-60 doi 10.1002/ijc.28624.

68. Jarup L, Best N, Toledano MB, Wakefield J, Elliott P. Geographical epidemiology of prostate cancer in Great Britain. Int J Cancer (2002) 97(5):695-9 doi 10.1002/ijc.10113.

69. Jeffreys M, Sarfati D, Stevanovic V, Tobias M, Lewis C, Pearce N, et al. Socioeconomic Inequalities in Cancer Survival in New Zealand: The Role of Extent of Disease at Diagnosis. Cancer Epidemiol Biomarkers Prev (2009) 18(3):915 doi 10.1158/1055-9965.EPI-08-0685.

70. Jemal A, Kulldorff M, Devesa SS, Hayes RB, Fraumeni JF, Jr. A geographic analysis of prostate cancer mortality in the United States, 1970-89. Int J Cancer (2002) 101(2):168-74 doi 10.1002/ijc.10594.

71. Jemal A, Ward E, Wu X, Martin HJ, McLaughlin CC, Thun MJ. Geographic patterns of prostate cancer mortality and variations in access to medical care in the United States. Cancer Epidemiol Biomarkers Prev (2005) 14(3):590-5 doi 10.1158/1055-9965.EPI-04-0522.

72. Jin CJ, Hanna TP, Cook EF, Miao Q, Brundage MD. Variation in Radiotherapy Referral and Treatment for High-risk Pathological Features after Radical Prostatectomy: Results from a Population-based Study. Clin Oncol (2018) 30(1):47-56 doi 10.1016/j.clon.2017.10.009.

73. Jones AP, Haynes R, Sauerzapf V, Crawford SM, Zhao H, Forman D. Travel times to health care and survival from cancers in Northern England. Eur J Cancer (2008) 44(2):269-74 doi 10.1016/j.ejca.2007.07.028.

74. Jones AP, Haynes R, Sauerzapf V, Crawford SM, Zhao H, Forman D. Travel time to hospital and treatment for breast, colon, rectum, lung, ovary and prostate cancer. Eur J Cancer (2008) 44(7):992-9 doi 10.1016/j.ejca.2008.02.001.

75. Jong KE, Smith DP, Yu XQ, O'Connell DL, Goldstein D, Armstrong BK. Remoteness of residence and survival from cancer in New South Wales. Med J Aust (2004) 180(12):618-22.

76. Kish JK, Yu M, Percy-Laurry A, Altekruse SF. Racial and ethnic disparities in cancer survival by neighborhood socioeconomic status in Surveillance, Epidemiology, and End Results (SEER) Registries. J Natl Cancer Inst Monogr (2014) 2014(49):236-43 doi 10.1093/jncimonographs/lgu020.

77. Krishna S, Fan Y, Jarosek S, Adejoro O, Chamie K, Konety B. Racial Disparities in Active Surveillance for Prostate Cancer. J Urol (2017) 197(2):342-9 doi 10.1016/j.juro.2016.08.104.

78. Krupski TL, Kwan L, Afifi AA, Litwin MS. Geographic and socioeconomic variation in the treatment of prostate cancer. J Clin Oncol (2005) 23(31):7881-8 doi 10.1200/jco.2005.08.755.

79. Krupski TL, Kwan L, Litwin MS. Sociodemographic factors associated with postprostatectomy radiotherapy. Prostate Cancer Prostatic Dis (2005) 8(2):184-8 doi 10.1038/sj.pcan.4500791.

80. Krupski TL, Saigal CS, Hanley J, Schonlau M, Litwin MS. Patterns of care for men with prostate cancer after failure of primary treatment. Cancer (2006) 107(2):258-65 doi 10.1002/cncr.21981.

81. Lagace C, Desmeules M, Pong RW, Heng D. Non-communicable disease and injury-related mortality in rural and urban places of residence: a comparison between Canada and Australia. Can J Public Health (2007) 98 Suppl 1:S62-9.

82. Li X, Sundquist K, Sundquist J. Neighborhood deprivation and prostate cancer mortality: a multilevel analysis from Sweden. Prostate Cancer Prostatic Dis (2012) 15:128 doi 10.1038/pcan.2011.46.

83. Littlejohns TJ, Travis RC, Key TJ, Allen NE. Lifestyle factors and prostate-specific antigen (PSA) testing in UK Biobank: Implications for epidemiological research. Cancer Epidemiol (2016) 45:40-6 doi 10.1016/j.canep.2016.09.010.

84. Liu L, Cozen W, Bernstein L, Ross RK, Deapen D. Changing relationship between socioeconomic status and prostate cancer incidence. J Natl Cancer Inst (2001) 93(9):705-9.

85. Louwman WJ, Aarts MJ, Houterman S, van Lenthe FJ, Coebergh JW, Janssen-Heijnen ML. A 50% higher prevalence of life-shortening chronic conditions among cancer patients with low socioeconomic status. Br J Cancer (2010) 103(11):1742-8 doi 10.1038/sj.bjc.6605949.

86. Luce D, Michel S, Dugas J, Bhakkan B, Menvielle G, Joachim C, et al. Disparities in cancer incidence by area-level socioeconomic status in the French West Indies. Cancer Causes Control (2017) 28(11):1305-12 doi 10.1007/s10552-017-0946-3.

87. Luo Q, Yu XQ, Smith DP, O'Connell DL. A population-based study of progression to metastatic prostate cancer in Australia. Cancer Epidemiol (2015) 39(4):617-22 doi 10.1016/j.canep.2015.04.013.

88. Lyratzopoulos G, Barbiere JM, Greenberg DC, Wright KA, Neal DE. Population based time trends and socioeconomic variation in use of radiotherapy and radical surgery for prostate cancer in a UK region: continuous survey. BMJ (2010) 340:c1928 doi 10.1136/bmj.c1928.

89. Lyratzopoulos G, Abel GA, Brown CH, Rous BA, Vernon SA, Roland M, et al. Socio-demographic inequalities in stage of cancer diagnosis: evidence from patients with female breast, lung, colon, rectal, prostate, renal, bladder, melanoma, ovarian and endometrial cancer. Ann Oncol (2013) 24(3):843-50 doi 10.1093/annonc/mds526.

90. Maclean R, Jeffreys M, Ives A, Jones T, Verne J, Ben-Shlomo Y. Primary care characteristics and stage of cancer at diagnosis using data from the national cancer registration service, quality outcomes framework and general practice information. BMC Cancer (2015) 15:500 doi 10.1186/s12885-015-1497-1.

91. Mahal BA, Cooperberg MR, Aizer AA, Ziehr DR, Hyatt AS, Choueiri TK, et al. Who bears the greatest burden of aggressive treatment of indolent prostate cancer? Am J Med (2015) 128(6):609-16 doi 10.1016/j.amjmed.2014.12.030.

92. Mahal BA, Chen YW, Muralidhar V, Mahal AR, Choueiri TK, Hoffman KE, et al. National sociodemographic disparities in the treatment of high-risk prostate cancer: Do academic cancer centers perform better than community cancer centers? Cancer (2016) 122(21):3371-7 doi 10.1002/cncr.30205.

93. Major JM, Norman Oliver M, Doubeni CA, Hollenbeck AR, Graubard BI, Sinha R. Socioeconomic status, healthcare density, and risk of prostate cancer among African American and Caucasian men in a large prospective study. Cancer Causes Control (2012) 23(7):1185-91 doi 10.1007/s10552-012-9988-8.

94. Maringe C, Mangtani P, Rachet B, Leon DA, Coleman MP, dos Santos Silva I. Cancer incidence in South Asian migrants to England, 1986-2004: unraveling ethnic from socioeconomic differentials. Int J Cancer (2013) 132(8):1886-94 doi 10.1002/ijc.27826.

95. Mariotto A, Capocaccia R, Verdecchia A, Micheli A, Feuer EJ, Pickle L, et al. Projecting SEER cancer survival rates to the US: an ecological regression approach. Cancer Causes Control (2002) 13(2):101-11.

96. Marlow NM, Halpern MT, Pavluck AL, Ward EM, Chen AY. Disparities associated with advanced prostate cancer stage at diagnosis. J Health Care Poor Underserved (2010) 21(1):112-31 doi 10.1353/hpu.0.0253.

97. Marsa K, Johnsen NF, Bidstrup PE, Johannesen-Henry CT, Friis S. Social inequality and incidence of and survival from male genital cancer in a population-based study in Denmark, 1994-2003. Eur J Cancer (2008) 44(14):2018-29 doi 10.1016/j.ejca.2008.06.012.

98. Marsh S, Walters RW, Silberstein PT. Survival Outcomes of Radical Prostatectomy Versus Radiotherapy in Intermediate-Risk Prostate Cancer: A NCDB Study. Clin Genitourin Cancer (2018) 16(1):E39-E46 doi 10.1016/j.clgc.2017.07.029.

99. Mather FJ, Chen VW, Morgan LH, Correa CN, Shaffer JG, Srivastav SK, et al. Hierarchical modeling and other spatial analyses in prostate cancer incidence data. Am J Prev Med (2006) 30(2 Suppl):S88-100 doi 10.1016/j.amepre.2005.09.012.

100. McAlister FA, Lin M, Bakal J, Dean S. Frequency of low-value care in Alberta, Canada: a retrospective cohort study. BMJ Qual Saf (2018) 27(5):340-6 doi 10.1136/bmjqs-2017-006778.

101. McLafferty S, Wang F. Rural reversal? Rural-urban disparities in late-stage cancer risk in Illinois. Cancer (2009) 115(12):2755-64 doi 10.1002/cncr.24306.

102. McVey GP, McPhail S, Fowler S, McIntosh G, Gillatt D, Parker CC. Initial management of low-risk localized prostate cancer in the UK: analysis of the British Association of Urological Surgeons Cancer Registry. BJU Int (2010) 106(8):1161-4 doi 10.1111/j.1464-410X.2010.09288.x.

103. Meijer M, Bloomfield K, Engholm G. Neighbourhoods matter too: the association between neighbourhood socioeconomic position, population density and breast, prostate and lung cancer incidence in Denmark between 2004 and 2008. J Epidemiol Community Health (2013) 67(1):6-13 doi 10.1136/jech-2011-200192.

104. Miki Y, Inoue M, Ikeda A, Sawada N, Nakaya T, Shimazu T, et al. Neighborhood deprivation and risk of cancer incidence, mortality and survival: results from a population-based cohort study in Japan. PLoS One (2014) 9(9):e106729 doi 10.1371/journal.pone.0106729.

105. Morgan RM, Steele RJ, Nabi G, McCowan C. Socioeconomic variation and prostate specific antigen testing in the community: a United Kingdom based population study. J Urol (2013) 190(4):1207-12 doi 10.1016/j.juro.2013.04.044.

106. Muralidhar V, Rose BS, Chen YW, Nezolosky MD, Nguyen PL. Association Between Travel Distance and Choice of Treatment for Prostate Cancer: Does Geography Reduce Patient Choice? Int J Radiat Oncol Biol Phys (2016) 96(2):313-7 doi 10.1016/j.ijrobp.2016.05.022.

107. Nikolaidis C, Tentes I, Lialiaris T, Constantinidis TC, Kortsaris A. Regional disparities in cancer mortality across the rural-urban axis: a case study from north-eastern Greece. Rural Remote Health (2015) 15(3):3013.

108. Niu X, Pawlish KS, Roche LM. Cancer survival disparities by race/ethnicity and socioeconomic status in New Jersey. J Health Care Poor Underserved (2010) 21(1):144-60 doi 10.1353/hpu.0.0263.

109. Obertova Z, Hodgson F, Scott-Jones J, Brown C, Lawrenson R. Rural-Urban Differences in Prostate-Specific Antigen (PSA) Screening and Its Outcomes in New Zealand. J Rural Health (2016) 32(1):56-62 doi 10.1111/jrh.12127.

110. Ocana-Riola R, Sanchez-Cantalejo C, Rosell J, Sanchez-Cantalejo E, Daponte A. Socio-economic level, farming activities and risk of cancer in small areas of Southern Spain. Eur J Epidemiol (2004) 19(7):643-50.

111. Odisho AY, Cooperberg MR, Fradet V, Ahmad AE, Carroll PR. Urologist density and county-level urologic cancer mortality. J Clin Oncol (2010) 28(15):2499-504 doi 10.1200/jco.2009.26.9597.

112. Oliver MN, Smith E, Siadaty M, Hauck FR, Pickle LW. Spatial analysis of prostate cancer incidence and race in Virginia, 1990-1999. Am J Prev Med (2006) 30(2 Suppl):S67-76 doi 10.1016/j.amepre.2005.09.008.

113. Pampalon R, Martinez J, Hamel D. Does living in rural areas make a difference for health in Quebec? Health Place (2006) 12(4):421-35 doi 10.1016/j.healthplace.2005.04.002.

114. Papa N, Lawrentschuk N, Muller D, MacInnis R, Ta A, Severi G, et al. Rural residency and prostate cancer specific mortality: results from the Victorian Radical Prostatectomy Register. Aust N Z J Public Health (2014) 38(5):449-54 doi 10.1111/1753-6405.12210.

115. Parikh RR, Kim S, Stein MN, Haffty BG, Kim IY, Goyal S. Trends in active surveillance for very low-risk prostate cancer: do guidelines influence modern practice? Cancer Med (2017) 6(10):2410-8 doi 10.1002/cam4.1132.

116. Park J, Suh B, Shin DW, Hong JH, Ahn H. Changing Patterns of Primary Treatment in Korean Men with Prostate Cancer Over 10 Years: A Nationwide Population Based Study. Cancer Res Treat (2016) 48(3):899-906 doi 10.4143/crt.2015.212.

117. Prasad SM, Eggener SE, Lipsitz SR, Irwin MR, Ganz PA, Hu JC. Effect of depression on diagnosis, treatment, and mortality of men with clinically localized prostate cancer. J Clin Oncol (2014) 32(23):2471-8 doi 10.1200/jco.2013.51.1048.

118. Pukkala E, Weiderpass E. Socio-economic differences in incidence rates of cancers of the male genital organs in Finland, 1971-95. Int J Cancer (2002) 102(6):643-8 doi 10.1002/ijc.10749.

119. Rachet B, Ellis L, Maringe C, Chu T, Nur U, Quaresma M, et al. Socioeconomic inequalities in cancer survival in England after the NHS cancer plan. Br J Cancer (2010) 103(4):446-53 doi 10.1038/sj.bjc.6605752.

120. Rand AE, Agarwal A, Ahuja D, Ngo T, Qureshi MM, Gupta A, et al. Patient demographic characteristics and disease stage as drivers of disparities in mortality in prostate cancer patients who receive care at a safety net academic medical center. Clin Genitourin Cancer (2014) 12(6):455-60 doi 10.1016/j.clgc.2014.04.005.

121. Rogerson PA, Sinha G, Han D. Recent changes in the spatial pattern of prostate cancer in the U.S. Am J Prev Med (2006) 30(2 Suppl):S50-9 doi 10.1016/j.amepre.2005.09.006.

122. Rowan S, Rachet B, Alexe DM, Cooper N, Coleman MP. Survival from prostate cancer in England and Wales up to 2001. Br J Cancer (2008) 99:S75-S7 doi 10.1038/sj.bjc.6604595.

123. Ruseckaite R, Sampurno F, Millar J, Frydenberg M, Evans S. Diagnostic and treatment factors associated with poor survival from prostate cancer are differentially distributed between regional and metropolitan Victoria, Australia. BMC Urol (2016) 16(1):54 doi 10.1186/s12894-016-0172-4.

124. Rusiecki JA, Kulldorff M, Nuckols JR, Song C, Ward MH. Geographically based investigation of prostate cancer mortality in four U.S. Northern Plain states. Am J Prev Med (2006) 30(2 Suppl):S101-8 doi 10.1016/j.amepre.2005.09.005.

125. Sammon J, Dalela D, Abdollah F, Choueiri T, Han P, Hansen M, et al. Determinants of prostate specific antigen screening among black men in the United States in the contemporary era. J Urol (2016) 195(4):e247 doi 10.1016/j.juro.2015.11.023.

126. Sanderson M, Coker AL, Perez A, Du XL, Peltz G, Fadden MK. A multilevel analysis of socioeconomic status and prostate cancer risk. Ann Epidemiol (2006) 16(12):901-7 doi 10.1016/j.annepidem.2006.02.006.

127. Schwartz KL, Crossley-May H, Vigneau FD, Brown K, Banerjee M. Race, socioeconomic status and stage at diagnosis for five common malignancies. Cancer Causes Control (2003) 14(8):761-6.

128. Schwartz K, Powell IJ, Underwood W, 3rd, George J, Yee C, Banerjee M. Interplay of race, socioeconomic status, and treatment on survival of patients with prostate cancer. Urology (2009) 74(6):1296-302 doi 10.1016/j.urology.2009.02.058.

129. Schymura MJ, Kahn AR, German RR, Hsieh MC, Cress RD, Finch JL, et al. Factors associated with initial treatment and survival for clinically localized prostate cancer: results from the CDC-NPCR Patterns of Care Study (PoC1). BMC Cancer (2010) 10:152 doi 10.1186/1471-2407-10-152.

130. Shack LG, Rachet B, Brewster DH, Coleman MP. Socioeconomic inequalities in cancer survival in Scotland 1986-2000. Br J Cancer (2007) 97(7):999-1004.

131. Shafique K, Morrison DS. Socio-economic inequalities in survival of patients with prostate cancer: role of age and Gleason grade at diagnosis. PLoS One (2013) 8(2):e56184 doi 10.1371/journal.pone.0056184.

132. Shafique K, Oliphant R, Morrison DS. The impact of socio-economic circumstances on overall and grade-specific prostate cancer incidence: a population-based study. Br J Cancer (2012) 107(3):575-82 doi 10.1038/bjc.2012.289.

133. Shafique K, Proctor MJ, McMillan DC, Qureshi K, Leung H, Morrison DS. Systemic inflammation and survival of patients with prostate cancer: evidence from the Glasgow Inflammation Outcome Study. Prostate Cancer Prostatic Dis (2012) 15(2):195-201 doi 10.1038/pcan.2011.60.

134. Sharma DK, Vangaveti VN, Larkins S. Geographical access to radiation therapy in North Queensland: a retrospective analysis of patient travel to radiation therapy before and after the opening of an additional radiotherapy facility. Rural Remote Health (2016) 16(1):3640.

135. Sharp L, Donnelly D, Hegarty A, Carsin AE, Deady S, McCluskey N, et al. Risk of several cancers is higher in urban areas after adjusting for socioeconomic status. Results from a two-country population-based study of 18 common cancers. J Urban Health (2014) 91(3):510-25 doi 10.1007/s11524-013-9846-3.

136. Singh GK, Jemal A. Socioeconomic and Racial/Ethnic Disparities in Cancer Mortality, Incidence, and Survival in the United States, 1950-2014: Over Six Decades of Changing Patterns and Widening Inequalities. J Environ Public Health (2017) 2017:2819372 doi 10.1155/2017/2819372.

137. Singh GK, Williams SD, Siahpush M, Mulhollen A. Socioeconomic, Rural-Urban, and Racial Inequalities in US Cancer Mortality: Part I-All Cancers and Lung Cancer and Part II-Colorectal, Prostate, Breast, and Cervical Cancers. J Cancer Epidemiol (2011) 2011:107497 doi 10.1155/2011/107497.

138. Skolarus TA, Chan S, Shelton JB, Antonio AL, Sales AE, Malin JL, et al. Quality of prostate cancer care among rural men in the Veterans Health Administration. Cancer (2013) 119(20):3629-35 doi 10.1002/cncr.28275.

139. Sloggett A, Young H, Grundy E. The association of cancer survival with four socioeconomic indicators: a longitudinal study of the older population of England and Wales 1981-2000. BMC Cancer (2007) 7 doi 10.1186/1471-2407-7-20.

140. Smailyte G, Kurtinaitis J. Cancer mortality differences among urban and rural residents in Lithuania. BMC Public Health (2008) 8:56 doi 10.1186/1471-2458-8-56.

141. Soto-Salgado M, Suarez E, Torres-Cintron M, Pettaway CA, Colon V, Ortiz AP. Prostate cancer incidence and mortality among Puerto Ricans: an updated analysis comparing men in Puerto Rico with US racial/ethnic groups. P R Health Sci J (2012) 31(3):107-13.

142. Stanbury JF, Baade PD, Yu Y, Yu XQ. Impact of geographic area level on measuring socioeconomic disparities in cancer survival in New South Wales, Australia: A period analysis. Cancer Epidemiol (2016) 43:56-62 doi 10.1016/j.euf.2017.03.008.

143. Tan L, Wang LL, Ranasinghe W, Persad R, Bolton D, Lawrentschuk N, et al. Survival outcomes of younger men (< 55 years) undergoing radical prostatectomy. Prostate International (2018) 6(1):31-5 doi 10.1016/j.prnil.2017.07.002.

144. Tervonen HE, Walton R, Roder D, You H, Morrell S, Baker D, et al. Socio-demographic disadvantage and distant summary stage of cancer at diagnosis--A population-based study in New South Wales. Cancer Epidemiol (2016) 40(2):87-94 doi 10.1016/j.canep.2015.10.032.

145. Tervonen HE, Aranda S, Roder D, You H, Walton R, Morrell S, et al. Cancer survival disparities worsening by socio-economic disadvantage over the last 3 decades in new South Wales, Australia. BMC Public Health (2017) 17(1):691 doi 10.1186/s12889-017-4692-y.

146. Tervonen HE, Morrell S, Aranda S, Roder D, You H, Niyonsenga T, et al. The impact of geographic unit of analysis on socioeconomic inequalities in cancer survival and distant summary stage - a population-based study. Aust N Z J Public Health (2017) 41(2):130-6 doi 10.1111/1753-6405.12608.

147. Thomas AA, Pearce A, Sharp L, Gardiner RA, Chambers S, Aitken J, et al. Socioeconomic disadvantage but not remoteness affects short-term survival in prostate cancer: A population-based study using competing risks. Asia Pac J Clin Oncol (2017) 13(2):e31-e40 doi 10.1111/ajco.12570.

148. Trinh QD, Li H, Meyer CP, Hanske J, Choueiri TK, Reznor G, et al. Determinants of cancer screening in Asian-Americans. Cancer Causes Control (2016) 27(8):989-98 doi 10.1007/s10552-016-0776-8.

149. Tweed EJ, Allardice GM, McLoone P, Morrison DS. Socio-economic inequalities in the incidence of four common cancers: a population-based registry study. Public Health (2018) 154:1-10 doi 10.1016/j.puhe.2017.10.005.

150. Vetterlein MW, Loppenberg B, Karabon P, Dalela D, Jindal T, Sood A, et al. Impact of travel distance to the treatment facility on overall mortality in US patients with prostate cancer. Cancer (2017) 123(17):3241-52 doi 10.1002/cncr.30744.

151. Vicens GR, Zafra MS, Moreno-Crespi J, Ferrer BC, Marcos-Gragera R. Incidence variation of prostate and cervical cancer according to socioeconomic level in the Girona Health Region. BMC Public Health (2014) 14:1079 doi 10.1186/1471-2458-14-1079.

152. Wan N, Zhan FB, Cai Z. Socioeconomic disparities in prostate cancer mortality and the impact of geographic scale. South Med J (2011) 104(8):553-9 doi 10.1097/SMJ.0b013e31821f99ff.

153. Watson M, Grande D, Radhakrishnan A, Mitra N, Ward KR, Pollack CE. Racial Differences in Prostate Cancer Treatment: The Role of Socioeconomic Status. Ethn Dis (2017) 27(3):201-8 doi 10.18865/ed.27.3.201.

154. Weiner AB, Matulewicz RS, Tosoian JJ, Feinglass JM, Schaeffer EM. The effect of socioeconomic status, race, and insurance type on newly diagnosed metastatic prostate cancer in the United States (2004–2013). Urologic Oncology: Seminars and Original Investigations (2018) 36(3):91.e1-.e6 doi 10.1016/j.urolonc.2017.10.023.

155. White A, Coker AL, Du XL, Eggleston KS, Williams M. Racial/ethnic disparities in survival among men diagnosed with prostate cancer in Texas. Cancer (2011) 117(5):1080-8 doi 10.1002/cncr.25671.

156. Williams SB, Lei Y, Nguyen PL, Gu X, Lipsitz SR, Yu HY, et al. Comparative effectiveness of cryotherapy vs brachytherapy for localised prostate cancer. BJU Int (2012) 110(2 Pt 2):E92-8 doi 10.1111/j.1464-410X.2011.10775.x.

157. Xiao H, Gwede CK, Kiros G, Milla K. Analysis of prostate cancer incidence using geographic information system and multilevel modeling. J Natl Med Assoc (2007) 99(3):218-25.

158. Xiao H, Tan F, Goovaerts P. Racial and geographic disparities in late-stage prostate cancer diagnosis in Florida. J Health Care Poor Underserved (2011) 22(4 Suppl):187-99 doi 10.1353/hpu.2011.0155.

159. Xiao H, Tan F, Goovaerts P, Ali A, Adunlin G, Gwede CK, et al. Multilevel Factors Associated With Overall Mortality for Men Diagnosed With Prostate Cancer in Florida. Am J Mens Health (2014) 8(4):316-26 doi 10.1177/1557988313512862.

160. Xiao H, Tan F, Goovaerts P, Adunlin G, Ali AA, Gwede CK, et al. Impact of Comorbidities on Prostate Cancer Stage at Diagnosis in Florida. Am J Mens Health (2016) 10(4):285-95 doi 10.1177/1557988314564593.

161. Yang CY, Hsieh YL. The relationship between population density and cancer mortality in Taiwan. Jpn J Cancer Res (1998) 89(4):355-60 doi S0910505098800501 [pii].

162. Yin D, Morris C, Allen M, Cress R, Bates J, Liu L. Does socioeconomic disparity in cancer incidence vary across racial/ethnic groups? Cancer Causes Control (2010) 21(10):1721-30 doi 10.1007/s10552-010-9601-y.

163. Yu XQ, O'Connell DL, Gibberd RW, Armstrong BK. Assessing the impact of socio-economic status on cancer survival in New South Wales, Australia 1996-2001. Cancer Causes Control (2008) 19(10):1383-90 doi 10.1007/s10552-008-9210-1.

164. Yu XQ, Luo Q, Smith DP, O'Connell DL, Baade PD. Geographic variation in prostate cancer survival in New South Wales. Med J Aust (2014) 200(10):586-90.

165. Yu M, Tatalovich Z, Gibson JT, Cronin KA. Using a composite index of socioeconomic status to investigate health disparities while protecting the confidentiality of cancer registry data. Cancer Causes Control (2014) 25(1):81-92 doi 10.1007/s10552-013-0310-1.

166. Zahnd WE, Jenkins WD, Mueller-Luckey GS. Cancer Mortality in the Mississippi Delta Region: Descriptive Epidemiology and Needed Future Research and Interventions. J Health Care Poor Underserved (2017) 28(1):315-28 doi 10.1353/hpu.2017.0025.

167. Zahnd WE, James AS, Jenkins WD, Izadi SR, Fogleman AJ, Steward DE, et al. Rural-Urban Differences in Cancer Incidence and Trends in the United States. Cancer Epidemiol Biomarkers Prev (2017):in press doi 10.1158/1055-9965.EPI-17-0430.

168. Zahnd WE, Jenkins WD, James AS, Izadi SR, Steward DE, Fogleman AJ, et al. Utility and Generalizability of Multi-State, Population-Based Cancer Registry Data for Rural Cancer Surveillance Research in the United States. Cancer Epidemiol Biomarkers Prev (2018):in press doi 10.1158/1055-9965.EPI-17-1087.

169. Zhu Y, Sorkin JD, Dwyer D, Groves C, Steinberger EK. Predictors of repeated PSA testing among black and white men from the Maryland Cancer Survey, 2006. Prev Chronic Dis (2011) 8(5):A114.
