# Supplementary material for: Geographical Variations in Prostate Cancer Outcomes: A Systematic Review of International Evidence
Source: Front Oncol. 2019 Apr 8;9:238. doi: 10.3389/fonc.2019.00238 (PMC6463763; doi:10.3389/fonc.2019.00238)
Supplement: Supplemental File 4 — Summary of the included studies that adjusted for both rurality and residential disadvantage. [file Data_Sheet_4.PDF]

Supplemental file 4

Geographical variations in prostate cancer outcomes: a systematic review of international evidence

Paramita Dasgupta, Peter D Baade*, Joanne F Aitken, Nicholas Ralph, Suzanne Chambers, Jeff Dunn

***Correspondence:** Professor Peter D Baade: peterbaade@cancerqld.org.au

1 Table 8 Summary of included studies that adjusted for both rurality and disadvantage

Author, year	Location	Additional covariates[1]	Findings [95% confidence interval in brackets][2]	
			**Rural versus Urban**	**Disadvantaged versus Affluent**
		PSA testing *(2 of 11 included studies)*		
McAlister et al, (2017) (1)	Alberta, Canada	age, comorbidity	OR (unit increase specialist/GP ratio) 7.79 [5.13-11.29]	OR 0.85 [0.81-0.88][3]
Littlejohns et al, (2016) (2)	UK	age, comorbidity, individual socio-demographics, lifestyle	OR 1.01 [0.98-1.04]	OR 0.84 [0.81-0.97]
		Prostate cancer Incidence *(4 of 23 included studies)*		
Major et al, (2012) (3)	USA	age, individual demographics, lifestyle, by ethnicity	HR W 0.90 [0.84-0.96]<br>AA 1.11 [0.80-1.54]	HR (unit increase disadvantage):<br>W 0.88 [0.85-0.92]<br>AA 1.04 [0.93-1.17]
Oliver et al, (2006) (4)	VA, USA	age, PSA-testing status, by ethnicity	RR 0.20 (W), 0.26 (AA)[3,4,5]	RR (D:A) 0.65 (W)[4], 1.15 (AA)[3,5]
Meijer et al, (2013) (5)	Denmark	age, individual socio-demographics	HR 0.95 [0.88-1.02]	HR 0.79 [0.75-0.83][3]
Ocana-Riola et al, (2004) (6)	Granada, Spain	age	RR 0.69 [0.61-0.79][3]	RR 0.73 [0.57-.93]
		Advanced stage prostate cancer *(5 of 16 included studies)*		
Major et al, (2012) (3)	USA	age, individual demographics, lifestyle, by ethnicity	HR W 0.98 [0.79-1.21]<br>AA 2.68 [1.31-5.47]	HR (unit increase disadvantage):<br>W 1.13 [0.79-1.63]<br>AA 0.90 [0.79-1.03]
McLafferty and Wang (2009) (7)	IL, USA	age, ethnicity	OR 0.96 (p> 0.05)	OR (unit increase advantage) 0.92 (p > .05)
Luo et al, (2015) (8)	NSW, Australia	age, year of diagnosis	HR 1.24 [1.14-1.36]	HR 1.12 [1.04-1.19]
Tervonen et al, (2016) (9)	NSW, Australia	age, country of birth, diagnostic period	OR 1.21 [0.88-1.66]	OR 1.33 [1.23-1.45]

Author, year	Location	Additional covariates[1]	Findings [95% confidence interval in brackets][2] Rural versus Urban	Disadvantaged versus Affluent
Haynes et al, (2008) (10)	New Zealand	age, ethnicity	OR 0.61 (p < 0.01)	OR 1.28 (p > 0.05)
	Overall survival *(higher mortality risk is poorer survival, 6 of 6 included studies)*			
Chen et al, (2016) (11)	USA	age, stage, treatment, comorbidity, ethnicity, individual socio-demographics	HR 1.03 [0.86-1.26]	HR income 1.23 [1.1-1.37][3], education 1.22 [1.09-1.37][3]
Marsh et al, (2018) (12)	USA	age, stage, treatment, comorbidity, ethnicity, individual socio-demographics, treatment faculty type	HR 0.97 [0.89-1.05][3]	HR income 1.26 [1.19-1.34], education 1.14 [1.07-1.21]
Prasad et al, (2014) (13)	USA	age, treatment, comorbidity, ethnicity, individual socio-demographics, by PC risk groups[6]	HR: low 0.98 [0.76-1.25], intermediate 1.09 [0.93-1.28], high 0.91 [0.90-1.03]	HR low 1.13 [0.84-1.50], intermediate 1.52 [1.25-1.85], high 1.16 [1.02-1.33]
Vetterlein et al, (2017) (14)	USA	age, stage, treatment, comorbidity, ethnicity, individual socio-demographics, treatment faculty type	HR 0.99 [0.94-1.04]	HR income 1.28 [1.23-1.34], education 1.13 [1.09-1.18]
Jones et al, (2008a) (15)	Northern England	age, treatment faculty type	HR (unit increase travel time) 1.04 [1.01-1.07]	HR (unit increase disadvantage) 1.06 [1.05-1.07]
Hall et al, (2005) (16)	WA, Australia	age, treatment, comorbidity, diagnostic period, ethnicity, individual socio-demographics, treatment faculty type	HR 0.71 [0.36-1.07]	HR 1.34 [1.10-1.64]
	Net survival *(higher mortality risk is poorer survival, 7 of 11 included studies)*			
Yu et al, (2014a) (17)	NSW, Australia	age, stage, 'at-risk' period (people alive, at risk of death)	RER 1.32 [1.19-1.46]	RER 1.40 [1.29-1.53]
White et al, (2011) (18)	TX, USA	age, stage, ethnicity, year of diagnosis	HR 0.95 [0.79-1.14]	HR 1.36 [1.25-1.49]
Li et al, (2012) (19)	Sweden	age, comorbidity, individual socio-demographics	OR 1.26 [1.19-1.35]	OR 1.19 [1.10-1.29]
Campbell et al, (2000) (20)	Scotland	age	HR 1.23 [1.02-1.48]	No modelled estimates
Tervonen et al, (2017a) (21)	NSW, Australia	age, stage, diagnostic period, country of birth	SHR 1.10 [1.05-1.16]	SHR 1.16 [1.11–1.22]
Thomas et al, (2017) (22)	QLD, Australia	age, stage, treatment, individual socio-demographics	SHR 1.21 [0.72-2.05]	SHR 1.35 [0.95-1.92]
Papa et al, (2014) (23)	Victoria, Australia	age, stage, treatment faculty type, only men who had RP	SHR 4.09 [1.56-10.7]	SHR 1.11 [0.55-2.22][3]
	Access and use of services *(10 of 22 included studies, 4 reported two measures)*			
	Higher surgery			
des Bordes et al, (2018) (24)	TX, USA	age, tumour grade, ethnicity, year of diagnosis, only localised PC[7]	OR 0.94 [0.75-1.19]]	OR 0.85 [0.79-0.90]

Author, year	Location	Additional covariates[1]	Findings [95% confidence interval in brackets][2]	
			**Rural versus Urban**	**Disadvantaged versus Affluent**
Jones et al, (2008b) (25)	Northern England	age	OR 0.93 [0.85-1.01]	OR (unit increase disadvantage) 0.99 [0.98-0.99]
Hayen et al, (2008) (26)	NSW, Australia	age, stage, year of diagnosis	RR 0.69 [0.65-0.73]	RR 0.83 [0.78-0.89]
Hall et al, (2005) (16)	WA, Australia	age, comorbidity, diagnostic period, ethnicity, individual socio-demographics, treatment faculty type	HR 0.54 [0.29-1.03]	HR 0.63 [0.47-0.83]
		Higher radiotherapy		
Cobran et al, (2016) (27)	USA	age, stage, tumour grade, comorbidity, year of radiation, marital status, only localised PC[7]	OR 0.17 [0.08-0.37]	OR 0.44 [0.36-0.53][3]
des Bordes et al, (2018) (24)	TX, USA	age, tumour grade, ethnicity, year of diagnosis, only localised PC[7]	OR 1.16 [0.94-1.43]	OR (D:A) 0.65 [0.60-0.69]
Jones et al, (2008b) (25)	Northern England	age	OR 0.88 [0.79-0.99]	OR (unit increase disadvantage) 0.99 [0.98-0.99]
		Type of curative treatment (surgery versus radiotherapy)		
Cary et al, (2016) (28)	USA	age, tumour characteristics, ethnicity, marital status	OR 1.02 [0.87-1.20][3]	Not reported
		Higher any curative treatment (surgery, radiotherapy)		
Baldwin et al, (2013) (29)	USA	age, tumour characteristics, ethnicity, marital status, only early stage PC[7]	HR 0.75 [0.68-0.83][6]	HR 0.75 [0.64-0.88]
Cary et al, (2016) (28)	USA	age, tumour characteristics, ethnicity, marital status	OR 0.81 [0.69-0.95][6]	Not reported
Mahal et al, (2015) (30)	USA	age, ethnicity, marital status, PSA level, only very-low risk PC[6]	OR 1.08 [1.01-1.16][6]	OR 0.80 [0.76-0.85][3]
		Higher hormonal therapy		
Hayen et al, (2008) (26)	NSW, Australia	age, stage, year of diagnosis	RR 1.36 [1.26-1.47]	RR 1.30 [1.15-1.46]
		Access to care		
Aggarwal et al, (2017) (31)	England	age, comorbidity, treatment faculty characteristics	OR 1.87 [1.51-2.33]	OR 0.76 [0.62-0.95][3]
Aggarwal et al, (2018) (32)	England	age, comorbidity, treatment faculty characteristics	OR 2.14 [1.84-2.47]	OR 0.74 [0.52-0.87][3]
		Prostate cancer mortality*(2 of 18 included studies)*		
Hagedoorn et al, (2018) (33)	Belgium	age, individual socio-demographics	RR 1.02 (p> 0.05)	RR 0.97 (p> 0.05)
Odisho et al, (2010) (34)	USA	age, ethnicity, health services	Urban 8.06% lower mortality [-10.94, -5.18][4]	(per unit increase advantage) 0.38 % lower mortality [-0.64, -0.12][4]

2 References

1. McAlister FA, Lin M, Bakal J, Dean S. Frequency of low-value care in Alberta, Canada: a retrospective cohort study. BMJ Qual Saf (2018) 27(5):340-6 doi 10.1136/bmjqs-2017-006778.

2. Littlejohns TJ, Travis RC, Key TJ, Allen NE. Lifestyle factors and prostate-specific antigen (PSA) testing in UK Biobank: Implications for epidemiological research. Cancer Epidemiol (2016) 45:40-6 doi 10.1016/j.canep.2016.09.010.

3. Major JM, Norman Oliver M, Doubeni CA, Hollenbeck AR, Graubard BI, Sinha R. Socioeconomic status, healthcare density, and risk of prostate cancer among African American and Caucasian men in a large prospective study. Cancer Causes Control (2012) 23(7):1185-91 doi 10.1007/s10552-012-9988-8.

4. Oliver MN, Smith E, Siadaty M, Hauck FR, Pickle LW. Spatial analysis of prostate cancer incidence and race in Virginia, 1990-1999. Am J Prev Med (2006) 30(2 Suppl):S67-76 doi 10.1016/j.amepre.2005.09.008.

5. Meijer M, Bloomfield K, Engholm G. Neighbourhoods matter too: the association between neighbourhood socioeconomic position, population density and breast, prostate and lung cancer incidence in Denmark between 2004 and 2008. J Epidemiol Community Health (2013) 67(1):6-13 doi 10.1136/jech-2011-200192.

6. Ocana-Riola R, Sanchez-Cantalejo C, Rosell J, Sanchez-Cantalejo E, Daponte A. Socio-economic level, farming activities and risk of cancer in small areas of Southern Spain. Eur J Epidemiol (2004) 19(7):643-50.

7. McLafferty S, Wang F. Rural reversal? Rural-urban disparities in late-stage cancer risk in Illinois. Cancer (2009) 115(12):2755-64 doi 10.1002/cncr.24306.

8. Luo Q, Yu XQ, Smith DP, O'Connell DL. A population-based study of progression to metastatic prostate cancer in Australia. Cancer Epidemiol (2015) 39(4):617-22 doi 10.1016/j.canep.2015.04.013.

9. Tervonen HE, Walton R, Roder D, You H, Morrell S, Baker D, et al. Socio-demographic disadvantage and distant summary stage of cancer at diagnosis--A population-based study in New South Wales. Cancer Epidemiol (2016) 40(2):87-94 doi 10.1016/j.canep.2015.10.032.

10. Haynes R, Pearce J, Barnett R. Cancer survival in New Zealand: ethnic, social and geographical inequalities. Soc Sci Med (2008) 67(6):928-37 doi 10.1016/j.socscimed.2008.05.005.

11. Chen Y-W, Mahal BA, Muralidhar V, Nezolosky M, Beard CJ, Den RB, et al. Association Between Treatment at a High-Volume Facility and Improved Survival for Radiation-Treated Men With High-Risk Prostate Cancer. Int J Radiat Oncol Biol Phys (2016) 94(4):683-90 doi 10.1016/j.ijrobp.2015.12.008.

12. Marsh S, Walters RW, Silberstein PT. Survival Outcomes of Radical Prostatectomy Versus Radiotherapy in Intermediate-Risk Prostate Cancer: A NCDB Study. Clin Genitourin Cancer (2018) 16(1):E39-E46 doi 10.1016/j.clgc.2017.07.029.

13. Prasad SM, Eggener SE, Lipsitz SR, Irwin MR, Ganz PA, Hu JC. Effect of depression on diagnosis, treatment, and mortality of men with clinically localized prostate cancer. J Clin Oncol (2014) 32(23):2471-8 doi 10.1200/jco.2013.51.1048.

14. Vetterlein MW, Loppenberg B, Karabon P, Dalela D, Jindal T, Sood A, et al. Impact of travel distance to the treatment facility on overall mortality in US patients with prostate cancer. Cancer (2017) 123(17):3241-52 doi 10.1002/cncr.30744.

15. Jones AP, Haynes R, Sauerzapf V, Crawford SM, Zhao H, Forman D. Travel times to health care and survival from cancers in Northern England. Eur J Cancer (2008) 44(2):269-74 doi 10.1016/j.ejca.2007.07.028.

16. Hall SE, Holman CD, Wisniewski ZS, Semmens J. Prostate cancer: socio-economic, geographical and private-health insurance effects on care and survival. BJU Int (2005) 95(1):51-8 doi 10.1111/j.1464-410X.2005.05248.x.

17. Yu XQ, Luo Q, Smith DP, O'Connell DL, Baade PD. Geographic variation in prostate cancer survival in New South Wales. Med J Aust (2014) 200(10):586-90.

18. White A, Coker AL, Du XL, Eggleston KS, Williams M. Racial/ethnic disparities in survival among men diagnosed with prostate cancer in Texas. Cancer (2011) 117(5):1080-8 doi 10.1002/cncr.25671.

19. Li X, Sundquist K, Sundquist J. Neighborhood deprivation and prostate cancer mortality: a multilevel analysis from Sweden. Prostate Cancer Prostatic Dis (2012) 15:128 doi 10.1038/pcan.2011.46.

20. Campbell NC, Elliott AM, Sharp L, Ritchie LD, Cassidy J, Little J. Rural factors and survival from cancer: analysis of Scottish cancer registrations. Br J Cancer (2000) 82(11):1863-6 doi 10.1054/bjoc.1999.1079.

21. Tervonen HE, Aranda S, Roder D, You H, Walton R, Morrell S, et al. Cancer survival disparities worsening by socio-economic disadvantage over the last 3 decades in new South Wales, Australia. BMC Public Health (2017) 17(1):691 doi 10.1186/s12889-017-4692-y.

22. Thomas AA, Pearce A, Sharp L, Gardiner RA, Chambers S, Aitken J, et al. Socioeconomic disadvantage but not remoteness affects short-term survival in prostate cancer: A population-based study using competing risks. Asia Pac J Clin Oncol (2017) 13(2):e31-e40 doi 10.1111/ajco.12570.

23. Papa N, Lawrentschuk N, Muller D, MacInnis R, Ta A, Severi G, et al. Rural residency and prostate cancer specific mortality: results from the Victorian Radical Prostatectomy Register. Aust N Z J Public Health (2014) 38(5):449-54 doi 10.1111/1753-6405.12210.

24. des Bordes JKA, Lopez DS, Swartz MD, Volk RJ. Sociodemographic Disparities in Cure-Intended Treatment in Localized Prostate Cancer. J Racial Ethn Health Disparities (2018) 5(1):104-10 doi 10.1007/s40615-017-0348-y.

25. Jones AP, Haynes R, Sauerzapf V, Crawford SM, Zhao H, Forman D. Travel time to hospital and treatment for breast, colon, rectum, lung, ovary and prostate cancer. Eur J Cancer (2008) 44(7):992-9 doi 10.1016/j.ejca.2008.02.001.

26. Hayen A, Smith DP, Patel MI, O'Connell DL. Patterns of surgical care for prostate cancer in NSW, 1993-2002: rural/urban and socio-economic variation. Aust N Z J Public Health (2008) 32(5):417-20 doi 10.1111/j.1753-6405.2008.00272.x.

27. Cobran EK, Chen RC, Overman R, Meyer AM, Kuo TM, O'Brien J, et al. Racial Differences in Diffusion of Intensity-Modulated Radiation Therapy for Localized Prostate Cancer. Am J Mens Health (2016) 10(5):399-407 doi 10.1177/1557988314568184.

28. Cary C, Odisho AY, Cooperberg MR. Variation in prostate cancer treatment associated with population density of the county of residence. Prostate Cancer Prostatic Dis (2016) 19:174 doi 10.1038/pcan.2015.65.

29. Baldwin LM, Andrilla CH, Porter MP, Rosenblatt RA, Patel S, Doescher MP. Treatment of early-stage prostate cancer among rural and urban patients. Cancer (2013) 119(16):3067-75 doi 10.1002/cncr.28037.

30. Mahal BA, Cooperberg MR, Aizer AA, Ziehr DR, Hyatt AS, Choueiri TK, et al. Who bears the greatest burden of aggressive treatment of indolent prostate cancer? Am J Med (2015) 128(6):609-16 doi 10.1016/j.amjmed.2014.12.030.

31. Aggarwal A, Lewis D, Sujenthiran A, Charman SC, Sullivan R, Payne H, et al. Hospital Quality Factors Influencing the Mobility of Patients for Radical Prostate Cancer Radiation Therapy: A National Population-Based Study. Int J Radiat Oncol Biol Phys (2017) 99(5):1261-70 doi 10.1016/j.ijrobp.2017.08.018.

32. Aggarwal A, Lewis D, Charman SC, Mason M, Clarke N, Sullivan R, et al. Determinants of Patient Mobility for Prostate Cancer Surgery: A Population-based Study of Choice and Competition. Eur Urol (2018) 73(6):822-5 doi 10.1016/j.eururo.2017.07.013.

33. Hagedoorn P, Vandenheede H, Vanthomme K, Gadeyne S. Socioeconomic position, population density and site-specific cancer mortality: A multilevel analysis of Belgian adults, 2001-2011. Int J Cancer (2018) 142(1):23-35 doi 10.1002/ijc.31031.

34. Odisho AY, Cooperberg MR, Fradet V, Ahmad AE, Carroll PR. Urologist density and county-level urologic cancer mortality. J Clin Oncol (2010) 28(15):2499-504 doi 10.1200/jco.2009.26.9597.
